# Loss of miR-514a-3p regulation of PEG3 activates the NF-kappa B pathway in human testicular germ cell tumors

**DOI:** 10.1038/cddis.2016.464

**Published:** 2017-05-04

**Authors:** Deniz Mahmut Özata, Xidan Li, Linkiat Lee, Jikai Liu, Dudi Warsito, Praveensingh Hajeri, Isabell Hultman, Omid Fotouhi, Stefan Marklund, Lars Ährlund-Richter, Carl Christofer Juhlin, Catharina Larsson, Weng-Onn Lui

**Affiliations:** 1 Department of Oncology-Pathology, Karolinska Institutet, Stockholm, Sweden; 2 Cancer Center Karolinska, Karolinska University Hospital, Stockholm, Sweden; 3 Swedish University of Agricultural Sciences, Uppsala, Sweden; 4 Department of Urology, Jinshan Hospital of Fudan University, Shanghai, China; 5 Department of Surgery, University of Minnesota, Minneapolis, MN, USA; 6 Department of Women’s and Children’s Health, Karolinska Institutet, Stockholm, Sweden

## Abstract

Deregulation of microRNAs (miRNAs) contributes to the development and progression of many cancer types; however, their functions in the pathogenesis of testicular germ cell tumor (TGCT) remain unclear. Here, we determined miRNA expression profiles of TGCTs and normal testes using small RNA sequencing, and identified several deregulated miRNAs in TGCTs, including the miR-506~514 cluster. In functional studies *in vitro* we demonstrated that miR-514a-3p induced apoptosis through direct regulation of the paternally expressed gene 3 (PEG3), and ectopically expressed PEG3 could rescue the apoptotic effect of miR-514a-3p overexpression. Silencing of PEG3 or miR-514a-3p overexpression reduced nuclear accumulation of p50 and NF-*κ*B reporter activity. Furthermore, PEG3 was co-immunoprecipitated with tumor necrosis factor receptor-associated factor 2 (TRAF2) in TGCT cell lysates. We propose a model of PEG3-mediated activation of NF-*κ*B in TGCT. Loss of miR-514a-3p expression in TGCT increases PEG3 expression that recruits TRAF2 and activates the NF-kappa B pathway, which protects germ cells from apoptosis. Importantly, we observed strong expression of PEG3 and nuclear p50 in the majority of TGCTs (83% and 78%, respectively). In conclusion, our study describes a novel function for miR-514a-3p in TGCT and highlights an unrecognized mechanism of PEG3 regulation and NF-*κ*B activation in TGCT.

Testicular germ cell tumor (TGCT) is the most common solid malignancy occurring in young men between 20 and 34 years of age, and its incidence has increased significantly over the last decades.^1^ The disease may be successfully treated with cisplastin-based chemotherapy, to which ~90% of TGCTs are sensitive. However, the treatment also increases the risk of developing secondary cancers and cardiovascular disease.^2, 3^

TGCT is a developmental disease of germ cell differentiation, and almost all TGCTs are derived from dysfunctional fetal germ cells known as carcinoma *in situ* (CIS)^4^ or intratubular germ cell neoplasia unclassified (IGCNU, WHO classification).^5^ These cells resemble primordial germ cells with expression of common pluripotency/germ cell markers (e.g., KIT, NANOG, OCT3/4, VASA, AP)^6, 7, 8^ and lack of imprinting.^9^ The development of IGCNU involves activation of the KITLG/SCF pathway and overexpression of embryonic transcription factors such as POU5F1, NANOG, STELLAR and GDF3, which lead to increased cell proliferation, suppression of apoptosis and accumulation of mutations.^10^ Although the progression of IGCNU to invasive tumors is still poorly understood, loss of PTEN and gain of chromosomal region 12p are associated with invasive TGCTs.^11, 12^

Genome-wide linkage analyses have identified several candidate genetic loci for predisposition to TGCT. The first locus was mapped to chromosomal region Xq27;^13^ however, the putative gene is yet to be discovered. Subsequently, several additional susceptibility loci have been reported, including three that overlap with the locations of *KITLG*, *SPRY4* and *BAK1*, involved in KIT signaling pathways regulating proliferation, survival and migration of primordial germ cells or TGCT development.^14^

Although molecular studies have contributed to our understanding of the etiology of TGCT, the role of microRNAs (miRNAs) in this tumor type is not fully understood. miRNAs are ~22-nucleotide-long single-stranded non-coding RNAs, which have important roles in the regulation of gene expression of many physiological and pathological processes, including tumor development.^15^ Several observations highlight the importance of miRNAs in TGCT development. For example, miR-372 and miR-373 act as oncogenes in TGCT by repressing the tumor suppressor activity of LATS2,^16^ and distinct miRNA expression profiles are associated with cell lineage and differentiation status of TGCT subtypes.^17^

In this study, we applied a deep-sequencing approach to comprehensively characterize miRNA profiles in human TGCTs and normal testes (NT). We identified several differentially expressed miRNAs in TGCTs, including the miR-506~514 cluster. This cluster is located in chromosomal region Xq27.3, which is a susceptibility locus for TGCT. Functionally, we demonstrated that miR-514a-3p, a member of the miR-506~514 cluster, protects cells from apoptosis by activation of NF-*κ*B through PEG3. The findings suggest an important role for PEG3 and NF-*κ*B in TGCT development.

## Results

### Identification of novel candidate miRNAs in TGCTs and NT

We sequenced small RNA (sRNA) libraries prepared from nine testicular germ cell tumors (TGCTs) and two NTs. A total of 3 656 505 sequencing reads were obtained from 11 sRNA libraries (NT: 1 694 962 reads; TGCTs: 1 961 543 reads). The workflow of the sRNA-sequencing data analysis is depicted in Supplementary Figure 1, together with a summary of the sRNA compositions in both NT and TGCTs. Besides the annotated RNA sequences, a total of 37 487 reads were mapped to the human genome but did not correspond to any known RNA species. Using previously described criteria for candidate miRNAs,^18^ we identified 29 novel candidate miRNA genes (Supplementary Table 1).

### Differentially expressed miRNAs between TGCTs and NT

**Figure 1:**
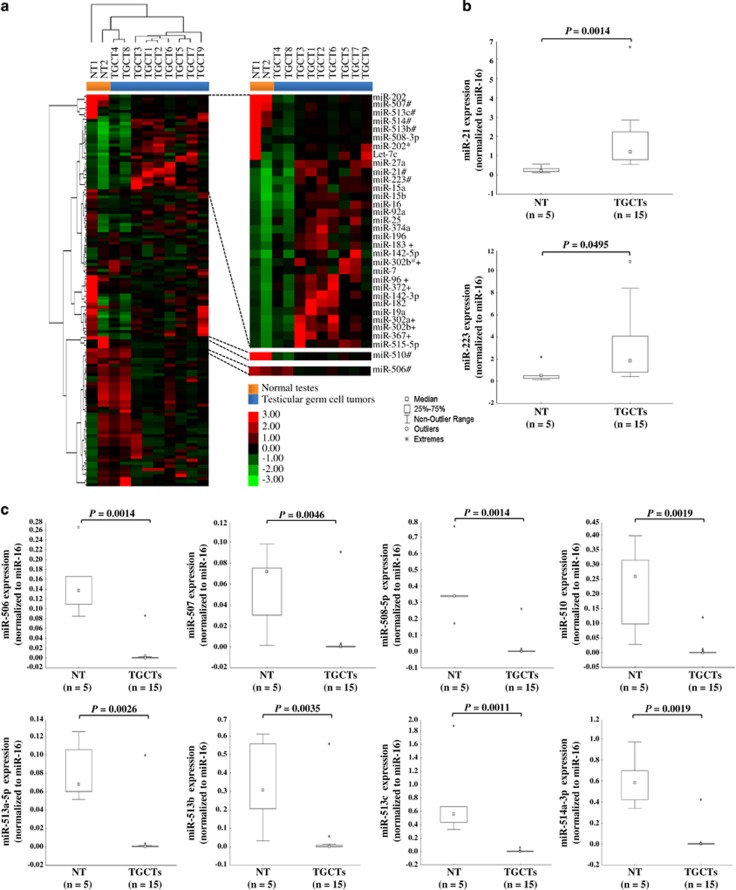
miRNA expression profiles and validation of deregulated miRNAs in TGCTs and NT. (**a**) Heatmap illustrating miRNA expression profiles obtained from sRNA-sequencing experiments of two NT (orange) and nine TGCTs (blue). Genes and samples were clustered using Euclidean distance and complete linkage. To the right is the enlargement of subsets of miRNAs. Red and green colors indicate relatively high and low expression, respectively. *refers to passenger miRNAs based on miRBase release 16; ^#^indicates miRNAs selected for RT-qPCR validation; ^+^refers to previously reported deregulated miRNAs in TGCTs.^16, 17^ (**b** and **c**) Validation of differentially expressed miRNAs between TGCTs (*n*=15) and NT (*n*=5) by RT-qPCR. The boxplots illustrate the relative expression of individual miRNAs normalized to miR-16. The expressions of miR-21 and miR-223 were significantly higher (**b**), whereas the members of the miR-506~514 cluster were lower (**c**), in TGCTs compared with NT. Statistical significance of the data was calculated with the Mann–Whitney *U*-test. *P*<0.05 was considered significant

To identify deregulated miRNAs in TGCTs, we compared the relative incidence frequency of each miRNA between TGCTs and NT. The analysis revealed 263 differentially expressed miRNAs between TGCTs and NT (*P*<0.0001; Supplementary Table 2). Using significance analysis of microarrays (SAM), we found 23 overexpressed and 105 underexpressed miRNAs in TGCTs as compared with NT (Supplementary Table 3). Clustering analysis of these 128 miRNAs showed that the majority of the TGCTs (seven out of nine) were grouped together but separately from the two NT (Figure 1a).

To verify the sequencing data, we selected 10 miRNAs for validation in an extended cohort of clinical samples (15 TGCTs and 5 NT) using reverse transcription-quantitative PCR. These miRNAs were selected based on their score from the SAM analysis (Supplementary Table 3), or their involvement in other tumor types.^19, 20, 21^ In concordance with the sequencing data, all 10 miRNAs were significantly differentially expressed between TGCTs and NT (Figures 1b and c). miR-21 and miR-223 expression levels were increased in TGCTs, whereas the eight miRNAs in the miR-506~514 cluster (miR-506, miR-507, miR-508-5p, miR-510, miR-513a-5p, miR-513b, miR-513c and miR-514a-3p) were reduced in TGCTs as compared with NT.

### Functional studies of miR-510 and miR-514a-3p in TGCT cell lines

**Figure 2:**
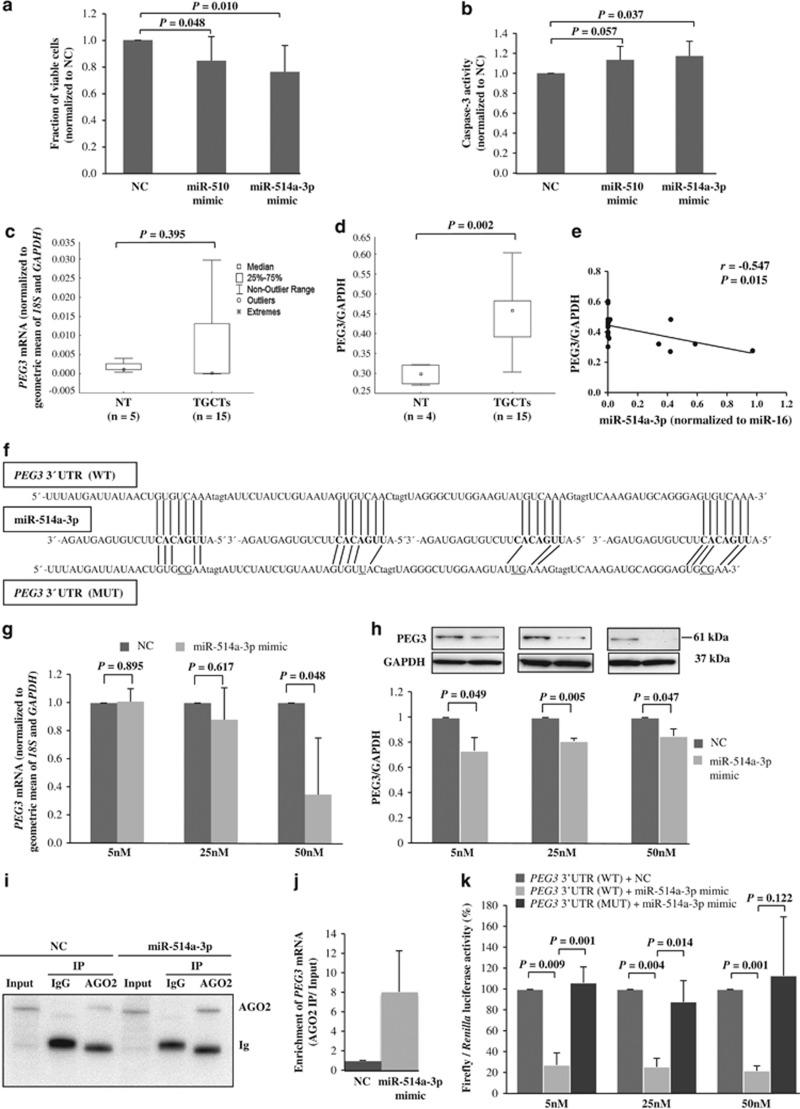
Functional studies and evaluation of PEG3 as a target of miR-514a-3p in TCam-2 cells. (**a** and **b**) The effect of miR-510 or miR-514a-3p overexpression on cell proliferation was assessed by WST-1 cell proliferation assay (*n*=9; **a**) and apoptosis was determined by caspase-3 calorimetric assay (*n*=6; **b**). (**c** and **d**) Evaluation of PEG3 mRNA and protein levels in NT and TGCTs by RT-qPCR (**c**) and western blot analysis (**d**), respectively. Western blot images of Figure 2d are shown in Supplementary Figure 3. (**e**) Correlation between PEG3 and miR-514a-3p expressions was evaluated using Pearson’s correlation analysis. (**f**) Illustration of sequence alignment of miR-514a-3p and the wild-type (WT) and the mutated (MUT) target sequences of *PEG3*. The seed sequence of miR-514a-3p is in bold, and the mutated sequences are underlined. (**g** and **h**) PEG3 mRNA and protein expressions were quantified in TCam-2 cells transfected with different concentrations of miR-514a-3p mimic or negative control (NC, miRNA mimic negative control #1) using RT-qPCR (**g**) and western blot analysis (**h**), respectively. Top: representative western blots showing decreased expression of PEG3 upon miR-514a-3p overexpression. Bottom: quantification of PEG3 protein levels in three independent experiments. (**i**) AGO2-IP was performed on protein lysates from TCam-2 cells transfected with NC or miR-514a-3p mimic using anti-AGO2 or -IgG (isotype control). Immunoprecipitates were analyzed by immunoblotting with anti-AGO2 antibody. Input represents 2% protein lysates used for IP. Ig, Ig heavy chain. (**j**) RT-qPCR analysis of *PEG3* mRNA was evaluated in AGO2-IP mRNAs of miR-514a-3p-overexpressing cells as compared with NC-treated cells. The geometric mean of miR-372 and miR-373 was used as endogenous controls for AGO2-IP RNA. Fold change was calculated by dividing the normalized expression values of AGO2-IP samples by the normalized expression values of its respective input samples. (**k**) The effect of miR-514a-3p on luciferase activity was evaluated 48 h after co-transfection of miR-514a-3p mimic or NC with the WT and MUT of *PEG3* reporter constructs in TCam-2 cells. Error bars represent standard deviations (S.D.) of the mean of at least three independent experiments. *P*-values were calculated by Student's paired *t*-test

The finding of decreased expression for the miR-506~514 cluster in TGCTs prompted us to investigate their functional consequences in TGCT cell lines. Two miRNAs in this cluster (miR-510 and miR-514a-3p) were chosen for functional studies because of their higher read counts as compared with the other members of the miR-506~514 cluster. Using the WST-1 cell viability assay, we showed that overexpression of miR-510 or miR-514a-3p in TCam-2 cells reduced cell viability (15% and 24%, respectively; *P*<0.05) as compared with the NC-treated cells (Figure 2a). Overexpression of miR-510 or miR-514a-3p also induced apoptosis (13% and 17%, respectively), as demonstrated by the caspase-3 activity assay (Figure 2b). A similar effect was observed in the 2102Ep cell line where Poly-ADP ribose polymerase (PARP) cleavage was increased by 30% in the cells transfected with miR-514a-3p mimic compared with the NC (*P=*0.031; Supplementary Figure 2). Because of the more pronounced functional effects observed for miR-514a-3p, we further investigated its direct target and its role in the pathogenesis of TGCT.

### Identification and validation of *PEG3* as a direct target of miR-514a-3p

We applied miRNA target prediction tools to identify candidate targets of miR-514a-3p. The paternally expressed gene 3 (PEG3) was ranked top as a predicted target of miR-514a-3p with three conserved and two poorly conserved sites using TargetScanHuman (release 6.2; http://www.targetscan.org). Furthermore, it was the fourth highest-ranked target of miR-514a-3p by miRanda (http://www.microrna.org/microrna/home.do). To investigate whether *PEG3* could be a target of miR-514a-3p, we compared the gene and protein expression levels in TGCTs and NT. We found that the PEG3 protein level, but not the mRNA level, was increased in TGCTs compared with NT (*P=*0.002; Figures 2c, d and Supplementary Figure 3) and inversely correlated with miR-514a-3p expression (*r*=−0.547, *P*=0.015; Figure 2e).

To determine the effect of miR-514a-3p on PEG3 expression, we quantified PEG3 expression by RT-qPCR and western blot analyses in TCam-2 and 2102Ep cells upon transfection of miR-514a-3p. We observed dosage-dependent decreases of PEG3 mRNA and protein levels in TCam-2 cells overexpressing miR-514a-3p (Figures 2g and h). Concordantly, reduced PEG3 mRNA and protein levels were also observed in 2102Ep cells transfected with miR-514a-3p mimic (Supplementary Figure 2).

Two different approaches were applied to determine whether *PEG3* is directly regulated by miR-514a-3p. First, we quantified *PEG3* mRNA levels by RT-qPCR after argonaute 2 immunoprecipitation (AGO2-IP) of TCam-2 cells transfected with miR-514a-3p mimic or negative control. We observed an enrichment of *PEG3* mRNA in the cells with miR-514a-3p overexpression compared with the control (Figure 2j). Second, we performed luciferase reporter assays to examine whether miR-514a-3p could directly target the 3’UTR of *PEG3*. Four miR-514a-3p target sites (sites 1–4, Supplementary Figure 4) were inserted into the 3’UTR of a luciferase reporter vector. TCam-2 cells were co-transfected with the wild-type (WT) *PEG3* 3’UTR construct and miR-514a-3p mimic or negative control. Significant reductions of luciferase activity were observed in the cells overexpressing miR-514a-3p compared with miRNA mimic negative controls (more than threefolds and *P*<0.01 for all three different concentrations of miR-514a-3p mimic; Figure 2k). As a complementary strategy to assess the interaction between miR-514a-3p and *PEG3* 3’UTR, we included a seed-mutant (MUT) construct, which has two to three mismatches in the seed region of the target sites (Figure 2f). The seed-MUT construct completely abolished the suppression of luciferase activity by miR-514a-3p (Figure 2k).

### Quantification of promoter methylation density for *PEG3* in TGCTs and NT

Given that the *PEG3* promoter resides within a CpG-rich region that is differentially methylated in cancers,^22, 23^ we asked whether increased expression of PEG3 in TGCTs could be due to loss of its promoter methylation. Here, we quantified the methylation density at five CpG sites in the *PEG3* promoter using bisulfite pyrosequencing. The analysis revealed comparable methylation levels for all five CpG sites in TGCTs (mean MetI 39% range 1–100%) and NT (mean MetI 39% range 16–65% Supplementary Figure 4), suggesting that increased expression of PEG3 in TGCTs is not due to loss of methylation in the *PEG3* promoter.

### Increased apoptosis after PEG3 silencing in TGCT cells

**Figure 3:**
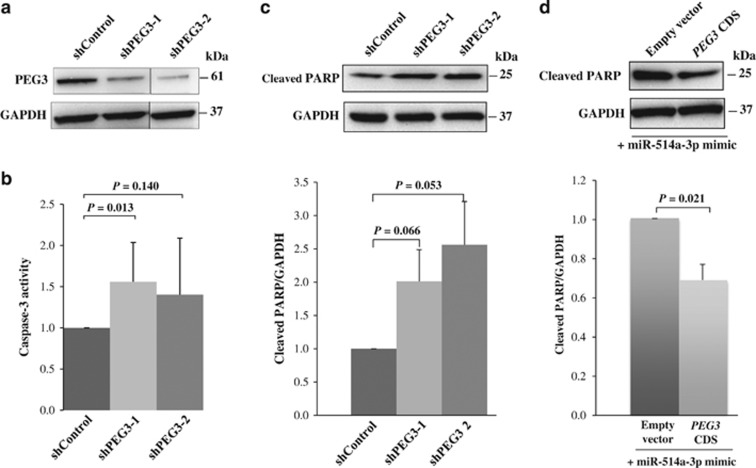
PEG3 regulates apoptosis in TCam-2 cells. (**a**) Detection of PEG3 protein expression in cells transfected with short hairpin RNA against PEG3 (shPEG3-1 or shPEG3-2) or vector control (shControl) by western blot analysis. (**b** and **c**) Evaluation of the effect of PEG3 silencing on apoptosis using caspase-3 activity (*n*=8; **b**) and cleaved PARP (*n*=3; **c**) assays. (**d**) Western blot analysis of cleaved PARP in cells co-transfected with miR-514a-3p and *PEG3* full-length coding sequence without 3’ UTR (CDS) or vector control. GAPDH was used as a loading control. Error bars represent S.D. of the mean of at least three independent experiments. Differences between two groups were evaluated using paired *t*-test and *P*<0.05 as significant

PEG3 is known to have both pro-apoptotic^24^ and anti-apoptotic^25^ roles in different cell types. Given that PEG3 protein expression was significantly higher in TGCTs as compared with NT, we hypothesized that PEG3 promotes cell survival by preventing apoptosis in TGCT. To investigate the effect of PEG3 on cell apoptosis, we silenced PEG3 expression using short hairpin RNAs (shRNAs) targeting exon 4 or exon 10 of the *PEG3* gene (designated as shPEG3-1 and shPEG3-2, respectively; Figure 3a and Supplementary Figure 4), and assessed their effects on caspase-3 activity and accumulation of cleaved PARP. Indeed, we observed increases in caspase-3 activity and cleaved PARP upon suppression of PEG3 (Figures 3b and 3c).

Given that miR-514a-3p promotes apoptosis and *PEG3* is a direct target of miR-514a-3p, we tested whether ectopically expressed PEG3 could rescue the miR-514a-3p-mediated apoptotic effect. We co-transfected TCam-2 cells with miR-514a-3p mimic and an expression plasmid encoding the full-length coding sequence of *PEG3* without the 3’UTR region (pCMV6-PEG3-CDS) or a vector control and examined the apoptotic effect by cleaved PARP. As shown in Figure 3d, ectopic expression of PEG3 significantly decreased the apoptotic effect caused by miR-514a-3p overexpression.

### Activation of the NF-*κ*B pathway by PEG3 in TGCT cell lines

**Figure 4:**
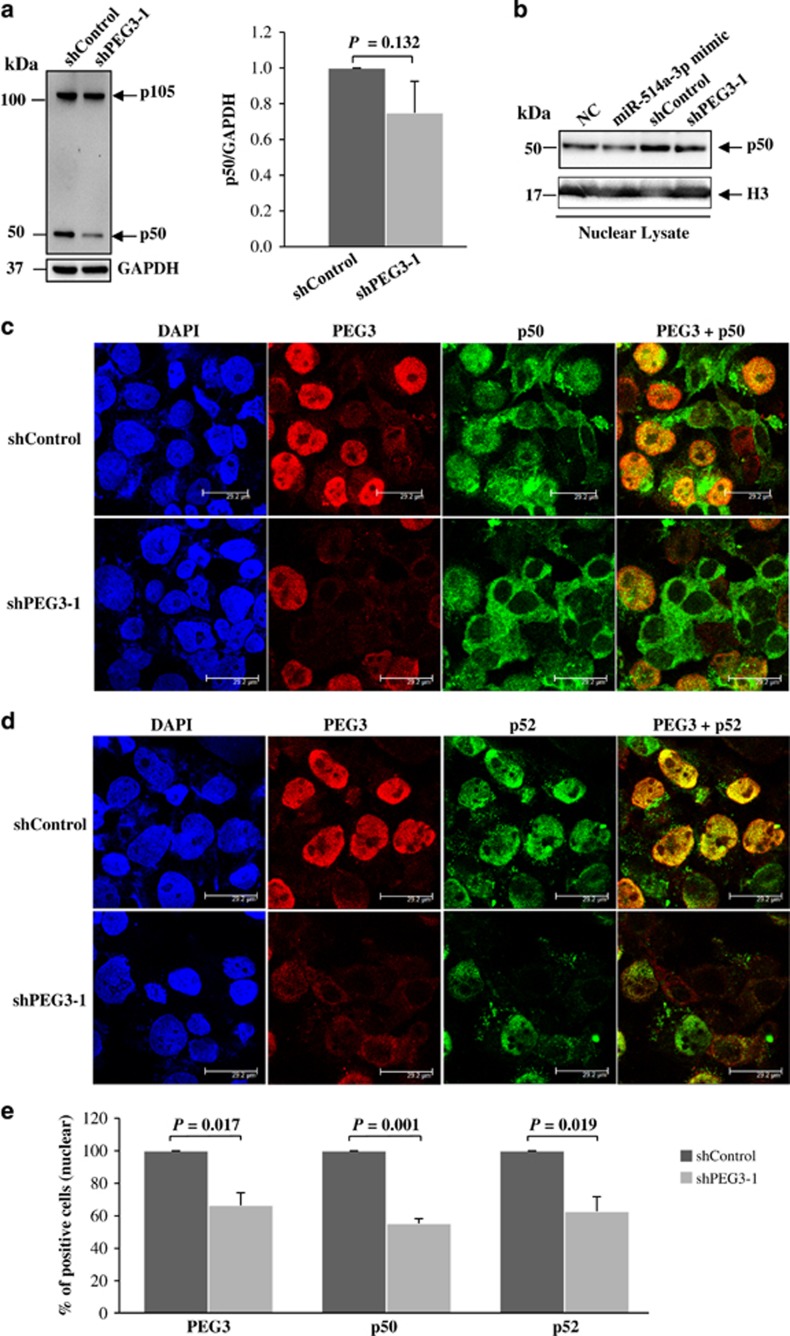
Involvement of PEG3 in the NF-*κ*B pathway. (**a**) Western blot analysis of p105/p50 in the total lysate of TCam-2 cells transfected with shPEG3-1 or shControl. Left: representative western blot of p105/p50. Right: quantification of p50 expression from the western blot analysis. GAPDH was used as a normalization control. (**b**) Western blot analysis of p50 expression in the nuclear lysate of TCam-2 cells transfected with miR-514a-3p or shPEG3-1, and their respective negative controls. (**c** and **d**) Confocal immunofluorescence images of PEG3 (red), p105/p50 (green, **c**), p100/p52 (green, **d**) and DAPI (blue) in TCam-2 cells transfected with shPEG3-1 or shControl. (**e**) Quantification of cells with nuclear expression of PEG3, p50 or p52 detected by immunofluorescence. Error bars represent the S.D. of 500 cells counted from two independent experiments

Given that PEG3 has been shown to protect cells from apoptosis by activation of the NF-*κ*B pathway via interaction with tumor necrosis factor receptor-associated factor 2 (TRAF2),^25^ we evaluated the effects of PEG3 and miR-514a-3p expressions on NF-*κ*B activation. We first silenced PEG3 expression using shPEG3-1 and assessed its effect on the processing of NF-*κ*B precursor p105 to p50 by western blot analysis. As shown in Figure 4a, suppression of PEG3 expression led to a decrease in p105 proteolysis and lower level of p50 as compared with shControl-treated cells. Given that p50 translocates to the nucleus and activates target genes, we quantified p50 expression in the nuclear fractions of TCam-2 cells transfected with miR-514a-3p mimic or shPEG3-1 and their respective negative controls. Reduction of nuclear p50 expression was observed both in cells overexpressing miR-514a-3p and with silencing of PEG3 expression (Figure 4b). Similar results were obtained in 2102Ep cells, in which ectopic expression of miR-514a-3p decreased the accumulation of p50 in the total cell lysate and in the nuclear lysate (Supplementary Figure 2).

NF-*κ*B is activated by two distinct pathways that exclusively involve p50 or p52 in the classical and alternative pathways, respectively.^26^ To determine whether PEG3 contributes to NF-*κ*B activation by the classical and/or alternative pathways, we assessed nuclear expression of p50 and p52 in TCam-2 cells with and without silencing of PEG3 using immunofluorescence microscopy. Our results showed that PEG3 was predominantly expressed in the nucleus and co-localized with nuclear p50 or p52 expressions in the shControl-treated cells (Figures 4c and d). Silencing of PEG3 expression resulted in a significant decrease in nuclear p50 and p52 expression (Figures 4c–e). Together, our data support the involvement of PEG3 in both classical and alternative pathways in TGCT *in vitro*.

**Figure 5:**
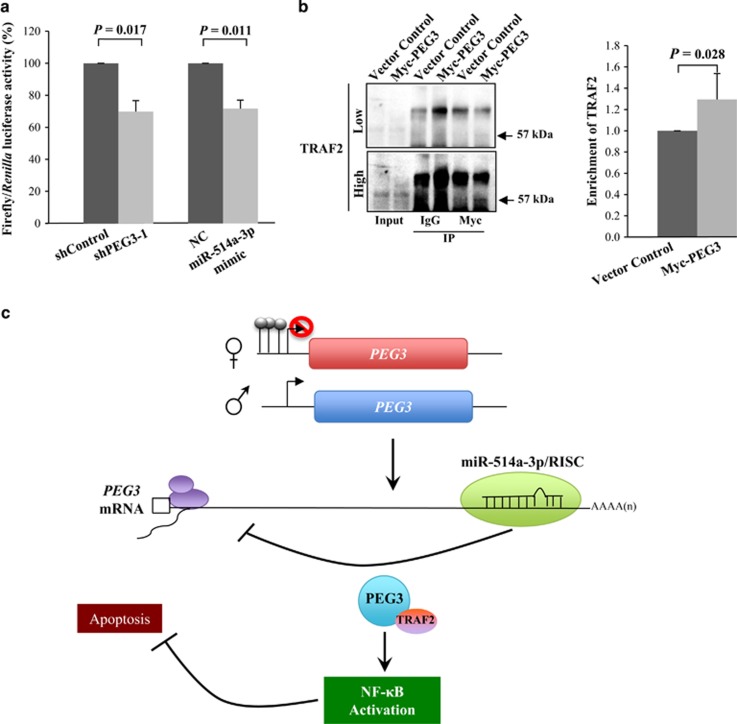
Silencing of PEG3 and miR-514a-3p overexpression suppress NF-*κ*B reporter activity. (**a**) Evaluation of NF-*κ*B luciferase activity in TCam-2 cells co-transfected with NF-*κ*B reporter vector and shPEG3-1 or miR-514a-3p mimic, as compared with their respective negative controls. (**b**) IP was performed on TCam-2 cells expressing Myc-PEG3 or vector control using anti-Myc or -IgG. The immunoprecipitates and input (2%) were analyzed by immunoblotting using anti-TRAF2 antibody. Low and high refers to the same western blot detected at low and high exposure time, respectively. Error bars indicate S.D. of the mean of three independent experiments. (**c**) Model of PEG3-mediated NF-*κ*B activation in TGCT, in which increased expression of PEG3 protect cells from apoptosis by activation of NF-*κ*B

To further demonstrate the functional significance of PEG3 and miR-514a-3p in the activation of the NF-*κ*B pathway, we tested their effects on NF-*κ*B luciferase reporter activity. Suppression of PEG3 and overexpression of miR-514a-3p significantly reduced luciferase activity (>30%, *P=*0.01; Figure 5a). As aforementioned, PEG3 can induce the NF-*κ*B pathway through binding to TRAF2.^25^ We therefore determined whether PEG3 interacted with TRAF2 in TGCT cells, by pulling down PEG3 complexes and measured the enrichment of TRAF2 in the IP lysates via western blotting. We observed a significant enrichment of TRAF2 in the Myc-tagged PEG3 pull-down lysates compared with the vector control (29%, *P*=0.028; Figure 5b). Collectively, our findings suggest a model of PEG3-mediated activation of NF-*κ*B in TGCT: PEG3 overexpression, because of the loss of miR-514a-3p-mediated suppression, recruits TRAF2 to activate the NF-*κ*B pathway that protects germ cells from apoptosis (Figure 5c).

### Expression of PEG3 and p50 in testicular tumors and non-tumorous tissues

**Figure 6:**
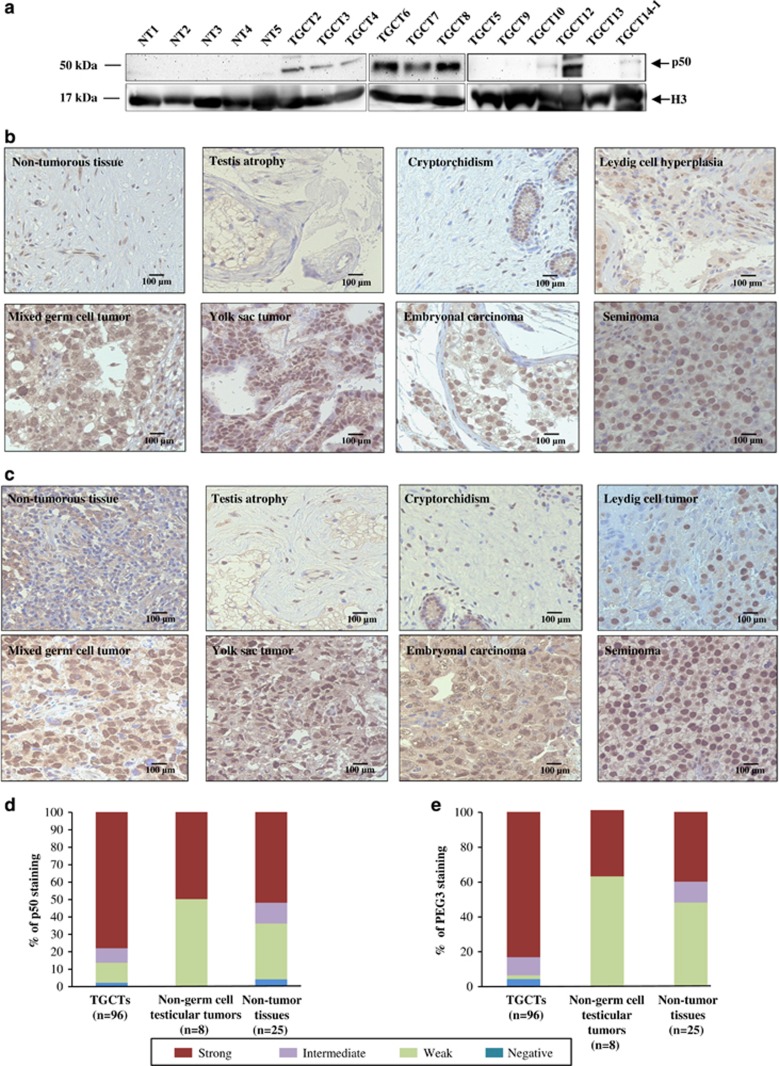
PEG3 and p50 expressions in testicular tumors and non-tumorous tissues. (**a**) Western blot analysis of endogenous p50 expression in the nuclear lysates of NT (*n*=5) and TGCTs (*n*=12). H3 was used as a loading control for nuclear lysates. (**b** and **c**) Representative immunohistochemical staining of p50 (**b**) and PEG3 (**c**) in different TGCT subtypes (lower panels), non-cancerous testicular tissues and other testicular tissues (upper panels). (**d** and **e**) Proportion of cases with positive nuclear staining for p50 (**d**) and PEG3 (**e**) in TGCTs, non-germ cell testicular tumors and testicular non-tumor tissues. Each sample was scored based on the intensity of positive nuclear staining. Strong (red); intermediate (purple); weak (green); negative (blue). The details of the samples and scoring are available in Supplementary Table 4

To further investigate the clinical significance of our findings, we first assessed the endogenous expression of p50 in nuclear lysates of 12 TGCTs and 5 NT by western blotting; three of the TGCTs were excluded because of insufficient protein lysates. Expression of nuclear p50 was evident in 7/12 of the TGCT samples examined, detected at very low or undetectable levels in 5/12 tumors, whereas no expression was detected in the five NT (Figure 6a).

We further evaluated both PEG3 and p50 immunohistochemical expressions in TGCTs, non-germ cell tumors and non-tumorous testicular tissues using tissue microarrays. PEG3 and p50 showed similar expression patterns. Strong nuclear p50 and PEG3 expressions were observed in different subtypes of TGCTs, whereas their expressions were lower in non-germ cell testicular tumors and testicular non-tumor tissues (Figures 6b and c).

For both proteins, we scored the intensity of nuclear-positive staining in each specimen (Figures 6d and e). The number and proportions of tissues with negative, weak, intermediate and strong nuclear staining is detailed in Supplementary Table 4. For PEG3, strong immunohistochemical staining was observed in 80/96 TGCTs (83%), as compared with 3/8 non-germ cell testicular tumors (37%) and 10/25 testicular non-tumor tissues (40%). For p50, strong immunoreactivity was observed in 75/96 TGCTs (78%), 4/8 non-germ cell testicular tumors (50%) and 13/25 testicular non-tumor tissues (52%).

## Discussion

Deregulation of miRNA expressions is known to be involved in testicular germ cell tumorigenesis. Despite distinct miRNA expression signatures are associated with histological subtypes, very few miRNAs have been functionally characterized in TGCT. Here, we describe the identification of a set of deregulated miRNAs in TGCT and the role of miR-514-3p and NF-*κ*B in TGCT.

A striking observation is the loss of miR-506~514 cluster expression in TGCT. This cluster is conserved in primates, and consists of seven distinct miRNAs, that is, miR-506, miR-507, miR-508, miR-509, miR-510, miR-513 and miR-514.^27^ The expression pattern and function of this miRNA cluster is depending on cellular context. In ovarian carcinoma, this miRNA cluster has been demonstrated as a tumor suppressor,^28^ whereas in melanoma it promotes melanoma development and progression.^21^ Downregulation of miR-506~514 cluster was also previously reported in seminomas and embryonal carcinomas as compared to CIS and NT, ^29^ suggesting its important role in TGCT development.

Here, we demonstrate the pro-apoptotic function of miR-514a-3p in TGCT cells by targeting *PEG3*. PEG3 expression is commonly reduced or lost in cancer cell lines and tumors because of promoter methylation and loss of imprinting.^22, 23^ In contrast, we observed increased expression of PEG3 in TGCTs compared with NT. In concordance with our finding, higher *PEG3* mRNA level was also found in various embryonal cancers (such as rhabdomyosarcoma, medullablastoma and Wilm’s tumors) compared with non-embryonal cancers (acute lymphoblastic leukemia and osteosarcoma) and non-cancerous tissues.^30^

Although its precise function in tumorigenesis remains unclear, PEG3 has been shown to induce p53-mediated apoptosis through Siah1,^31^ Bax translocation from cytosol to mitochondria^24^ and inhibition of Wnt signaling,^32^ suggesting its pro-apoptotic function. In contrast, PEG3 can also activate NF-*κ*B via TRAF2 and protect cells against apoptosis.^25^ Concordant with its anti-apoptotic role, we observed increases in caspase-3 activity and cleaved PARP upon suppression of PEG3 in TGCT cells. Furthermore, we demonstrate that ectopic expression of PEG3 rescues the apoptotic effect caused by miR-514a-3p overexpression. Collectively, our results indicate that miR-514a-3p-mediated regulation of PEG3 has an important role in cell survival of TGCT cells.

To our knowledge, only a single study has reported the role of PEG3 in NF-*κ*B pathway.^25^ Here, we show that silencing of PEG3 expression reduces p105 proteolysis, p50 nuclear translocation and NF-*κ*B reporter activity, which provide additional evidence supporting the involvement of PEG3 in the NF-*κ*B pathway. The NF-*κ*B pathway has important roles in immunity, inflammatory and apoptotic processes.^26^ Activation of the NF-*κ*B pathway is involved in the pathogenesis of many diseases, including some human cancers. However, this has not been reported in TGCT. NF-*κ*B has been suggested to have a role in mammalian spermatogenesis and testicular germ cell apoptosis.^33, 34^ Interestingly, during *in vitro*-induced testicular apoptosis, NF-*κ*B is activated in Sertoli cells and germ cells undergoing apoptosis.^33^ On the other hand, activation of NF-*κ*B in germ cells can induce expression of anti-apoptotic genes that attenuate germ cell apoptosis.^34^ Taken together, we propose that NF-*κ*B activation is required for germ cells survival. Noteworthy, NF-*κ*B activation is involved in the cisplatin resistance of various cancer types;^35, 36^ however, the role of NF-*κ*B activation in cisplatin-resistant of TGCT is yet to be determined.

In conclusion, we provide evidence to support a model of PEG3-mediated activation of NF-*κ*B in TGCT, in which loss of miR-514a-3p in TGCT increases PEG3 expression that recruits TRAF2 and activate the NF-*κ*B pathway for protecting cells from apoptosis.

## Materials and Methods

### Established TGCT cell lines

The TCam-2 seminoma and 2102Ep non-seminoma cell lines were kindly provided by Drs Leendert HJ Looijenga (Erasmus MC-University Medical Center, Rotterdam, Netherlands) and Peter Andrews (University of Sheffield, UK), respectively. TCam-2 cells were grown in RPMI 1640 supplemented with 10% FBS, 2% L-glutamine and 1% penicillin/streptomycin. 2102Ep cells were grown in DMEM supplemented with 10% FBS, 1% L-glutamine and 1% penicillin/streptomycin. Both cell lines were cultured at 37 °C with 5% CO_2_. The authenticity of the cell lines was evaluated by genotyping of short tandem repeats at Bio-Synthesis (Lewisville, TX, USA), and comparison with the genotypes from Palmer *et al.*^17^ All genotypes are detailed in Supplementary Table 5.

### Clinical samples

A total of 15 frozen tumors from 14 TGCT patients (TGCT1–14) and 5 histopathologically verified NT (NT1-5) were provided by the Cooperative Human Tissue Network, which is funded by the National Cancer Institute, USA. Other investigators may have received specimens from the same subjects.

Two tissue microarrays (TMA135 and TMA136) were kindly provided by Dr. John Higgins at Stanford University School of Medicine.^37, 38^ The TMAs included 96 TGCTs, eight non-germ cell testicular tumors and 25 testicular non-tumor tissues as detailed in Supplementary Table 4. The study was approved by the Stanford Human Subjects Review Committee.

### RNA isolation

The mirVana miRNA Isolation Kit (Life Technologies, Carlsbad, CA, USA) was used to extract sRNA for cloning and total RNA for RT-qPCR analysis. AGO2-IP RNA was extracted using TRIzol reagent (Life Technolgies). RNA concentrations were measured using a NanoDrop ND-1000 spectrophotometer (NanoDrop Technologies, Wilmington, DE, USA).

### sRNA cloning

sRNA cloning was performed for nine TGCTs and two NT using previously described methodology.^39^ First, sRNA was ligated with adenylated 3’-adaptor and purified in a 12% denaturing polyacrylamide gel. Second, a ligation was performed with 5’-adaptor, followed by gel purification. Complementary DNA was synthesized with the reverse transcription enzyme SuperScript II (Life Technologies) and the RT primer, followed by PCR amplification for 16–20 cycles using the Amp_F and Amp_R primers. sRNA libraries were sequenced using a Solexa/Illumina sequencing platform (Illumina 1G Genome Analyzer; Illumina Inc., San Diego, CA, USA). The adaptors and primers were according to previous descriptions,^39^ and are detailed in Supplementary Table S6. The sequencing data are available at Gene Expression Omnibus (accession no. GSE59267).

### Sequencing analysis

sRNA-sequencing reads from each of the pooled libraries were separated based on their barcode sequence. All reads were aligned to human mature miRNA sequences (miRBase release 16), using CLC Genomics Workbench 4.5.1 (www.clcbio.com). The mapped reads were counted for each miRNA in individual samples. Remaining reads were further analyzed for identification of novel miRNAs. These reads were initially filtered out from other known structural RNAs, followed by mapping to human genome (hg19) using Bowtie 1.0.0. The aligned reads with less than five genomic locations (to avoid potential repeat sequences) were extended to 100 bases for predicting RNA secondary structures using RNAfold (Vienna RNA Package version 1.8.5; http://rna.tbi.univie.ac.at/).

### TaqMan RT-qPCR

Selected miRNAs and mRNA were quantified using commercially available TaqMan assays (Life Technologies). For mature miRNAs, cDNA was synthesized from 150 ng of total RNA and used to quantitate miR-506 (ID 001050), miR-510 (ID 002241), miR-514a-3p (ID 242955_mat), miR-513c (ID 002756), miR-513b (ID 002757), miR-513a-5p (ID 002090), miR-507 (ID 001051), miR-508-5p (ID 002092), miR-21 (ID 000397), miR-223 (ID 002295), miR-372 (ID 000560) and miR-373 (ID 000561). miR-16 (ID 000391) was used as an endogenous control because of its higher abundance and stability than RNU6B (Supplementary Figure 5).

For the quantification of mRNA, cDNA was synthesized from 500 ng of total RNA using High Capacity cDNA Reverse Transcription Kit (Life Technologies). RT-qPCR reactions were performed for *PEG3* (Hs00300418_s1). Relative expression was normalized against the geometric mean of *GAPDH* (Hs02758991_g1) and *18S* (Hs99999901_s1; Supplementary Figure 5). For quantification of *PEG3* enrichment in AGO2-IP RNA, the geometric mean of miR-372 and miR-373 was used for normalization because of their high abundance in TGCT. Enrichment of *PEG3* mRNA bound to AGO2 was calculated by dividing the relative amount of mRNAs in IP samples to their corresponding input samples. All RT-qPCR reactions were performed in triplicate using Applied Biosystems 7500 Fast Real-time PCR system (Life Technologies).

### DNA extraction, bisulfite conversion and pyrosequencing

DNA methylation density was quantified for five CpGs of the *PEG3* promoter in 12 TGCTs and five NT. DNA was extracted with the QIAamp DNA mini kit (Qiagen GmbH, Hilden, Germany) and bisulfite conversion of genomic DNA was carried out using the EpiTect Bisulfite kit (Qiagen). DNA methylation analysis of bisulfite-converted DNA was performed by pyrosequencing, using previously described procedures.^40^ For each sample, a methylation index (MetI) was calculated as the mean methylation density for the five CpGs analyzed.

### Transfection of miRNA mimics

For miRNA mimics, cells were transfected with three different concentrations (5, 25 and 50 nM) of mirVana miR-510 mimic (MC12923), miR-514a-3p mimic (MC13114) or miRNA mimic Negative Control #1 (NC, ID_4464058) using siPORT NeoFX transfection agent (Life Technologies). Transfection efficiency was assessed by quantification of the expression of the respective miRNAs using RT-qPCR (Supplementary Figure 6).

### WST-1 cell viability assay

WST-1 colorimetric assay (11644807001; Roche Diagnostics, Indianapolis, IN, USA) was performed to determine the effects on cell viability from overexpression of miR-510 or miR-514a-3p. At 48 h post transfection, 20 *μ*l of WST-1 reagent was added to each well. Measurements at the wavelengths 450 nm for the WST-1 cleavage products and 650  nm as reference were recorded after 2 h of incubation using a microplate ELISA reader (VERSAmax; Molecular Devices, Sunnyvale, CA, USA). Results were analyzed with the SoftMax Pro 5 Software (Molecular Devices). All experiments were performed in 10 wells for each condition and repeated at least three times.

### Caspase-3 colorimetric apoptosis assay

Caspase-3 colorimetric assay (K106-100; BioVision, Milpitas, CA, USA) was used to evaluate apoptosis. At 48 h post transfection, 2.5 × 10^6^ transfected cells were resuspended in 50 *μ*l of chilled lysis buffer and incubated on ice for 10 min. Concentration of protein lysate was measured using the BCA protein assay kit (23227; Pierce Biotechnology, Rockford, IL, USA). A total of 150 *μ*g protein lysate was mixed with 50 *μ*l of 2 × Reaction Buffer and 5 *μ*l of 4 mM caspase-3 substrate (DEVD-pNA), and incubated at 37 °C for 2 h. Absorbance at wavelength 405  nm was subsequently determined using microplate ELISA reader (VERSAmax; Molecular Devices) and analyzed with the SoftMax Pro 5 Software (Molecular Devices). Relative caspase-3 activity was calculated after background subtraction and compared with the respective negative control-treated cells. All experiments were conducted three times.

### AGO2-IP

Cells were seeded into five 10-cm tissue culture plates at a density of 2 × 10^6^ cells per plate, and transfected with 50 nM of miR-514a-3p mimic or NC. At 48 h post transfection, cells were lysed for RNA IP using a mouse anti-human AGO2 antibody (ab57113; Abcam, Cambridge, UK) and previously described experimental conditions.^41^

### Construction of plasmids

Two shRNA constructs targeting exon 4 or exon 10 of *PEG3* were generated and cloned into the pcDNA3-U6M2 plasmid using the *Bgl*II and *Kpn*I restriction sites according to previously described methodology.^42^ To generate pCMV6-PEG3-CDS, the full-length coding sequence of human *PEG3* was amplified from a cDNA library of TCam-2 cells and cloned into the pCMV6-Entry with Myc and DDK tags (PS100001, OriGene Technologies, Rockville, MD, USA) at *Bgl*II and *Not*I sites. For constructs with the *PEG3* 3’UTR, synthetic oligonucleotides containing WT or MUT sequences of the putative miR-514a-3p-binding sites of *PEG3* were cloned into pmirGLO Dual-Luciferase miRNA Target Expression Vector (Promega, Madison, WI, USA) at *Pme*I and *Xba*I sites according to the manufacturer’s protocol. The oligonucleotides used for plasmid constructions are given in Supplementary Table 6. All constructs were validated by sequencing at the KIGene core facility of Karolinska Institutet.

### *PEG3* 3´UTR luciferase assay

TCam-2 cells (3 × 10^5^ cells per well) were seeded into a 96-well plate a day before transfection. Cells were co-transfected with 80 ng of pmirGLO reporter plasmid (WT or MUT) and miR-514a-3p mimic or NC using lipofectamine 2000 (Life Technologies). At 48 h post transfection, the Dual-Luciferase Reporter Assay System (E1910; Promega) was applied to detect the firefly and *Renilla* luciferase activities using a microplate luminometer (Centro LB 960, Berthold Technologies, Bad Wildbad, Germany). The firefly luciferase activity was divided by the *Renilla* luciferase activity in each condition and normalized to the *PEG3* 3´UTR (WT) and NC transfectants. All experiments were performed in triplicate independently.

### NF-kappa B luciferase reporter assay

TCam-2 cells (3 × 10^5^ cells per well) were seeded into a 96-well plate a day before transfection. Cells were co-transfected with 80 ng of pGL2 vector (Promega) bearing NF-*κ*B enhancer region and TATA box upstream of firefly luciferase gene,^43^ *Renilla* vector (Promega) and shPEG3-1 vector or miR-514a-3p mimic using lipofectamine 2000 (Life Technologies). After 48 h post transfection, firefly and *Renilla* activity was measured using Dual-Glo Luciferase Assay (E2940; Promega). Firefly luciferase activity was divided by the *Renilla* luciferase activity in each condition and normalized to the shControl or NC transfectants. All experiments were performed in triplicate independently.

### Western blot analysis

For both cell lines and tissues, total protein lysates were prepared using NP-40 lysis buffer (FNN0021; Life Technologies) containing 1 mM of phenylmethanesulfonyl fluoride (Sigma-Aldrich, St. Louis, MO, USA) and protease inhibitor (complete protease inhibitor cocktail; Roche Diagnostics GmbH) and the nuclear protein lysates were prepared using Qproteome Cell Compartment Kit (Qiagen). Protein concentrations were measured using the BCA protein assay kit (Pierce Biotechnology). Samples of 75 *μ*g protein lysate were separated in NuPAGE 10% bis-tris gels (Life Technologies) and transferred to nitrocellulose membranes (LC2001; Life Technologies). After blocking with 5% skim milk powder (Merck, Darmstadt, Germany) diluted in TBS/0.05% Tween 20, membranes were incubated with anti-ZIM2/PEG3 (ab139166; Abcam; at dilution 1:750), anti-TRAF2 (ab12122; Abcam; 1:750), anti-cleaved PARP (ab32064; Abcam; 1:65 000), anti-human AGO2 antibody (ab57113; Abcam; 1:400), anti-H3 (9715; Cell Signaling Technologies, Danvers, MA, USA; 1:10 000) or anti-p105/p50 (ab31412; Abcam; 1:500). Anti-rabbit IgG–HRP (170-6515; Bio-Rad Laboratories, Hercules, CA, USA; 1:3000) or anti-mouse IgG–HRP (sc-2005; Santa Cruz Biotechnology Inc., Dallas, TX, USA; 1:10 000) were used as secondary antibodies. For normalization purposes, membranes were incubated with a GAPDH antibody (sc-47724; Santa Cruz Biotechnology Inc.; 1:10 000). Detection was performed using the Novex ECL HRP chemiluminescent substrate reagent (WP20005; Life Technologies) and Image Reader LAS-1000 (Fujifilm, Tokyo, Japan).

### Co-immunoprecipitation of Myc-PEG3

TCam-2 cells (2 × 10^6^ cells) were seeded into a 10-cm tissue culture plate a day before transfection. Cells were transfected with 3 *μ*g of pCMV6-PEG3-CDS or pCMV6-entry for 48 h and then harvested in modified RIPA lysis buffer without SDS. Total lysates were precleared with Dynabead protein G (10003D; Life Technologies) for 30 min. The lysates were then incubated with 1 *μ*g of anti-MYC antibody (TA150121; OriGene Technologies) for 1 h at 4 °C, followed by 10 *μ*l of Dynabeads and further incubated overnight at 4 °C. The beads were washed three times with lysis buffer and boiled in SDS sample loading buffer (161-0737, Bio-Rad) for 5 min at 90 °C to elute the proteins.

### Immunofluorescence

TCam-2 cells were harvested after 48 h of transfection for cytospin preparation using Shandon Cytospin 3 centrifuge (Thermo Scientific, Waltham, MA, USA). The cytospun cells were washed with cold PBS twice before fixation with 4% paraformaldehyde (USB Corporation/Affymetrix, Santa Clara, CA, USA) for 10 min at room temperature. Cells were permeabilized with cold solution of 0.5% Triton-X for 10 min followed by overnight incubation at 4 °C with anti-PEG3 (ab139166; Abcam; 1:200), anti-p105/p50 (ab31412; Abcam; 1:50) or anti-p100/p52 (3017S; Cell Signaling Technology; 1:100) primary antibodies. Alexa Fluor 488 and 546-conjugated secondary antibodies (A11008 and A11003, respectively; Life Technologies) were diluted to 1:400 and applied on the cells for an hour at room temperature. Cell nuclei were counterstained with DAPI (4′,6-diamidino-2-phenylindole; Sigma-Aldrich) in Vectorshield mounting medium (Vector Laboratories, Burlingame, CA, USA). Images were captured in a confocal microscope Leica TCS SP5 (Lecia Microsystems GmbH, Wetzlar, Germany).

### Immunohistochemistry

Immunohistochemistry was performed according to previously described methodology ^44^, using rabbit polyclonal anti-PEG3 (HPA026070; Sigma-Aldrich) and anti-NF-*κ*B p105/p50 (ab31412; Abcam) antibodies at dilutions 1 :50 and 1:100, respectively. Briefly, TMA slides were de-waxed with xylene and re-hydrated in a descending ethanol series before quenching with 3% hydrogen peroxide (Merck, Darmstadt, Germany) for 20 min. The antigen retrieval was performed with citrate buffer pH 6.0 (S2369; Dako, Glostrup, Denmark) at 95–99 °C for 20 min. Slides were incubated with primary antibodies at 4 °C overnight. The REAL EnVision Detection System Peroxidase/DAB+ (K5007; Dako) was used and slides were counterstained with Mayer’s hematoxylin (Histolab Products AB, Gothenburg, Sweden). The immunostaining was classified as negative or positive (weak, intermediate and strong) based on the scoring of the nuclear staining intensity (0, 1+, 2+ or 3+).

### Statistical analysis

For sRNA-seq data, we performed *χ*^2^-test to determine differentially expressed miRNAs between NT and TGCTs. miRNAs with *P*<0.0001 were considered as significant, and were used to generate heatmap using TreeView (http://jtreeview.sourceforge.net/) and SAM analysis (http://www-stat.stanford.edu/Btibs/SAM/).

For RT-qPCR data, Mann–Whitney *U*-test (Statistica 8.0; StatSoft Inc., Tulsa, OK, USA) was used to compare miRNA expressions between sample groups. Student’s paired *t*-test was performed to analyze transfection experiments using MS Office Excel. All statistical tests were two-sided and *P*<0.05 was considered significant.
