# Supplementary material for: Loss of miR-514a-3p regulation of PEG3 activates the NF-kappa B pathway in human testicular germ cell tumors
Source: Cell Death Dis. 2017 May 4;8(5):e2759–. doi: 10.1038/cddis.2016.464 (PMC5520681; doi:10.1038/cddis.2016.464)
Supplement: Supplementary Information [file cddis2016464x1.pdf]

# Supplementary Information

## Loss of miR-514a-3p regulation of PEG3 activates the NF-kappa B pathway in human testicular germ cell tumors

DM Özata, X Li, L Lee, J Liu, D Warsito, P Hajeri, I Hultman, O Fotouhi, S Marklund, L Ährlund-Richter, CC Juhlin, C Larsson and W-O Lui

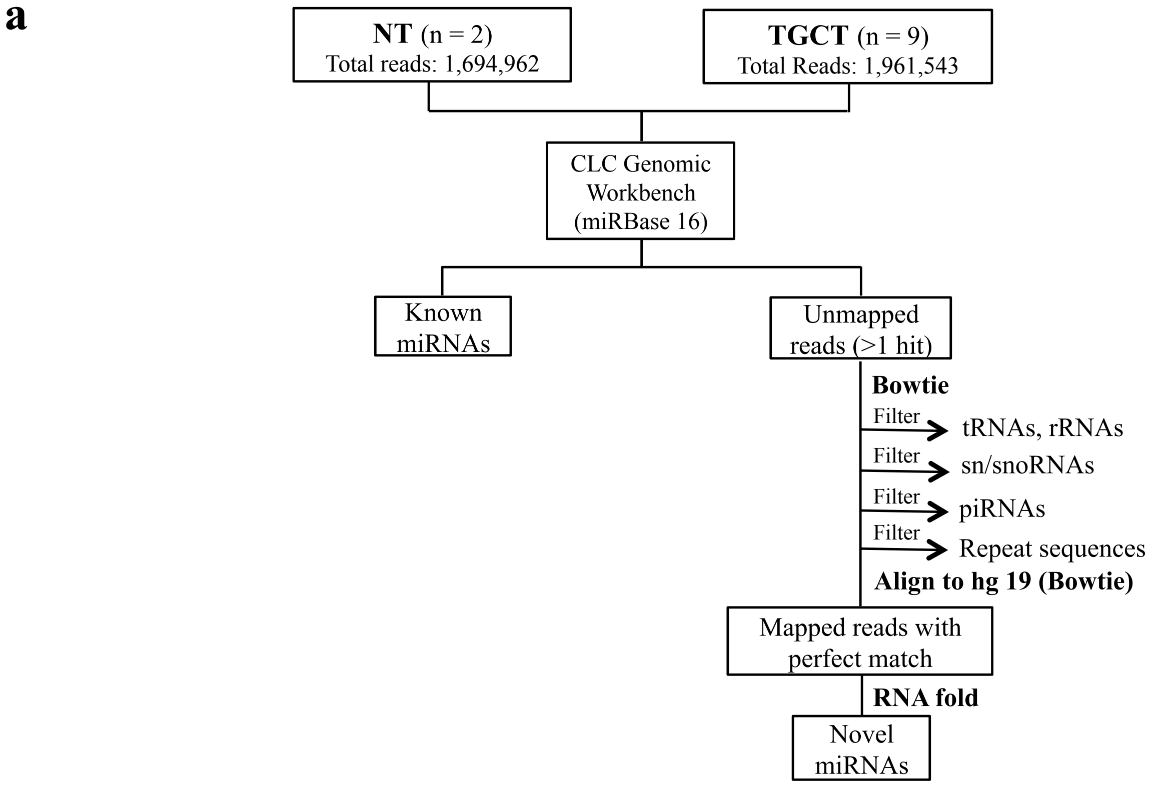


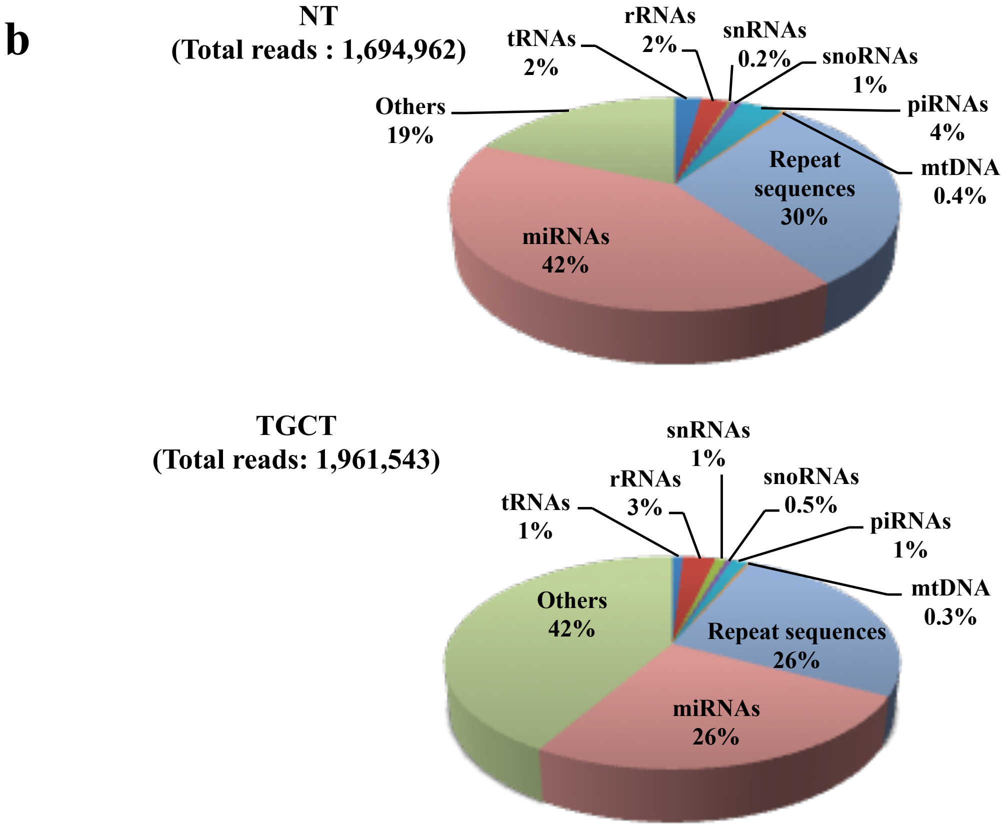


**Supplementary Figure 1.** sRNA sequencing data analysis and sRNA compositions in normal testes (NT) and testicular germ cell tumors (TGCTs). (**a**) Workflow of the sRNA-sequencing data analysis performed in the study. (**b**) Different sRNA compositions of cDNA libraries present in the NT and TGCTs.

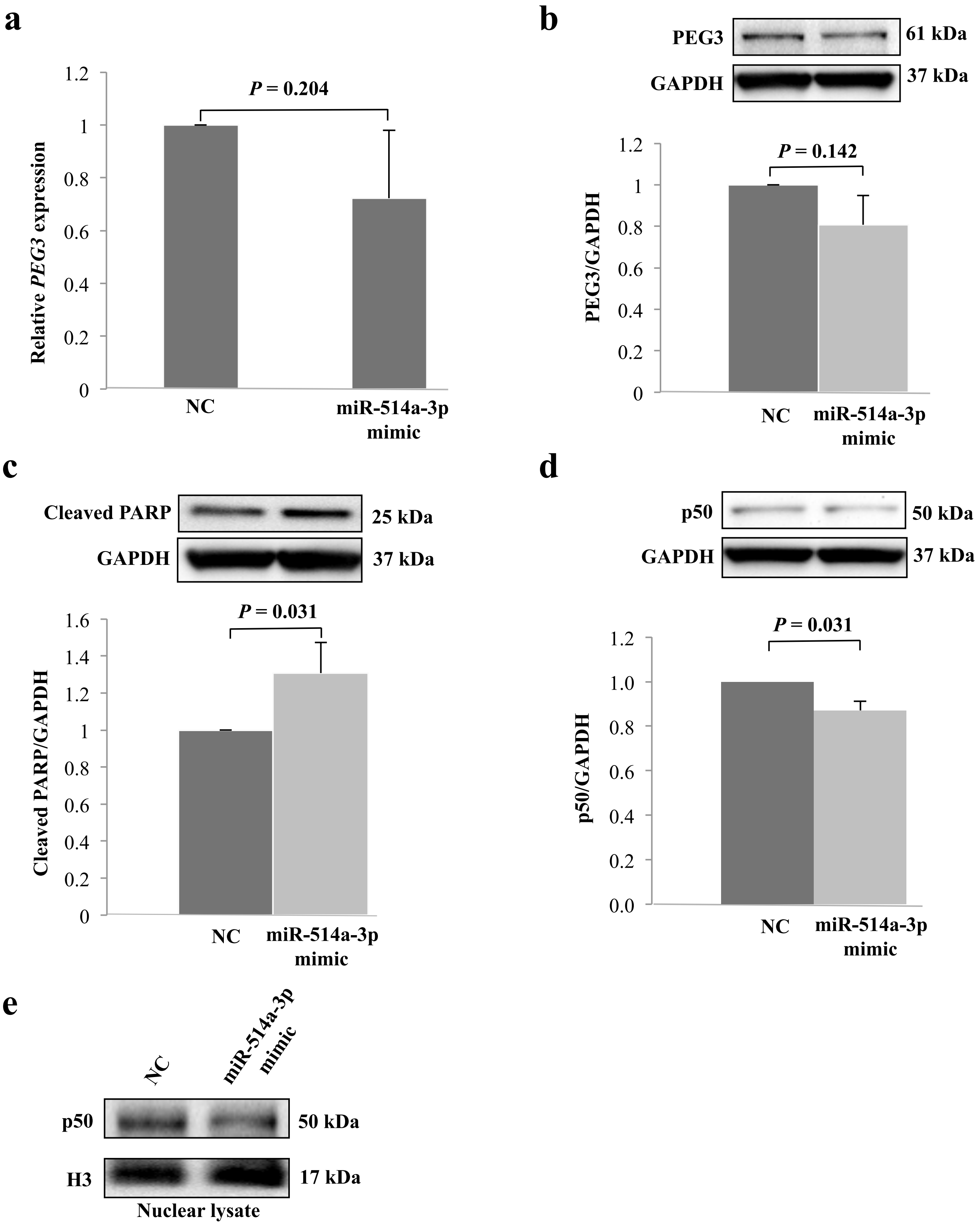


**Supplementary Figure 2.** Evaluation of the functional consequences of miR-514a-3p overexpression in 2102Ep cells. Cells were transfected with miR-514a-3p mimic or negative control (NC) for 48 hours and evaluated for: (**a**) PEG3 mRNA expression by RT-qPCR; (**b**) PEG3 protein by Western blot analysis; (**c**) cleaved PARP by Western blot analysis; and (**d-e**) Western blot analysis of p50 expression from total protein lysate (**d**) and nuclear lysate (**e**). GAPDH and H3 were used as loading controls.

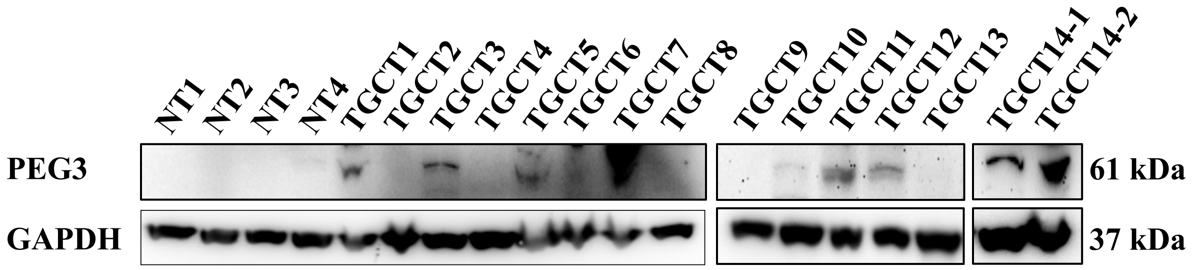


**Supplementary Figure 3.** Measurement of PEG3 protein expression in 4 NT and 15 TGCTs by Western blot analysis. GAPDH was used as a loading control.

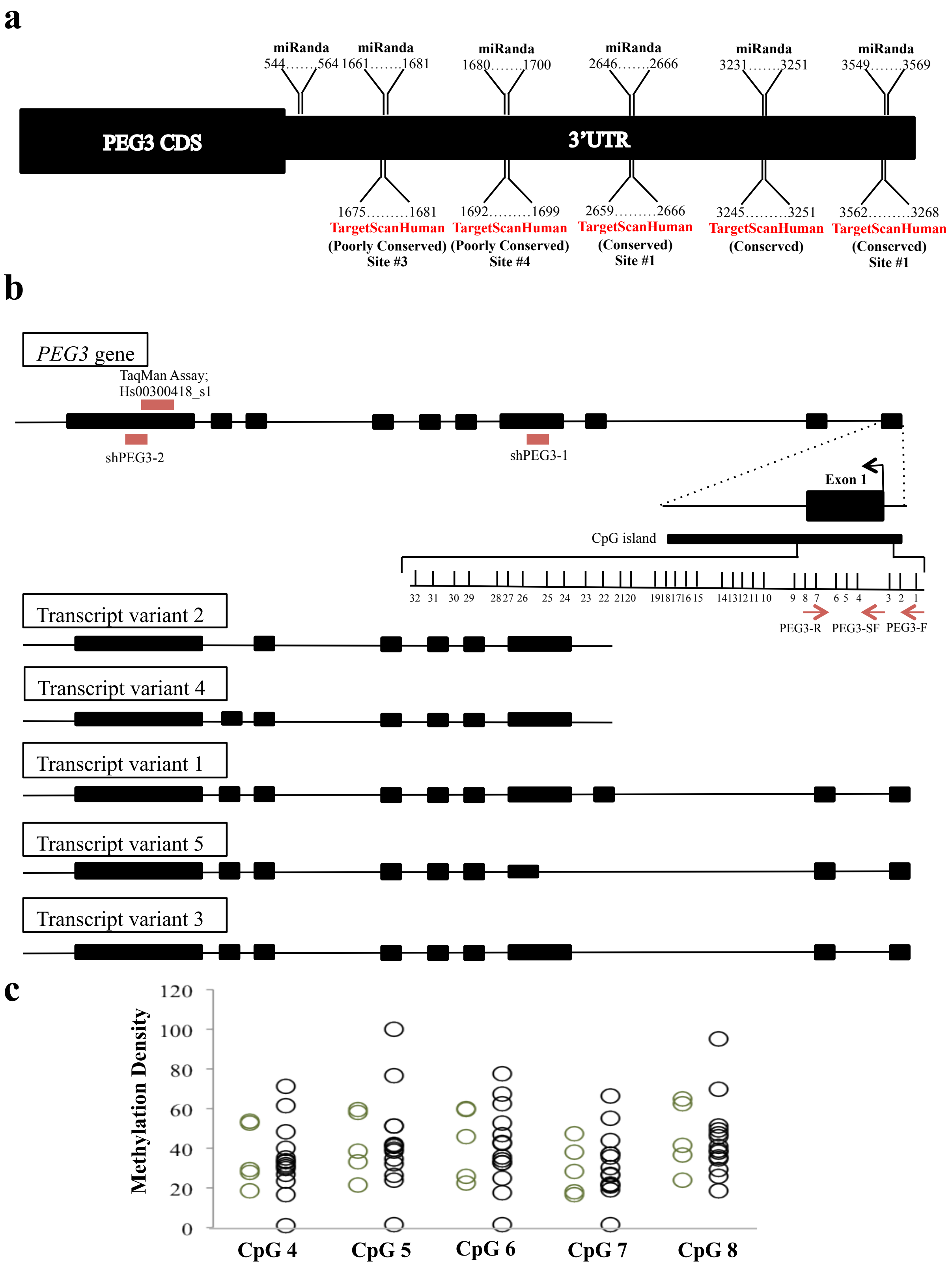


**Supplementary Figure 4.** *PEG3* gene structure, *miR-514a-3p* target sites, shRNA design and methylation analysis. (**a**) Predicted *miR-514a-3p* binding sites in the 3'UTR of *PEG3* by TargetScanHuman and miRanda. Sites #1-4 were included in the *PEG3* 3' UTR reporter assays. (**b**) An illustration of the *PEG3* gene and its transcript variants. The location of the target sequence by the shRNAs and TaqMan gene expression assay is indicated with red boxes, and the Pyrosequencing primers are marked with arrows. (**c**) methylation density (in %) at five CpG sites of the *PEG3* promoter in NT (green empty circles, *n*=5) and TGCTs (black empty circles, *n*=12) determined by Pyrosequencing.

**a**

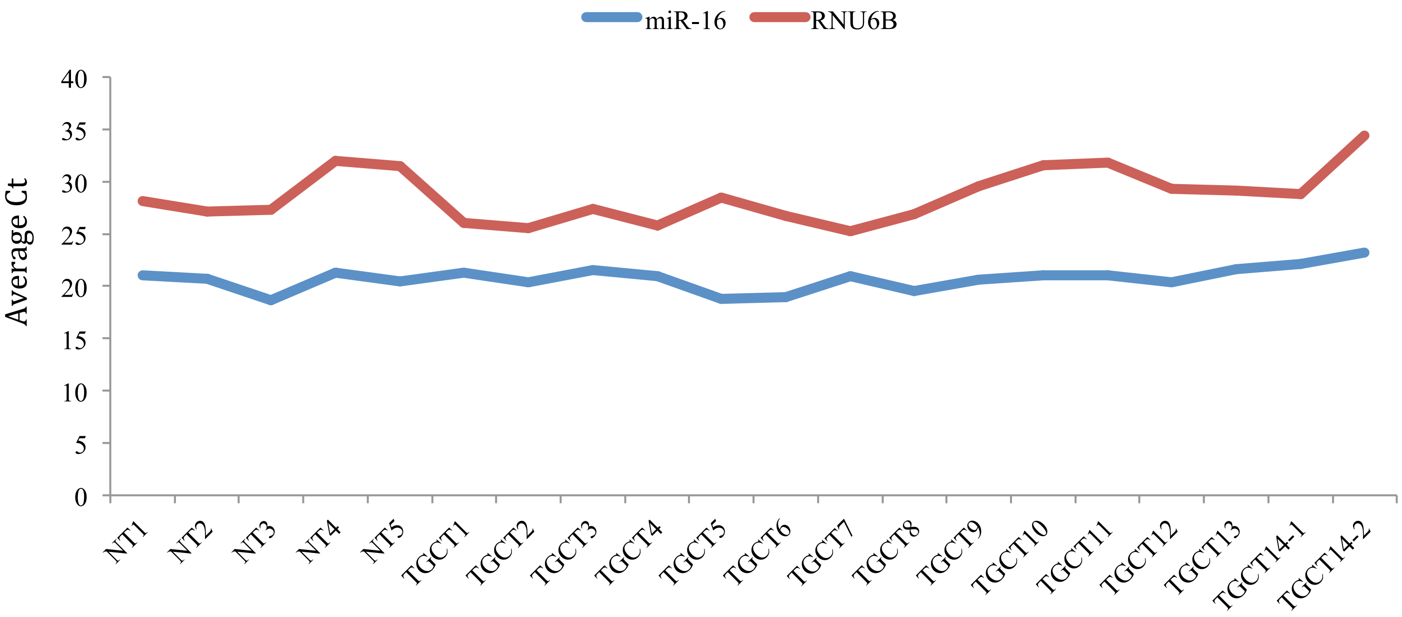


**b**

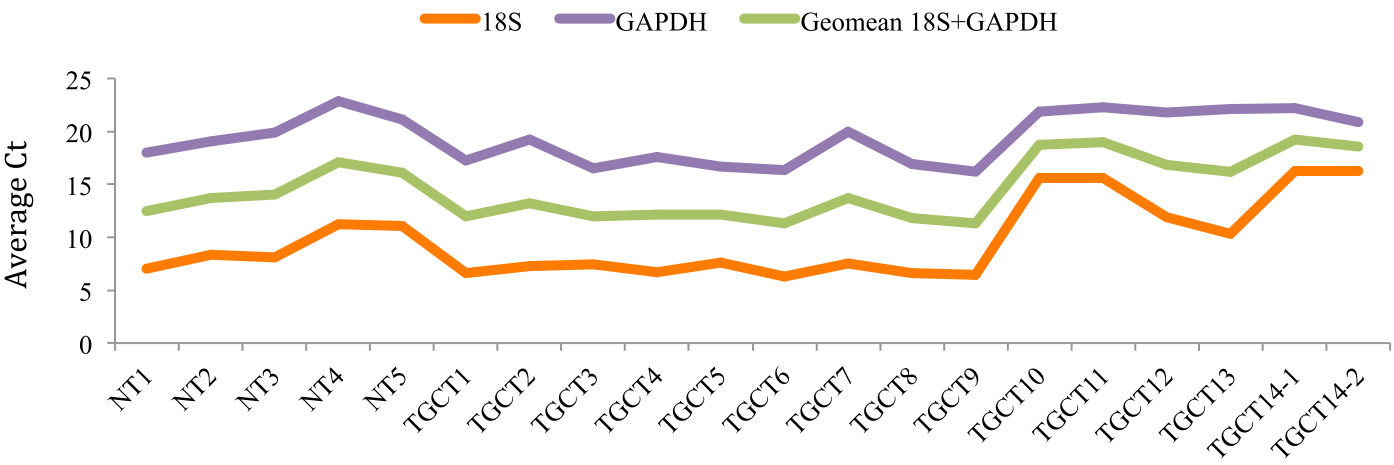


**Supplementary Figure 5.** RT-qPCR analysis of various endogenous controls in 5 NT and 15 TGCTs. (**a**) Comparison of miR-16 and *RNU6B* expression as endogenous control for miRNA quantification. (**b**) Comparison of *18S rRNA*, *GAPDH* and geomean of *18S rRNA* and *GAPDH* as endogenous control for mRNA quantification.

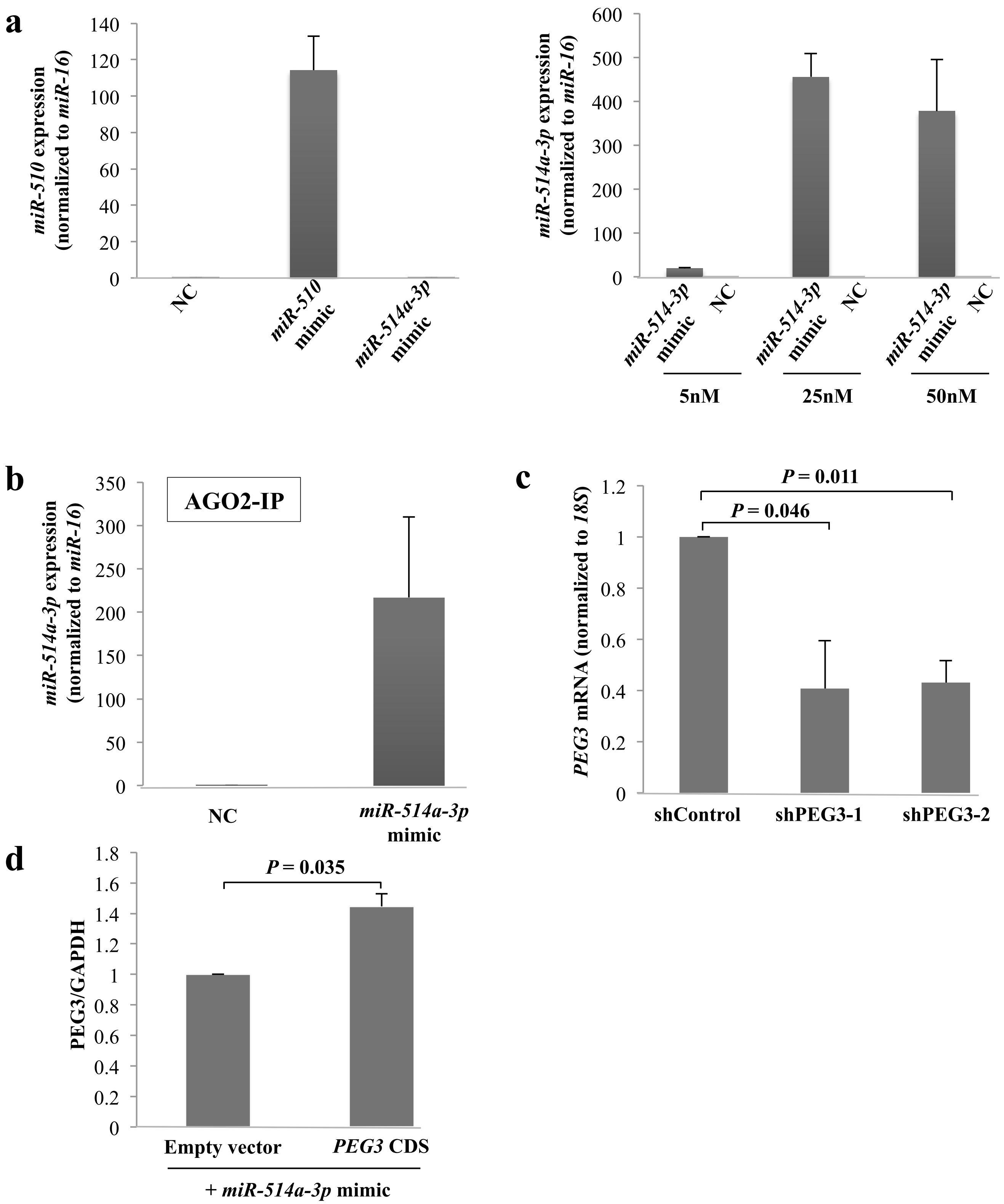


**Supplementary Figure 6.** Evaluation of miRNA, shRNA and PEG3 CDS transfection efficiencies. (**a**) Expressions of miR-510 and miR-514a-3p were quantified by RT-qPCR in cells transfected with miRNA mimic or NC for functional studies. (**b**) miR-514a-3p level was determined in cells transfected with miR-514a-3p mimic or NC for the AGO2 RNA co-immunoprecipitation experiment. (**c**) Evaluation of PEG3 silencing efficiency by RT-qPCR. (**d**) Assessment of PEG3 protein level in cells co-transfected with PEG3 CDS construct or vector control and miR-514a-3p mimic.

**Supplementary Table 1.** Novel candidate miRNAs identified from human TGCTs and NT

| Candidate miRNA | No. of reads | Size (nt) | Stem-loop structures of putative miRNA precursors (5'→ 3') | dG | Genomic location | Gene/ intergenic | intron/ exon/ UTR |
|---|---|---|---|---|---|---|---|
| Candidate_1_5p | 2 | 22 | 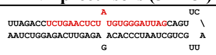 | -57.8 | 4p15.33 | intergenic | |
| Candidate_2_5p | 3 | 19 | 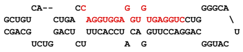 | -44 | 22q13.33 | *PPP6R2* | intron |
| Candidate_3_5p | 3 | 24 | 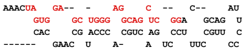 | -26.3 | 4p15.2 | *GPR125* | intron |
| Candidate_4_5p | 4 | 21 | 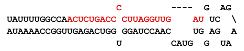 | -49.8 | 15q26.3 | *IGF1R* | intron |
| Candidate_5_5p | 5 | 22 | 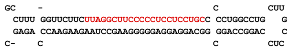 | -91.7 | 1q25.2 | *FAM163A* | intron |
| Candidate_6_5p | 2 | 20 | 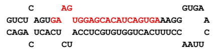 | -51.4 | 9p21.1 | intergenic | |
| Candidate_7_5p | 4 | 21 | 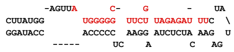 | -35.3 | 1q21.1 | *NBPF9* | intron |
| Candidate_8_5p | 4 | 22 | 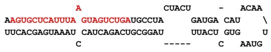 | -47.7 | 2q11.2 | intergenic | |
| Candidate_9_5p | 4 | 20 | 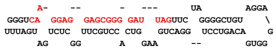 | -35.8 | 5q35.2 | intergenic | |
| Candidate_10_5p | 3 | 25 | 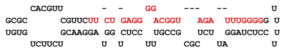 | -38.1 | 7q22.1 | *BUD31* | intron |
| Candidate_11_5p | 4 | 25 | 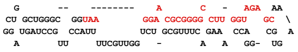 | -34.2 | Yq11.221 | *UTY* | intron |
| Candidate_12_5p | 3 | 21 | 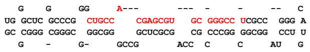 | -61.9 | 14q32.33 | *BC172394* | intron |
| Candidate_13_5p | 2 | 25 | 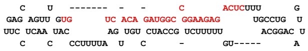 | -30.5 | 20q13.13 | *SLC9A8* | intron |
| Candidate_14_5p | 2 | 22 | 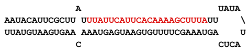 | -46.9 | 8q21.13 | intergenic | |
| Candidate_15_3p | 2 | 20 | 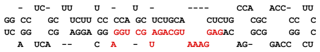 | -34.9 | 3q21.3 | intergenic | |
| Candidate_16_3p | 3 | 22 | 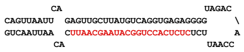 | -55.2 | 5q34 | *TENM2* | intron |
| Candidate_17_3p | 10 | 22 | 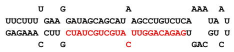 | -45.4 | 17q24.2 | *ABCA6* | intron |
| Candidate_18_3p | 3 | 21 | 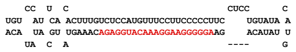 | -61.3 | 15q21.1 | *GALK2* | intron |
| Candidate_19_3p | 2 | 24 | 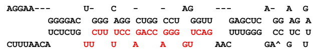 | -34.4 | 9q34.11 | intergenic | |
| Candidate_20_3p | 2 | 25 | 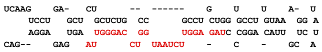 | -32.7 | 3p25.3 | *TATDN2* | intron |
| Candidate_21_3p | 2 | 26 | 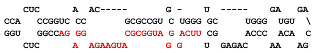 | -40.6 | 16q24.1 | intergenic | |
| Candidate_22_3p | 3 | 22 | 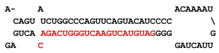 | -54 | Xq28 | *ZNF275* | intron |
| Candidate_23_3p | 2 | 19 | 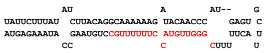 | -47.3 | 5q23.1 | *COMMD10* | intron |
| Candidate_24_3p | 2 | 23 | 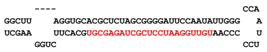 | -68.3 | 1p32.3 | *TCEANC2* | intron |
| Candidate_25_3p | 2 | 20 | 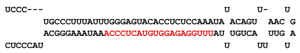 | 69.4 | 6q25.1 | intergenic | |
| Candidate_26_3p | 3 | 21 | 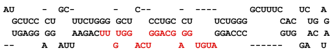 | -35.9 | 21q22.11 | intergenic | |
| Candidate_27_3p | 4 | 22 | 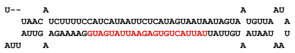 | -51.2 | 1p31.3 | *INADL* | intron |
| Candidate_28_3p | 5 | 22 | 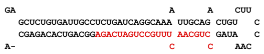 | -69.4 | 2q12.1 | *IL18RAP* | intron |
| Candidate_29_3p | 3 | 22 | 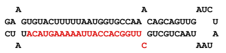 | -48.8 | 1p21.3 | intergenic | |

dG = free energies of predicted secondary structures; UTR = Untranslated region

**Supplementary Table 2.** Statistical analysis of miRNA variations between TGCTs and NT

| miRNA | Observation (no. of reads) | | | | Expectation (no. of reads) | | | | *P*-value # |
|---|---|---|---|---|---|---|---|---|---|
| | NT | | TGCTs | | NT | | TGCT | | |
| | Present | Absent | Present | Absent | Present | Absent | Present | Absent | |
| **Total reads** | 702465 | | 501743 | | | | | | |
| **Differentially expressed miRNAs between TGCTs and NT (P<0.0001)** | | | | | | | | | |
| let-7a | 68831 | 633634 | 32629 | 469114 | 59186 | 643279 | 42274 | 459469 | < 0.0001 |
| let-7b | 120293 | 582172 | 20486 | 481257 | 82122 | 620343 | 58657 | 443086 | < 0.0001 |
| let-7c | 56852 | 645613 | 11006 | 490737 | 39584 | 662881 | 28274 | 473469 | < 0.0001 |
| miR-127-3p | 9454 | 693011 | 1843 | 499900 | 6590 | 695875 | 4707 | 497036 | < 0.0001 |
| miR-1323 | 141 | 702324 | 1742 | 500001 | 1098 | 701367 | 785 | 500958 | < 0.0001 |
| miR-142-3p | 1014 | 701451 | 7070 | 494673 | 4716 | 697749 | 3368 | 498375 | < 0.0001 |
| miR-142-5p | 485 | 701980 | 2065 | 499678 | 1488 | 700977 | 1062 | 500681 | < 0.0001 |
| miR-143 | 15020 | 687445 | 47799 | 453944 | 36645 | 665820 | 26174 | 475569 | < 0.0001 |
| miR-145 | 2839 | 699626 | 12428 | 489315 | 8906 | 693559 | 6361 | 495382 | < 0.0001 |
| miR-150 | 267 | 702198 | 2686 | 499057 | 1723 | 700742 | 1230 | 500513 | < 0.0001 |
| miR-202 | 13081 | 689384 | 124 | 501619 | 7703 | 694762 | 5502 | 496241 | < 0.0001 |
| miR-202* | 31326 | 671139 | 3004 | 498739 | 20026 | 682439 | 14304 | 487439 | < 0.0001 |
| miR-21 | 17129 | 685336 | 68920 | 432823 | 50196 | 652269 | 35853 | 465890 | < 0.0001 |
| miR-223 | 281 | 702184 | 1855 | 499888 | 1246 | 701219 | 890 | 500853 | < 0.0001 |
| miR-23a | 7866 | 694599 | 11600 | 490143 | 11355 | 691110 | 8111 | 493632 | < 0.0001 |
| miR-27a | 881 | 701584 | 2522 | 499221 | 1985 | 700480 | 1418 | 500325 | < 0.0001 |
| miR-27b | 1558 | 700907 | 3346 | 498397 | 2861 | 699604 | 2043 | 499700 | < 0.0001 |
| miR-302a | 455 | 702010 | 1942 | 499801 | 1398 | 701067 | 999 | 500744 | < 0.0001 |
| miR-302b | 303 | 702162 | 2755 | 498988 | 1784 | 700681 | 1274 | 500469 | < 0.0001 |
| miR-320a | 17242 | 685223 | 2009 | 499734 | 11230 | 691235 | 8021 | 493722 | < 0.0001 |
| miR-335 | 6939 | 695526 | 24037 | 477706 | 18070 | 684395 | 12906 | 488837 | < 0.0001 |
| miR-374a | 452 | 702013 | 2087 | 499656 | 1481 | 700984 | 1058 | 500685 | < 0.0001 |

| | | | | | | | | | |
|---|---|---|---|---|---|---|---|---|---|
| miR-424 | 10466 | 691999 | 1232 | 500511 | 6824 | 695641 | 4874 | 496869 | < 0.0001 |
| miR-451 | 587 | 701878 | 3009 | 498734 | 2098 | 700367 | 1498 | 500245 | < 0.0001 |
| miR-507 | 2470 | 699995 | 19 | 501724 | 1452 | 701013 | 1037 | 500706 | < 0.0001 |
| miR-508-3p | 3188 | 699277 | 14 | 501729 | 1868 | 700597 | 1334 | 500409 | < 0.0001 |
| miR-510 | 12839 | 689626 | 128 | 501615 | 7564 | 694901 | 5403 | 496340 | < 0.0001 |
| miR-513b | 22959 | 679506 | 64 | 501679 | 13430 | 689035 | 9593 | 492150 | < 0.0001 |
| miR-513c | 3465 | 699000 | 32 | 501711 | 2040 | 700425 | 1457 | 500286 | < 0.0001 |
| miR-514 | 5104 | 697361 | 34 | 501709 | 2997 | 699468 | 2141 | 499602 | < 0.0001 |
| miR-514b-5p | 5695 | 696770 | 65 | 501678 | 3360 | 699105 | 2400 | 499343 | < 0.0001 |
| miR-7 | 1129 | 701336 | 2860 | 498883 | 2327 | 700138 | 1662 | 500081 | < 0.0001 |
| miR-15b | 4180 | 698285 | 6176 | 495567 | 6041 | 696424 | 4315 | 497428 | < 0.0001 |
| miR-182 | 689 | 701776 | 2010 | 499733 | 1574 | 700891 | 1125 | 500618 | < 0.0001 |
| miR-515-5p | 69 | 702396 | 944 | 500799 | 591 | 701874 | 422 | 501321 | < 0.0001 |
| miR-144 | 92 | 702373 | 978 | 500765 | 624 | 701841 | 446 | 501297 | < 0.0001 |
| miR-373 | 606 | 701859 | 1782 | 499961 | 1393 | 701072 | 995 | 500748 | < 0.0001 |
| miR-15a | 674 | 701791 | 1862 | 499881 | 1479 | 700986 | 1057 | 500686 | < 0.0001 |
| miR-367 | 102 | 702363 | 941 | 500802 | 608 | 701857 | 435 | 501308 | < 0.0001 |
| miR-509-3p | 1392 | 701073 | 11 | 501732 | 818 | 701647 | 585 | 501158 | < 0.0001 |
| miR-19b | 808 | 701657 | 1916 | 499827 | 1589 | 700876 | 1135 | 500608 | < 0.0001 |
| miR-424* | 1890 | 700575 | 203 | 501540 | 1221 | 701244 | 872 | 500871 | < 0.0001 |
| miR-517a | 76 | 702389 | 794 | 500949 | 508 | 701957 | 362 | 501381 | < 0.0001 |
| miR-423-5p | 2396 | 700069 | 404 | 501339 | 1633 | 700832 | 1167 | 500576 | < 0.0001 |
| miR-1283 | 85 | 702380 | 764 | 500979 | 495 | 701970 | 354 | 501389 | < 0.0001 |
| miR-23b | 3391 | 699074 | 4569 | 497174 | 4643 | 697822 | 3317 | 498426 | < 0.0001 |
| miR-136 | 2654 | 699811 | 547 | 501196 | 1867 | 700598 | 1334 | 500409 | < 0.0001 |
| miR-1 | 193 | 702272 | 938 | 500805 | 660 | 701805 | 471 | 501272 | < 0.0001 |
| miR-508-5p | 1051 | 701414 | 10 | 501733 | 619 | 701846 | 442 | 501301 | < 0.0001 |
| miR-484 | 198 | 702267 | 890 | 500853 | 635 | 701830 | 453 | 501290 | < 0.0001 |
| miR-524-5p | 43 | 702422 | 590 | 501153 | 369 | 702096 | 264 | 501479 | < 0.0001 |
| miR-503 | 1161 | 701304 | 63 | 501680 | 714 | 701751 | 510 | 501233 | < 0.0001 |

| | | | | | | | | | |
|---|---|---|---|---|---|---|---|---|---|
| miR-26a | 24474 | 677991 | 22114 | 479629 | 27177 | 675288 | 19411 | 482332 | < 0.0001 |
| miR-518b | 88 | 702377 | 655 | 501088 | 433 | 702032 | 310 | 501433 | < 0.0001 |
| miR-302d | 374 | 702091 | 1091 | 500652 | 855 | 701610 | 610 | 501133 | < 0.0001 |
| miR-486-5p | 256 | 702209 | 896 | 500847 | 672 | 701793 | 480 | 501263 | < 0.0001 |
| miR-519a | 30 | 702435 | 509 | 501234 | 314 | 702151 | 225 | 501518 | < 0.0001 |
| miR-16 | 1309 | 701156 | 2171 | 499572 | 2030 | 700435 | 1450 | 500293 | < 0.0001 |
| miR-517c | 19 | 702446 | 476 | 501267 | 289 | 702176 | 206 | 501537 | < 0.0001 |
| miR-526b | 25 | 702440 | 485 | 501258 | 298 | 702167 | 212 | 501531 | < 0.0001 |
| miR-302a* | 410 | 702055 | 1082 | 500661 | 870 | 701595 | 622 | 501121 | < 0.0001 |
| miR-125b | 17132 | 685333 | 15857 | 485886 | 19244 | 683221 | 13745 | 487998 | < 0.0001 |
| miR-92a | 1235 | 701230 | 2032 | 499711 | 1906 | 700559 | 1361 | 500382 | < 0.0001 |
| miR-520a-5p | 27 | 702438 | 455 | 501288 | 281 | 702184 | 201 | 501542 | < 0.0001 |
| miR-302b* | 645 | 701820 | 1333 | 500410 | 1154 | 701311 | 824 | 500919 | < 0.0001 |
| miR-193a-3p | 123 | 702342 | 606 | 501137 | 425 | 702040 | 304 | 501439 | < 0.0001 |
| miR-107 | 878 | 701587 | 1568 | 500175 | 1427 | 701038 | 1019 | 500724 | < 0.0001 |
| miR-519c-3p | 23 | 702442 | 396 | 501347 | 244 | 702221 | 175 | 501568 | < 0.0001 |
| miR-200c | 173 | 702292 | 653 | 501090 | 482 | 701983 | 344 | 501399 | < 0.0001 |
| miR-96 | 154 | 702311 | 617 | 501126 | 450 | 702015 | 321 | 501422 | < 0.0001 |
| miR-512-3p | 32 | 702433 | 402 | 501341 | 253 | 702212 | 181 | 501562 | < 0.0001 |
| miR-134 | 756 | 701709 | 31 | 501712 | 459 | 702006 | 328 | 501415 | < 0.0001 |
| miR-25 | 1120 | 701345 | 1739 | 500004 | 1668 | 700797 | 1191 | 500552 | < 0.0001 |
| miR-205 | 10 | 702455 | 326 | 501417 | 196 | 702269 | 140 | 501603 | < 0.0001 |
| miR-542-5p | 748 | 701717 | 50 | 501693 | 466 | 701999 | 332 | 501411 | < 0.0001 |
| miR-19a | 203 | 702262 | 636 | 501107 | 489 | 701976 | 350 | 501393 | < 0.0001 |
| miR-519b-3p | 27 | 702438 | 346 | 501397 | 218 | 702247 | 155 | 501588 | < 0.0001 |
| miR-99a | 3067 | 699398 | 1110 | 500633 | 2437 | 700028 | 1740 | 500003 | < 0.0001 |
| miR-155 | 474 | 701991 | 951 | 500792 | 831 | 701634 | 594 | 501149 | < 0.0001 |
| miR-520c-3p | 23 | 702442 | 312 | 501431 | 195 | 702270 | 140 | 501603 | < 0.0001 |
| miR-218 | 112 | 702353 | 469 | 501274 | 339 | 702126 | 242 | 501501 | < 0.0001 |
| miR-25* | 839 | 701626 | 114 | 501629 | 556 | 701909 | 397 | 501346 | < 0.0001 |

| | | | | | | | | | |
|---|---|---|---|---|---|---|---|---|---|
| miR-498 | 42 | 702423 | 325 | 501418 | 214 | 702251 | 153 | 501590 | < 0.0001 |
| miR-30b | 1297 | 701168 | 1779 | 499964 | 1794 | 700671 | 1282 | 500461 | < 0.0001 |
| miR-141 | 10 | 702455 | 258 | 501485 | 156 | 702309 | 112 | 501631 | < 0.0001 |
| miR-494 | 701 | 701764 | 77 | 501666 | 454 | 702011 | 324 | 501419 | < 0.0001 |
| miR-302c | 22 | 702443 | 275 | 501468 | 173 | 702292 | 124 | 501619 | < 0.0001 |
| miR-516a-5p | 21 | 702444 | 269 | 501474 | 169 | 702296 | 121 | 501622 | < 0.0001 |
| miR-26b | 5000 | 697465 | 5047 | 496696 | 5861 | 696604 | 4186 | 497557 | < 0.0001 |
| miR-365 | 434 | 702031 | 832 | 500911 | 739 | 701726 | 527 | 501216 | < 0.0001 |
| miR-30e | 584 | 701881 | 993 | 500750 | 920 | 701545 | 657 | 501086 | < 0.0001 |
| miR-183 | 60 | 702405 | 326 | 501417 | 225 | 702240 | 161 | 501582 | < 0.0001 |
| miR-574-5p | 1371 | 701094 | 386 | 501357 | 1025 | 701440 | 732 | 501011 | < 0.0001 |
| miR-374b | 505 | 701960 | 885 | 500858 | 811 | 701654 | 579 | 501164 | < 0.0001 |
| miR-9* | 19 | 702446 | 237 | 501506 | 149 | 702316 | 107 | 501636 | < 0.0001 |
| miR-146a | 360 | 702105 | 712 | 501031 | 625 | 701840 | 447 | 501296 | < 0.0001 |
| miR-214 | 2060 | 700405 | 741 | 501002 | 1634 | 700831 | 1167 | 500576 | < 0.0001 |
| miR-335* | 17 | 702448 | 224 | 501519 | 141 | 702324 | 100 | 501643 | < 0.0001 |
| miR-92a-1* | 959 | 701506 | 220 | 501523 | 688 | 701777 | 491 | 501252 | < 0.0001 |
| miR-342-3p | 182 | 702283 | 476 | 501267 | 384 | 702081 | 274 | 501469 | < 0.0001 |
| miR-506 | 381 | 702084 | 7 | 501736 | 226 | 702239 | 162 | 501581 | < 0.0001 |
| miR-376c | 4493 | 697972 | 2119 | 499624 | 3857 | 698608 | 2755 | 498988 | < 0.0001 |
| miR-518e | 15 | 702450 | 212 | 501531 | 132 | 702333 | 95 | 501648 | < 0.0001 |
| miR-520d-3p | 20 | 702445 | 220 | 501523 | 140 | 702325 | 100 | 501643 | < 0.0001 |
| miR-30c | 1333 | 701132 | 1671 | 500072 | 1752 | 700713 | 1252 | 500491 | < 0.0001 |
| miR-361-3p | 46 | 702419 | 259 | 501484 | 178 | 702287 | 127 | 501616 | < 0.0001 |
| miR-10b* | 427 | 702038 | 29 | 501714 | 266 | 702199 | 190 | 501553 | < 0.0001 |
| miR-28-5p | 236 | 702229 | 521 | 501222 | 442 | 702023 | 315 | 501428 | < 0.0001 |
| miR-518a-3p | 12 | 702453 | 190 | 501553 | 118 | 702347 | 84 | 501659 | < 0.0001 |
| hsa-let-7e | 11516 | 690949 | 6529 | 495214 | 10526 | 691939 | 7519 | 494224 | < 0.0001 |
| miR-432 | 1324 | 701141 | 423 | 501320 | 1019 | 701446 | 728 | 501015 | < 0.0001 |
| hsa-let-7g | 5204 | 697261 | 4937 | 496806 | 5916 | 696549 | 4225 | 497518 | < 0.0001 |

| | | | | | | | | | |
|---|---|---|---|---|---|---|---|---|---|
| miR-22 | 966 | 701499 | 1257 | 500486 | 1297 | 701168 | 926 | 500817 | < 0.0001 |
| miR-106b | 125 | 702340 | 350 | 501393 | 277 | 702188 | 198 | 501545 | < 0.0001 |
| miR-151-5p | 6511 | 695954 | 5976 | 495767 | 7284 | 695181 | 5203 | 496540 | < 0.0001 |
| miR-221 | 1000 | 701465 | 289 | 501454 | 752 | 701713 | 537 | 501206 | < 0.0001 |
| miR-199a-5p | 2843 | 699622 | 1272 | 500471 | 2400 | 700065 | 1715 | 500028 | < 0.0001 |
| miR-372 | 446 | 702019 | 723 | 501020 | 682 | 701783 | 487 | 501256 | < 0.0001 |
| miR-518c | 13 | 702452 | 166 | 501577 | 104 | 702361 | 75 | 501668 | < 0.0001 |
| miR-518f* | 10 | 702455 | 158 | 501585 | 98 | 702367 | 70 | 501673 | < 0.0001 |
| miR-515-3p | 27 | 702438 | 186 | 501557 | 124 | 702341 | 89 | 501654 | < 0.0001 |
| miR-516b | 61 | 702404 | 237 | 501506 | 174 | 702291 | 124 | 501619 | < 0.0001 |
| miR-135b | 250 | 702215 | 481 | 501262 | 426 | 702039 | 305 | 501438 | < 0.0001 |
| miR-140-3p | 1023 | 701442 | 324 | 501419 | 786 | 701679 | 561 | 501182 | < 0.0001 |
| miR-509-3-5p | 255 | 702210 | 4 | 501739 | 151 | 702314 | 108 | 501635 | < 0.0001 |
| miR-145* | 240 | 702225 | 463 | 501280 | 410 | 702055 | 293 | 501450 | < 0.0001 |
| miR-339-3p | 87 | 702378 | 266 | 501477 | 206 | 702259 | 147 | 501596 | < 0.0001 |
| miR-21* | 54 | 702411 | 217 | 501526 | 158 | 702307 | 113 | 501630 | < 0.0001 |
| miR-483-5p | 390 | 702075 | 56 | 501687 | 260 | 702205 | 186 | 501557 | < 0.0001 |
| miR-518f | 15 | 702450 | 141 | 501602 | 91 | 702374 | 65 | 501678 | < 0.0001 |
| miR-519d | 34 | 702431 | 175 | 501568 | 122 | 702343 | 87 | 501656 | < 0.0001 |
| miR-10a | 94 | 702371 | 262 | 501481 | 208 | 702257 | 148 | 501595 | < 0.0001 |
| miR-148a | 58 | 702407 | 208 | 501535 | 155 | 702310 | 111 | 501632 | < 0.0001 |
| miR-513a-3p | 219 | 702246 | 4 | 501739 | 130 | 702335 | 93 | 501650 | < 0.0001 |
| miR-371-5p | 1120 | 701345 | 403 | 501340 | 888 | 701577 | 635 | 501108 | < 0.0001 |
| miR-337-3p | 387 | 702078 | 593 | 501150 | 572 | 701893 | 408 | 501335 | < 0.0001 |
| miR-299-3p | 408 | 702057 | 73 | 501670 | 281 | 702184 | 200 | 501543 | < 0.0001 |
| miR-31 | 29 | 702436 | 155 | 501588 | 107 | 702358 | 77 | 501666 | < 0.0001 |
| miR-525-5p | 19 | 702446 | 136 | 501607 | 90 | 702375 | 65 | 501678 | < 0.0001 |
| miR-127-5p | 322 | 702143 | 48 | 501695 | 216 | 702249 | 154 | 501589 | < 0.0001 |
| miR-197 | 62 | 702403 | 196 | 501547 | 151 | 702314 | 107 | 501636 | < 0.0001 |
| miR-517* | 14 | 702451 | 118 | 501625 | 77 | 702388 | 55 | 501688 | < 0.0001 |

| | | | | | | | | | |
|---|---|---|---|---|---|---|---|---|---|
| miR-340 | 155 | 702310 | 309 | 501434 | 271 | 702194 | 193 | 501550 | < 0.0001 |
| miR-186 | 1499 | 700966 | 645 | 501098 | 1251 | 701214 | 893 | 500850 | < 0.0001 |
| miR-512-5p | 27 | 702438 | 137 | 501606 | 96 | 702369 | 68 | 501675 | < 0.0001 |
| miR-409-3p | 357 | 702108 | 66 | 501677 | 247 | 702218 | 176 | 501567 | < 0.0001 |
| miR-371-3p | 109 | 702356 | 246 | 501497 | 207 | 702258 | 148 | 501595 | < 0.0001 |
| miR-128 | 136 | 702329 | 277 | 501466 | 241 | 702224 | 172 | 501571 | < 0.0001 |
| miR-192 | 478 | 701987 | 635 | 501108 | 649 | 701816 | 464 | 501279 | < 0.0001 |
| miR-200b | 9 | 702456 | 97 | 501646 | 62 | 702403 | 44 | 501699 | < 0.0001 |
| miR-9 | 78 | 702387 | 202 | 501541 | 163 | 702302 | 117 | 501626 | < 0.0001 |
| miR-23a* | 164 | 702301 | 4 | 501739 | 98 | 702367 | 70 | 501673 | < 0.0001 |
| miR-664* | 214 | 702251 | 20 | 501723 | 137 | 702328 | 97 | 501646 | < 0.0001 |
| miR-32 | 117 | 702348 | 247 | 501496 | 212 | 702253 | 152 | 501591 | < 0.0001 |
| miR-18a | 78 | 702387 | 197 | 501546 | 160 | 702305 | 115 | 501628 | < 0.0001 |
| miR-126* | 5166 | 697299 | 4513 | 497230 | 5646 | 696819 | 4033 | 497710 | < 0.0001 |
| miR-30d | 1245 | 701220 | 1313 | 500430 | 1492 | 700973 | 1066 | 500677 | < 0.0001 |
| miR-324-5p | 416 | 702049 | 106 | 501637 | 305 | 702160 | 217 | 501526 | < 0.0001 |
| miR-148b | 51 | 702414 | 148 | 501595 | 116 | 702349 | 83 | 501660 | < 0.0001 |
| miR-450a | 1274 | 701191 | 573 | 501170 | 1077 | 701388 | 770 | 500973 | < 0.0001 |
| miR-590-3p | 49 | 702416 | 144 | 501599 | 113 | 702352 | 80 | 501663 | < 0.0001 |
| hsa-let-7d | 4694 | 697771 | 2689 | 499054 | 4307 | 698158 | 3076 | 498667 | < 0.0001 |
| miR-34a | 481 | 701984 | 151 | 501592 | 369 | 702096 | 263 | 501480 | < 0.0001 |
| miR-497 | 748 | 701717 | 289 | 501454 | 605 | 701860 | 432 | 501311 | < 0.0001 |
| miR-141* | 5 | 702460 | 69 | 501674 | 43 | 702422 | 31 | 501712 | < 0.0001 |
| miR-526b* | 5 | 702460 | 68 | 501675 | 43 | 702422 | 30 | 501713 | < 0.0001 |
| miR-10b | 1491 | 700974 | 1472 | 500271 | 1728 | 700737 | 1235 | 500508 | < 0.0001 |
| miR-3613-5p | 48 | 702417 | 135 | 501608 | 107 | 702358 | 76 | 501667 | < 0.0001 |
| miR-101 | 1129 | 701336 | 1155 | 500588 | 1332 | 701133 | 952 | 500791 | < 0.0001 |
| miR-130b | 194 | 702271 | 300 | 501443 | 288 | 702177 | 206 | 501537 | < 0.0001 |
| miR-660 | 1027 | 701438 | 455 | 501288 | 865 | 701600 | 617 | 501126 | < 0.0001 |
| miR-29b-2* | 178 | 702287 | 25 | 501718 | 118 | 702347 | 85 | 501658 | < 0.0001 |

| | | | | | | | | | |
|---|---|---|---|---|---|---|---|---|---|
| miR-193b* | 184 | 702281 | 28 | 501715 | 124 | 702341 | 88 | 501655 | < 0.0001 |
| miR-345 | 29 | 702436 | 101 | 501642 | 76 | 702389 | 54 | 501689 | < 0.0001 |
| miR-378* | 14 | 702451 | 77 | 501666 | 53 | 702412 | 38 | 501705 | < 0.0001 |
| miR-495 | 429 | 702036 | 522 | 501221 | 555 | 701910 | 396 | 501347 | < 0.0001 |
| miR-27b* | 105 | 702360 | 196 | 501547 | 176 | 702289 | 125 | 501618 | < 0.0001 |
| miR-30a | 463 | 702002 | 552 | 501191 | 592 | 701873 | 423 | 501320 | < 0.0001 |
| miR-181c | 49 | 702416 | 127 | 501616 | 103 | 702362 | 73 | 501670 | < 0.0001 |
| miR-146b-5p | 176 | 702289 | 271 | 501472 | 261 | 702204 | 186 | 501557 | < 0.0001 |
| miR-520a-3p | 4 | 702461 | 56 | 501687 | 35 | 702430 | 25 | 501718 | < 0.0001 |
| miR-23c | 18 | 702447 | 81 | 501662 | 58 | 702407 | 41 | 501702 | < 0.0001 |
| miR-411 | 339 | 702126 | 99 | 501644 | 256 | 702209 | 182 | 501561 | < 0.0001 |
| miR-126 | 4895 | 697570 | 4142 | 497601 | 5272 | 697193 | 3765 | 497978 | < 0.0001 |
| miR-375 | 7 | 702458 | 61 | 501682 | 40 | 702425 | 28 | 501715 | < 0.0001 |
| miR-523 | 8 | 702457 | 62 | 501681 | 41 | 702424 | 29 | 501714 | < 0.0001 |
| miR-154* | 131 | 702334 | 218 | 501525 | 204 | 702261 | 145 | 501598 | < 0.0001 |
| miR-485-5p | 165 | 702300 | 26 | 501717 | 111 | 702354 | 80 | 501663 | < 0.0001 |
| miR-2110 | 110 | 702355 | 7 | 501736 | 68 | 702397 | 49 | 501694 | < 0.0001 |
| miR-193b | 392 | 702073 | 129 | 501614 | 304 | 702161 | 217 | 501526 | < 0.0001 |
| miR-1301 | 277 | 702188 | 74 | 501669 | 205 | 702260 | 146 | 501597 | < 0.0001 |
| miR-655 | 90 | 702375 | 170 | 501573 | 152 | 702313 | 108 | 501635 | < 0.0001 |
| miR-518c* | 3 | 702462 | 49 | 501694 | 30 | 702435 | 22 | 501721 | < 0.0001 |
| miR-519e* | 43 | 702422 | 108 | 501635 | 88 | 702377 | 63 | 501680 | < 0.0001 |
| miR-590-5p | 28 | 702437 | 85 | 501658 | 66 | 702399 | 47 | 501696 | < 0.0001 |
| miR-30e* | 1783 | 700682 | 1630 | 500113 | 1991 | 700474 | 1422 | 500321 | < 0.0001 |
| miR-99a* | 137 | 702328 | 22 | 501721 | 93 | 702372 | 66 | 501677 | < 0.0001 |
| miR-548x | 9 | 702456 | 54 | 501689 | 37 | 702428 | 26 | 501717 | < 0.0001 |
| miR-615-3p | 12 | 702453 | 59 | 501684 | 41 | 702424 | 30 | 501713 | < 0.0001 |
| miR-24 | 1016 | 701449 | 992 | 500751 | 1171 | 701294 | 837 | 500906 | < 0.0001 |
| miR-152 | 179 | 702286 | 251 | 501492 | 251 | 702214 | 179 | 501564 | < 0.0001 |
| miR-744 | 315 | 702150 | 104 | 501639 | 244 | 702221 | 175 | 501568 | < 0.0001 |

| | | | | | | | | | |
|---|---|---|---|---|---|---|---|---|---|
| miR-29a | 4952 | 697513 | 4096 | 497647 | 5278 | 697187 | 3770 | 497973 | < 0.0001 |
| miR-505 | 76 | 702389 | 141 | 501602 | 127 | 702338 | 90 | 501653 | < 0.0001 |
| miR-210 | 263 | 702202 | 79 | 501664 | 200 | 702265 | 142 | 501601 | < 0.0001 |
| miR-520g | 7 | 702458 | 49 | 501694 | 33 | 702432 | 23 | 501720 | < 0.0001 |
| miR-185 | 1096 | 701369 | 1052 | 500691 | 1253 | 701212 | 895 | 500848 | < 0.0001 |
| miR-382 | 257 | 702208 | 78 | 501665 | 195 | 702270 | 140 | 501603 | < 0.0001 |
| miR-200a | 2 | 702463 | 37 | 501706 | 23 | 702442 | 16 | 501727 | < 0.0001 |
| miR-302c* | 2 | 702463 | 37 | 501706 | 23 | 702442 | 16 | 501727 | < 0.0001 |
| miR-1269 | 4 | 702461 | 41 | 501702 | 26 | 702439 | 19 | 501724 | < 0.0001 |
| miR-151-3p | 740 | 701725 | 342 | 501401 | 631 | 701834 | 451 | 501292 | < 0.0001 |
| miR-654-3p | 146 | 702319 | 210 | 501533 | 208 | 702257 | 148 | 501595 | < 0.0001 |
| miR-199b-5p | 347 | 702118 | 401 | 501342 | 436 | 702029 | 312 | 501431 | < 0.0001 |
| miR-130b* | 8 | 702457 | 46 | 501697 | 32 | 702433 | 22 | 501721 | < 0.0001 |
| miR-629 | 401 | 702064 | 157 | 501586 | 326 | 702139 | 232 | 501511 | < 0.0001 |
| miR-122 | 54 | 702411 | 108 | 501635 | 95 | 702370 | 67 | 501676 | < 0.0001 |
| miR-509-5p | 71 | 702394 | 4 | 501739 | 44 | 702421 | 31 | 501712 | < 0.0001 |
| miR-7-1* | 89 | 702376 | 146 | 501597 | 137 | 702328 | 98 | 501645 | < 0.0001 |
| miR-378c | 29 | 702436 | 75 | 501668 | 61 | 702404 | 43 | 501700 | < 0.0001 |
| miR-129-5p | 81 | 702384 | 8 | 501735 | 52 | 702413 | 37 | 501706 | < 0.0001 |
| miR-320b | 231 | 702234 | 73 | 501670 | 177 | 702288 | 127 | 501616 | < 0.0001 |
| miR-135a | 495 | 701970 | 521 | 501222 | 593 | 701872 | 423 | 501320 | < 0.0001 |
| miR-302d* | 43 | 702422 | 91 | 501652 | 78 | 702387 | 56 | 501687 | < 0.0001 |
| miR-362-3p | 56 | 702409 | 105 | 501638 | 94 | 702371 | 67 | 501676 | < 0.0001 |
| miR-487b | 242 | 702223 | 81 | 501662 | 188 | 702277 | 135 | 501608 | < 0.0001 |
| miR-373* | 181 | 702284 | 52 | 501691 | 136 | 702329 | 97 | 501646 | < 0.0001 |
| miR-18a* | 17 | 702448 | 55 | 501688 | 42 | 702423 | 30 | 501713 | < 0.0001 |
| miR-542-3p | 396 | 702069 | 166 | 501577 | 328 | 702137 | 234 | 501509 | < 0.0001 |
| miR-16-2* | 60 | 702405 | 106 | 501637 | 97 | 702368 | 69 | 501674 | < 0.0001 |
| miR-133a | 19 | 702446 | 56 | 501687 | 44 | 702421 | 31 | 501712 | < 0.0001 |
| miR-518d-3p | 8 | 702457 | 39 | 501704 | 27 | 702438 | 20 | 501723 | < 0.0001 |

| | | | | | | | | | |
|---|---|---|---|---|---|---|---|---|---|
| miR-339-5p | 39 | 702426 | 81 | 501662 | 70 | 702395 | 50 | 501693 | < 0.0001 |
| miR-125a-3p | 151 | 702314 | 41 | 501702 | 112 | 702353 | 80 | 501663 | < 0.0001 |
| miR-380 | 54 | 702411 | 98 | 501645 | 89 | 702376 | 63 | 501680 | < 0.0001 |
| miR-1296 | 9 | 702456 | 40 | 501703 | 29 | 702436 | 20 | 501723 | < 0.0001 |
| miR-181b | 231 | 702234 | 82 | 501661 | 183 | 702282 | 130 | 501613 | < 0.0001 |
| miR-125a-5p | 3006 | 699459 | 1823 | 499920 | 2817 | 699648 | 2012 | 499731 | < 0.0001 |
| miR-521 | 4 | 702461 | 30 | 501713 | 20 | 702445 | 14 | 501729 | < 0.0001 |
| miR-1908 | 65 | 702400 | 7 | 501736 | 42 | 702423 | 30 | 501713 | < 0.0001 |
| miR-642b | 6 | 702459 | 33 | 501710 | 23 | 702442 | 16 | 501727 | < 0.0001 |
| miR-411* | 49 | 702416 | 89 | 501654 | 81 | 702384 | 57 | 501686 | < 0.0001 |
| miR-522 | 8 | 702457 | 36 | 501707 | 26 | 702439 | 18 | 501725 | < 0.0001 |
| miR-3609 | 4 | 702461 | 29 | 501714 | 19 | 702446 | 14 | 501729 | < 0.0001 |
| miR-493* | 1089 | 701376 | 594 | 501149 | 982 | 701483 | 701 | 501042 | < 0.0001 |
| miR-184 | 127 | 702338 | 166 | 501577 | 171 | 702294 | 122 | 501621 | < 0.0001 |
| miR-874 | 138 | 702327 | 40 | 501703 | 104 | 702361 | 74 | 501669 | < 0.0001 |
| miR-517b | 2 | 702463 | 23 | 501720 | 15 | 702450 | 10 | 501733 | < 0.0001 |
| miR-204 | 1198 | 701267 | 671 | 501072 | 1090 | 701375 | 779 | 500964 | < 0.0001 |
| miR-20a* | 109 | 702356 | 146 | 501597 | 149 | 702316 | 106 | 501637 | < 0.0001 |
| miR-221* | 5 | 702460 | 28 | 501715 | 19 | 702446 | 14 | 501729 | < 0.0001 |
| miR-222 | 580 | 701885 | 289 | 501454 | 507 | 701958 | 362 | 501381 | < 0.0001 |
| miR-520e | 4 | 702461 | 26 | 501717 | 18 | 702447 | 12 | 501731 | < 0.0001 |
| miR-520d-5p | 2 | 702463 | 22 | 501721 | 14 | 702451 | 10 | 501733 | < 0.0001 |
| miR-26b* | 8 | 702457 | 32 | 501711 | 23 | 702442 | 17 | 501726 | < 0.0001 |
| miR-488 | 10 | 702455 | 35 | 501708 | 26 | 702439 | 19 | 501724 | < 0.0001 |
| miR-362-5p | 93 | 702372 | 22 | 501721 | 67 | 702398 | 48 | 501695 | < 0.0001 |
| miR-20a | 357 | 702108 | 365 | 501378 | 421 | 702044 | 301 | 501442 | < 0.0001 |
| miR-30b* | 73 | 702392 | 14 | 501729 | 51 | 702414 | 36 | 501707 | < 0.0001 |
| miR-525-3p | 2 | 702463 | 21 | 501722 | 13 | 702452 | 10 | 501733 | < 0.0001 |
| miR-642a | 2 | 702463 | 21 | 501722 | 13 | 702452 | 10 | 501733 | < 0.0001 |
| miR-449a | 45 | 702420 | 4 | 501739 | 29 | 702436 | 20 | 501723 | < 0.0001 |

| | | | | | | | | | |
|---|---|---|---|---|---|---|---|---|---|
| miR-370 | 59 | 702406 | 9 | 501734 | 40 | 702425 | 28 | 501715 | < 0.0001 |
| **Additional miRNAs detected (P>0.0001)** | | | | | | | | | |
| miR-378 | 1544 | 700921 | 907 | 500836 | 1430 | 701035 | 1021 | 500722 | 0,0002 |
| miR-524-3p | 8 | 702457 | 30 | 501713 | 22 | 702443 | 16 | 501727 | 0,0002 |
| miR-140-5p | 933 | 701532 | 518 | 501225 | 846 | 701619 | 605 | 501138 | 0,0003 |
| miR-425 | 75 | 702390 | 106 | 501637 | 106 | 702359 | 75 | 501668 | 0,0003 |
| miR-323b-3p | 78 | 702387 | 18 | 501725 | 56 | 702409 | 40 | 501703 | 0,0004 |
| miR-369-3p | 2217 | 700248 | 1825 | 499918 | 2358 | 700107 | 1684 | 500059 | 0,0004 |
| miR-376b | 591 | 701874 | 550 | 501193 | 666 | 701799 | 475 | 501268 | 0,0005 |
| miR-664 | 19 | 702446 | 43 | 501700 | 36 | 702429 | 26 | 501717 | 0,0006 |
| miR-124 | 54 | 702411 | 82 | 501661 | 79 | 702386 | 57 | 501686 | 0,0007 |
| miR-31* | 2 | 702463 | 18 | 501725 | 12 | 702453 | 8 | 501735 | 0,0007 |
| miR-98 | 756 | 701709 | 680 | 501063 | 838 | 701627 | 598 | 501145 | 0,0007 |
| miR-17* | 513 | 701952 | 264 | 501479 | 453 | 702012 | 324 | 501419 | 0,0008 |
| miR-425* | 179 | 702286 | 199 | 501544 | 221 | 702244 | 157 | 501586 | 0,0009 |
| miR-455-3p | 75 | 702390 | 102 | 501641 | 103 | 702362 | 74 | 501669 | 0,0010 |
| miR-1277 | 8 | 702457 | 27 | 501716 | 20 | 702445 | 15 | 501728 | 0,0012 |
| miR-196b | 2 | 702463 | 17 | 501726 | 11 | 702454 | 8 | 501735 | 0,0013 |
| miR-194 | 24 | 702441 | 47 | 501696 | 41 | 702424 | 30 | 501713 | 0,0015 |
| hsa-let-7f | 24583 | 677882 | 16862 | 484881 | 24177 | 678288 | 17268 | 484475 | 0,0020 |
| miR-383 | 42 | 702423 | 6 | 501737 | 28 | 702437 | 20 | 501723 | 0,0021 |
| miR-505* | 3 | 702462 | 18 | 501725 | 12 | 702453 | 9 | 501734 | 0,0021 |
| miR-514b-3p | 36 | 702429 | 4 | 501739 | 23 | 702442 | 17 | 501726 | 0,0024 |
| miR-379 | 169 | 702296 | 68 | 501675 | 138 | 702327 | 99 | 501644 | 0,0025 |
| miR-3180-3p | 72 | 702393 | 19 | 501724 | 53 | 702412 | 38 | 501705 | 0,0028 |
| miR-491-5p | 41 | 702424 | 6 | 501737 | 27 | 702438 | 20 | 501723 | 0,0028 |
| miR-409-5p | 78 | 702387 | 22 | 501721 | 58 | 702407 | 42 | 501701 | 0,0031 |
| miR-342-5p | 50 | 702415 | 10 | 501733 | 35 | 702430 | 25 | 501718 | 0,0039 |
| miR-32* | 65 | 702400 | 87 | 501656 | 89 | 702376 | 63 | 501680 | 0,0044 |

| | | | | | | | | | |
|---|---|---|---|---|---|---|---|---|---|
| miR-154 | 235 | 702230 | 107 | 501636 | 200 | 702265 | 142 | 501601 | 0,0044 |
| miR-889 | 159 | 702306 | 65 | 501678 | 131 | 702334 | 93 | 501650 | 0,0053 |
| hsa-let-7i | 7560 | 694905 | 5771 | 495972 | 7777 | 694688 | 5554 | 496189 | 0,0055 |
| miR-181a | 2632 | 699833 | 1670 | 500073 | 2510 | 699955 | 1792 | 499951 | 0,0061 |
| miR-299-5p | 103 | 702362 | 121 | 501622 | 131 | 702334 | 93 | 501650 | 0,0071 |
| miR-190b | 35 | 702430 | 5 | 501738 | 23 | 702442 | 17 | 501726 | 0,0073 |
| miR-196a | 7 | 702458 | 22 | 501721 | 17 | 702448 | 12 | 501731 | 0,0074 |
| miR-624* | 2 | 702463 | 14 | 501729 | 9 | 702456 | 7 | 501736 | 0,0079 |
| miR-454 | 90 | 702375 | 30 | 501713 | 70 | 702395 | 50 | 501693 | 0,0083 |
| miR-30a* | 546 | 701919 | 490 | 501253 | 604 | 701861 | 432 | 501311 | 0,0090 |
| miR-532-3p | 111 | 702354 | 127 | 501616 | 139 | 702326 | 99 | 501644 | 0,0095 |
| miR-17 | 435 | 702030 | 231 | 501512 | 389 | 702076 | 277 | 501466 | 0,0096 |
| miR-147b | 7 | 702458 | 21 | 501722 | 16 | 702449 | 12 | 501731 | 0,0123 |
| miR-493 | 357 | 702108 | 185 | 501558 | 316 | 702149 | 226 | 501517 | 0,0131 |
| miR-200c* | 2 | 702463 | 13 | 501730 | 9 | 702456 | 6 | 501737 | 0,0140 |
| hsa-let-7b* | 229 | 702236 | 109 | 501634 | 197 | 702268 | 141 | 501602 | 0,0150 |
| miR-326 | 24 | 702441 | 41 | 501702 | 38 | 702427 | 27 | 501716 | 0,0155 |
| miR-181a-2* | 6 | 702459 | 19 | 501724 | 15 | 702450 | 10 | 501733 | 0,0164 |
| miR-520f | 4 | 702461 | 16 | 501727 | 12 | 702453 | 8 | 501735 | 0,0167 |
| miR-548y | 4 | 702461 | 16 | 501727 | 12 | 702453 | 8 | 501735 | 0,0167 |
| miR-183* | 43 | 702422 | 60 | 501683 | 60 | 702405 | 43 | 501700 | 0,0201 |
| miR-135a* | 28 | 702437 | 4 | 501739 | 19 | 702446 | 13 | 501730 | 0,0244 |
| miR-329 | 44 | 702421 | 60 | 501683 | 61 | 702404 | 43 | 501700 | 0,0267 |
| miR-1249 | 20 | 702445 | 35 | 501708 | 32 | 702433 | 23 | 501720 | 0,0274 |
| miR-1468 | 35 | 702430 | 7 | 501736 | 25 | 702440 | 17 | 501726 | 0,0289 |
| miR-95 | 207 | 702258 | 204 | 501539 | 240 | 702225 | 171 | 501572 | 0,0296 |
| miR-548c-5p | 11 | 702454 | 24 | 501719 | 20 | 702445 | 15 | 501728 | 0,0339 |
| miR-504 | 3 | 702462 | 13 | 501730 | 9 | 702456 | 7 | 501736 | 0,0354 |
| miR-576-3p | 8 | 702457 | 20 | 501723 | 16 | 702449 | 12 | 501731 | 0,0371 |
| miR-497* | 58 | 702407 | 18 | 501725 | 44 | 702421 | 32 | 501711 | 0,0386 |

| | | | | | | | | | |
|---|---|---|---|---|---|---|---|---|---|
| miR-20b | 13 | 702452 | 26 | 501717 | 23 | 702442 | 16 | 501727 | 0,0399 |
| miR-543 | 197 | 702268 | 95 | 501648 | 170 | 702295 | 122 | 501621 | 0,0401 |
| miR-144* | 2 | 702463 | 11 | 501732 | 8 | 702457 | 5 | 501738 | 0,0427 |
| miR-301b | 2 | 702463 | 11 | 501732 | 8 | 702457 | 5 | 501738 | 0,0427 |
| miR-429 | 2 | 702463 | 11 | 501732 | 8 | 702457 | 5 | 501738 | 0,0427 |
| miR-519e | 2 | 702463 | 11 | 501732 | 8 | 702457 | 5 | 501738 | 0,0427 |
| miR-551a | 2 | 702463 | 11 | 501732 | 8 | 702457 | 5 | 501738 | 0,0427 |
| miR-625* | 2 | 702463 | 11 | 501732 | 8 | 702457 | 5 | 501738 | 0,0427 |
| miR-363 | 26 | 702439 | 40 | 501703 | 39 | 702426 | 27 | 501716 | 0,0450 |
| miR-942 | 10 | 702455 | 22 | 501721 | 19 | 702446 | 13 | 501730 | 0,0466 |
| miR-582-5p | 4 | 702461 | 14 | 501729 | 11 | 702454 | 7 | 501736 | 0,0466 |
| miR-125b-1* | 71 | 702394 | 25 | 501718 | 56 | 702409 | 40 | 501703 | 0,0469 |
| miR-485-3p | 33 | 702432 | 47 | 501696 | 47 | 702418 | 33 | 501710 | 0,0476 |
| miR-376a* | 65 | 702400 | 22 | 501721 | 51 | 702414 | 36 | 501707 | 0,0477 |
| miR-550a* | 12 | 702453 | 24 | 501719 | 21 | 702444 | 15 | 501728 | 0,0550 |
| miR-455-5p | 33 | 702432 | 46 | 501697 | 46 | 702419 | 33 | 501710 | 0,0632 |
| miR-331-3p | 67 | 702398 | 78 | 501665 | 85 | 702380 | 60 | 501683 | 0,0670 |
| miR-1287 | 29 | 702436 | 6 | 501737 | 20 | 702445 | 15 | 501728 | 0,0702 |
| miR-34c-3p | 29 | 702436 | 6 | 501737 | 20 | 702445 | 15 | 501728 | 0,0702 |
| miR-155* | 2 | 702463 | 10 | 501733 | 7 | 702458 | 5 | 501738 | 0,0727 |
| miR-556-5p | 2 | 702463 | 10 | 501733 | 7 | 702458 | 5 | 501738 | 0,0727 |
| miR-2114 | 7 | 702458 | 17 | 501726 | 14 | 702451 | 10 | 501733 | 0,0780 |
| miR-34b* | 26 | 702439 | 5 | 501738 | 18 | 702447 | 13 | 501730 | 0,0806 |
| miR-15b* | 36 | 702429 | 48 | 501695 | 49 | 702416 | 35 | 501708 | 0,0819 |
| miR-29c | 3474 | 698991 | 2301 | 499442 | 3369 | 699096 | 2406 | 499337 | 0,0945 |
| miR-935 | 3 | 702462 | 11 | 501732 | 8 | 702457 | 6 | 501737 | 0,0974 |
| miR-92b | 235 | 702230 | 218 | 501525 | 264 | 702201 | 189 | 501554 | 0,1002 |
| miR-532-5p | 158 | 702307 | 77 | 501666 | 137 | 702328 | 98 | 501645 | 0,1049 |
| miR-629* | 6 | 702459 | 15 | 501728 | 12 | 702453 | 9 | 501734 | 0,1051 |
| miR-652 | 188 | 702277 | 95 | 501648 | 165 | 702300 | 118 | 501625 | 0,1059 |

| | | | | | | | | | |
|---|---|---|---|---|---|---|---|---|---|
| miR-101* | 21 | 702444 | 32 | 501711 | 31 | 702434 | 22 | 501721 | 0,1059 |
| miR-501-3p | 23 | 702442 | 34 | 501709 | 33 | 702432 | 24 | 501719 | 0,1080 |
| miR-296-5p | 11 | 702454 | 21 | 501722 | 19 | 702446 | 13 | 501730 | 0,1092 |
| miR-139-5p | 51 | 702414 | 61 | 501682 | 65 | 702400 | 47 | 501696 | 0,1096 |
| miR-486-3p | 36 | 702429 | 10 | 501733 | 27 | 702438 | 19 | 501724 | 0,1110 |
| miR-2355-5p | 4 | 702461 | 12 | 501731 | 9 | 702456 | 7 | 501736 | 0,1202 |
| miR-576-5p | 4 | 702461 | 12 | 501731 | 9 | 702456 | 7 | 501736 | 0,1202 |
| miR-598 | 4 | 702461 | 12 | 501731 | 9 | 702456 | 7 | 501736 | 0,1202 |
| miR-106a | 2 | 702463 | 9 | 501734 | 6 | 702459 | 5 | 501738 | 0,1210 |
| miR-1270 | 2 | 702463 | 9 | 501734 | 6 | 702459 | 5 | 501738 | 0,1210 |
| miR-191* | 2 | 702463 | 9 | 501734 | 6 | 702459 | 5 | 501738 | 0,1210 |
| miR-1912 | 2 | 702463 | 9 | 501734 | 6 | 702459 | 5 | 501738 | 0,1210 |
| miR-100 | 1036 | 701429 | 837 | 500906 | 1093 | 701372 | 780 | 500963 | 0,1334 |
| miR-671-5p | 43 | 702422 | 14 | 501729 | 33 | 702432 | 24 | 501719 | 0,1434 |
| miR-96* | 47 | 702418 | 56 | 501687 | 60 | 702405 | 43 | 501700 | 0,1446 |
| miR-1185 | 30 | 702435 | 8 | 501735 | 22 | 702443 | 16 | 501727 | 0,1560 |
| miR-205* | 6 | 702459 | 14 | 501729 | 12 | 702453 | 8 | 501735 | 0,1582 |
| miR-149 | 45 | 702420 | 53 | 501690 | 57 | 702408 | 41 | 501702 | 0,1836 |
| miR-187 | 27 | 702438 | 7 | 501736 | 20 | 702445 | 14 | 501729 | 0,1837 |
| miR-3158 | 4 | 702461 | 11 | 501732 | 9 | 702456 | 6 | 501737 | 0,1855 |
| miR-548e | 4 | 702461 | 11 | 501732 | 9 | 702456 | 6 | 501737 | 0,1855 |
| miR-3606 | 8 | 702457 | 16 | 501727 | 14 | 702451 | 10 | 501733 | 0,1867 |
| miR-450b-5p | 235 | 702230 | 128 | 501615 | 212 | 702253 | 151 | 501592 | 0,1899 |
| miR-1251 | 2 | 702463 | 8 | 501735 | 6 | 702459 | 4 | 501739 | 0,1957 |
| miR-200a* | 2 | 702463 | 8 | 501735 | 6 | 702459 | 4 | 501739 | 0,1957 |
| miR-3647-5p | 2 | 702463 | 8 | 501735 | 6 | 702459 | 4 | 501739 | 0,1957 |
| miR-548b-3p | 2 | 702463 | 8 | 501735 | 6 | 702459 | 4 | 501739 | 0,1957 |
| miR-29b-1* | 47 | 702418 | 17 | 501726 | 37 | 702428 | 27 | 501716 | 0,1986 |
| miR-214* | 50 | 702415 | 57 | 501686 | 62 | 702403 | 45 | 501698 | 0,2045 |
| miR-132 | 75 | 702390 | 79 | 501664 | 90 | 702375 | 64 | 501679 | 0,2083 |

| | | | | | | | | | |
|---|---|---|---|---|---|---|---|---|---|
| miR-1228 | 5 | 702460 | 12 | 501731 | 10 | 702455 | 7 | 501736 | 0,2106 |
| hsa-let-7d* | 130 | 702335 | 125 | 501618 | 149 | 702316 | 106 | 501637 | 0,2248 |
| hsa-let-7g* | 30 | 702435 | 9 | 501734 | 23 | 702442 | 16 | 501727 | 0,2358 |
| miR-99b | 575 | 701890 | 474 | 501269 | 612 | 701853 | 437 | 501306 | 0,2530 |
| miR-3614-5p | 8 | 702457 | 15 | 501728 | 13 | 702452 | 10 | 501733 | 0,2627 |
| miR-1255b | 4 | 702461 | 10 | 501733 | 8 | 702457 | 6 | 501737 | 0,2769 |
| miR-3660 | 4 | 702461 | 10 | 501733 | 8 | 702457 | 6 | 501737 | 0,2769 |
| miR-323-3p | 103 | 702362 | 50 | 501693 | 89 | 702376 | 64 | 501679 | 0,2788 |
| hsa-let-7a* | 186 | 702279 | 101 | 501642 | 167 | 702298 | 120 | 501623 | 0,2924 |
| miR-30d* | 5 | 702460 | 11 | 501732 | 9 | 702456 | 7 | 501736 | 0,3053 |
| miR-106a* | 2 | 702463 | 7 | 501736 | 5 | 702460 | 4 | 501739 | 0,3053 |
| miR-138 | 2 | 702463 | 7 | 501736 | 5 | 702460 | 4 | 501739 | 0,3053 |
| miR-148a* | 2 | 702463 | 7 | 501736 | 5 | 702460 | 4 | 501739 | 0,3053 |
| miR-2115* | 2 | 702463 | 7 | 501736 | 5 | 702460 | 4 | 501739 | 0,3053 |
| miR-3200-3p | 2 | 702463 | 7 | 501736 | 5 | 702460 | 4 | 501739 | 0,3053 |
| miR-3940 | 2 | 702463 | 7 | 501736 | 5 | 702460 | 4 | 501739 | 0,3053 |
| miR-548a-3p | 2 | 702463 | 7 | 501736 | 5 | 702460 | 4 | 501739 | 0,3053 |
| miR-548i | 2 | 702463 | 7 | 501736 | 5 | 702460 | 4 | 501739 | 0,3053 |
| miR-579 | 2 | 702463 | 7 | 501736 | 5 | 702460 | 4 | 501739 | 0,3053 |
| miR-891b | 2 | 702463 | 7 | 501736 | 5 | 702460 | 4 | 501739 | 0,3053 |
| miR-29b | 2174 | 700291 | 1667 | 500076 | 2241 | 700224 | 1600 | 500143 | 0,3119 |
| miR-19b-1* | 6 | 702459 | 12 | 501731 | 11 | 702454 | 7 | 501736 | 0,3275 |
| miR-33b | 6 | 702459 | 12 | 501731 | 11 | 702454 | 7 | 501736 | 0,3275 |
| miR-1247 | 49 | 702416 | 20 | 501723 | 40 | 702425 | 29 | 501714 | 0,3349 |
| miR-93* | 59 | 702406 | 62 | 501681 | 71 | 702394 | 50 | 501693 | 0,3351 |
| miR-708 | 198 | 702267 | 110 | 501633 | 180 | 702285 | 128 | 501615 | 0,3438 |
| miR-223* | 7 | 702458 | 13 | 501730 | 12 | 702453 | 8 | 501735 | 0,3449 |
| miR-487a | 56 | 702409 | 24 | 501719 | 47 | 702418 | 33 | 501710 | 0,3450 |
| miR-548d-5p | 24 | 702441 | 30 | 501713 | 32 | 702433 | 22 | 501721 | 0,3686 |
| miR-195* | 39 | 702426 | 15 | 501728 | 32 | 702433 | 22 | 501721 | 0,3688 |

| | | | | | | | | | |
|---|---|---|---|---|---|---|---|---|---|
| miR-139-3p | 37 | 702428 | 14 | 501729 | 30 | 702435 | 21 | 501722 | 0,3745 |
| miR-374a* | 129 | 702336 | 119 | 501624 | 145 | 702320 | 103 | 501640 | 0,3961 |
| miR-3687 | 4 | 702461 | 9 | 501734 | 8 | 702457 | 5 | 501738 | 0,3974 |
| miR-548o | 4 | 702461 | 9 | 501734 | 8 | 702457 | 5 | 501738 | 0,3974 |
| miR-760 | 16 | 702449 | 4 | 501739 | 12 | 702453 | 8 | 501735 | 0,4249 |
| miR-340* | 23 | 702442 | 28 | 501715 | 30 | 702435 | 21 | 501722 | 0,4516 |
| miR-1264 | 2 | 702463 | 6 | 501737 | 5 | 702460 | 3 | 501740 | 0,4544 |
| miR-1273e | 2 | 702463 | 6 | 501737 | 5 | 702460 | 3 | 501740 | 0,4544 |
| miR-146a* | 2 | 702463 | 6 | 501737 | 5 | 702460 | 3 | 501740 | 0,4544 |
| miR-18b | 2 | 702463 | 6 | 501737 | 5 | 702460 | 3 | 501740 | 0,4544 |
| miR-208a | 2 | 702463 | 6 | 501737 | 5 | 702460 | 3 | 501740 | 0,4544 |
| miR-20b* | 2 | 702463 | 6 | 501737 | 5 | 702460 | 3 | 501740 | 0,4544 |
| miR-217 | 2 | 702463 | 6 | 501737 | 5 | 702460 | 3 | 501740 | 0,4544 |
| miR-218-1* | 2 | 702463 | 6 | 501737 | 5 | 702460 | 3 | 501740 | 0,4544 |
| miR-24-2* | 2 | 702463 | 6 | 501737 | 5 | 702460 | 3 | 501740 | 0,4544 |
| miR-3065-3p | 2 | 702463 | 6 | 501737 | 5 | 702460 | 3 | 501740 | 0,4544 |
| miR-338-5p | 2 | 702463 | 6 | 501737 | 5 | 702460 | 3 | 501740 | 0,4544 |
| miR-3620 | 2 | 702463 | 6 | 501737 | 5 | 702460 | 3 | 501740 | 0,4544 |
| miR-4326 | 2 | 702463 | 6 | 501737 | 5 | 702460 | 3 | 501740 | 0,4544 |
| miR-548c-3p | 2 | 702463 | 6 | 501737 | 5 | 702460 | 3 | 501740 | 0,4544 |
| miR-548p | 2 | 702463 | 6 | 501737 | 5 | 702460 | 3 | 501740 | 0,4544 |
| miR-643 | 2 | 702463 | 6 | 501737 | 5 | 702460 | 3 | 501740 | 0,4544 |
| miR-550a | 24 | 702441 | 8 | 501735 | 19 | 702446 | 13 | 501730 | 0,4544 |
| miR-941 | 7 | 702458 | 12 | 501731 | 11 | 702454 | 8 | 501735 | 0,4612 |
| miR-374b* | 20 | 702445 | 25 | 501718 | 26 | 702439 | 19 | 501724 | 0,4670 |
| miR-125b-2* | 37 | 702428 | 15 | 501728 | 30 | 702435 | 22 | 501721 | 0,4754 |
| miR-1303 | 37 | 702428 | 15 | 501728 | 30 | 702435 | 22 | 501721 | 0,4754 |
| hsa-let-7e* | 9 | 702456 | 14 | 501729 | 13 | 702452 | 10 | 501733 | 0,4794 |
| miR-377* | 16 | 702449 | 21 | 501722 | 22 | 702443 | 15 | 501728 | 0,4829 |
| miR-544 | 15 | 702450 | 20 | 501723 | 20 | 702445 | 15 | 501728 | 0,4856 |

| | | | | | | | | | |
|---|---|---|---|---|---|---|---|---|---|
| miR-153 | 11 | 702454 | 16 | 501727 | 16 | 702449 | 11 | 501732 | 0,4873 |
| miR-548aa | 12 | 702453 | 17 | 501726 | 17 | 702448 | 12 | 501731 | 0,4886 |
| miR-22* | 263 | 702202 | 156 | 501587 | 244 | 702221 | 175 | 501568 | 0,4947 |
| miR-29c* | 150 | 702315 | 133 | 501610 | 165 | 702300 | 118 | 501625 | 0,5074 |
| miR-1179 | 3 | 702462 | 7 | 501736 | 6 | 702459 | 4 | 501739 | 0,5084 |
| miR-19a* | 3 | 702462 | 7 | 501736 | 6 | 702459 | 4 | 501739 | 0,5084 |
| miR-3614-3p | 3 | 702462 | 7 | 501736 | 6 | 702459 | 4 | 501739 | 0,5084 |
| miR-539 | 3 | 702462 | 7 | 501736 | 6 | 702459 | 4 | 501739 | 0,5084 |
| miR-559 | 3 | 702462 | 7 | 501736 | 6 | 702459 | 4 | 501739 | 0,5084 |
| miR-30c-2* | 24 | 702441 | 28 | 501715 | 30 | 702435 | 22 | 501721 | 0,5291 |
| miR-367* | 4 | 702461 | 8 | 501735 | 7 | 702458 | 5 | 501738 | 0,5435 |
| miR-3688 | 4 | 702461 | 8 | 501735 | 7 | 702458 | 5 | 501738 | 0,5435 |
| miR-625 | 34 | 702431 | 14 | 501729 | 28 | 702437 | 20 | 501723 | 0,5436 |
| miR-93 | 300 | 702165 | 182 | 501561 | 281 | 702184 | 201 | 501542 | 0,5532 |
| miR-410 | 39 | 702426 | 41 | 501702 | 47 | 702418 | 33 | 501710 | 0,5539 |
| miR-501-5p | 30 | 702435 | 33 | 501710 | 37 | 702428 | 26 | 501717 | 0,5618 |
| miR-106b* | 70 | 702395 | 67 | 501676 | 80 | 702385 | 57 | 501686 | 0,5655 |
| miR-1307 | 51 | 702414 | 51 | 501692 | 60 | 702405 | 42 | 501701 | 0,5721 |
| miR-876-5p | 6 | 702459 | 10 | 501733 | 9 | 702456 | 7 | 501736 | 0,5820 |
| miR-548n | 14 | 702451 | 4 | 501739 | 11 | 702454 | 7 | 501736 | 0,5919 |
| miR-502-5p | 22 | 702443 | 8 | 501735 | 18 | 702447 | 12 | 501731 | 0,5958 |
| miR-34c-5p | 16 | 702449 | 5 | 501738 | 12 | 702453 | 9 | 501734 | 0,5996 |
| miR-92b* | 18 | 702447 | 6 | 501737 | 14 | 702451 | 10 | 501733 | 0,6018 |
| miR-324-3p | 55 | 702410 | 27 | 501716 | 48 | 702417 | 34 | 501709 | 0,6310 |
| miR-1246 | 2 | 702463 | 5 | 501738 | 4 | 702461 | 3 | 501740 | 0,6355 |
| miR-1276 | 2 | 702463 | 5 | 501738 | 4 | 702461 | 3 | 501740 | 0,6355 |
| miR-1537 | 2 | 702463 | 5 | 501738 | 4 | 702461 | 3 | 501740 | 0,6355 |
| miR-1972 | 2 | 702463 | 5 | 501738 | 4 | 702461 | 3 | 501740 | 0,6355 |
| miR-3129 | 2 | 702463 | 5 | 501738 | 4 | 702461 | 3 | 501740 | 0,6355 |
| miR-3133 | 2 | 702463 | 5 | 501738 | 4 | 702461 | 3 | 501740 | 0,6355 |

| | | | | | | | | | |
|---|---|---|---|---|---|---|---|---|---|
| miR-3143 | 2 | 702463 | 5 | 501738 | 4 | 702461 | 3 | 501740 | 0,6355 |
| miR-3148 | 2 | 702463 | 5 | 501738 | 4 | 702461 | 3 | 501740 | 0,6355 |
| miR-3156 | 2 | 702463 | 5 | 501738 | 4 | 702461 | 3 | 501740 | 0,6355 |
| miR-3166 | 2 | 702463 | 5 | 501738 | 4 | 702461 | 3 | 501740 | 0,6355 |
| miR-3192 | 2 | 702463 | 5 | 501738 | 4 | 702461 | 3 | 501740 | 0,6355 |
| miR-323b-5p | 2 | 702463 | 5 | 501738 | 4 | 702461 | 3 | 501740 | 0,6355 |
| miR-330-5p | 2 | 702463 | 5 | 501738 | 4 | 702461 | 3 | 501740 | 0,6355 |
| miR-33b* | 2 | 702463 | 5 | 501738 | 4 | 702461 | 3 | 501740 | 0,6355 |
| miR-365* | 2 | 702463 | 5 | 501738 | 4 | 702461 | 3 | 501740 | 0,6355 |
| miR-3667-3p | 2 | 702463 | 5 | 501738 | 4 | 702461 | 3 | 501740 | 0,6355 |
| miR-3667-5p | 2 | 702463 | 5 | 501738 | 4 | 702461 | 3 | 501740 | 0,6355 |
| miR-3684 | 2 | 702463 | 5 | 501738 | 4 | 702461 | 3 | 501740 | 0,6355 |
| miR-3910 | 2 | 702463 | 5 | 501738 | 4 | 702461 | 3 | 501740 | 0,6355 |
| miR-3944 | 2 | 702463 | 5 | 501738 | 4 | 702461 | 3 | 501740 | 0,6355 |
| miR-412 | 2 | 702463 | 5 | 501738 | 4 | 702461 | 3 | 501740 | 0,6355 |
| miR-454* | 2 | 702463 | 5 | 501738 | 4 | 702461 | 3 | 501740 | 0,6355 |
| miR-491-3p | 2 | 702463 | 5 | 501738 | 4 | 702461 | 3 | 501740 | 0,6355 |
| miR-499-3p | 2 | 702463 | 5 | 501738 | 4 | 702461 | 3 | 501740 | 0,6355 |
| miR-548a-5p | 2 | 702463 | 5 | 501738 | 4 | 702461 | 3 | 501740 | 0,6355 |
| miR-548d-3p | 2 | 702463 | 5 | 501738 | 4 | 702461 | 3 | 501740 | 0,6355 |
| miR-548g | 2 | 702463 | 5 | 501738 | 4 | 702461 | 3 | 501740 | 0,6355 |
| miR-548j | 2 | 702463 | 5 | 501738 | 4 | 702461 | 3 | 501740 | 0,6355 |
| miR-548m | 2 | 702463 | 5 | 501738 | 4 | 702461 | 3 | 501740 | 0,6355 |
| miR-549 | 2 | 702463 | 5 | 501738 | 4 | 702461 | 3 | 501740 | 0,6355 |
| miR-552 | 2 | 702463 | 5 | 501738 | 4 | 702461 | 3 | 501740 | 0,6355 |
| miR-616* | 2 | 702463 | 5 | 501738 | 4 | 702461 | 3 | 501740 | 0,6355 |
| miR-624 | 2 | 702463 | 5 | 501738 | 4 | 702461 | 3 | 501740 | 0,6355 |
| miR-627 | 2 | 702463 | 5 | 501738 | 4 | 702461 | 3 | 501740 | 0,6355 |
| miR-875-5p | 2 | 702463 | 5 | 501738 | 4 | 702461 | 3 | 501740 | 0,6355 |
| miR-877* | 2 | 702463 | 5 | 501738 | 4 | 702461 | 3 | 501740 | 0,6355 |

| | | | | | | | | | |
|---|---|---|---|---|---|---|---|---|---|
| miR-885-5p | 2 | 702463 | 5 | 501738 | 4 | 702461 | 3 | 501740 | 0,6355 |
| miR-628-5p | 23 | 702442 | 9 | 501734 | 19 | 702446 | 13 | 501730 | 0,6601 |
| miR-499-5p | 48 | 702417 | 47 | 501696 | 55 | 702410 | 40 | 501703 | 0,6657 |
| miR-1304 | 3 | 702462 | 6 | 501737 | 5 | 702460 | 4 | 501739 | 0,6781 |
| miR-2113 | 3 | 702462 | 6 | 501737 | 5 | 702460 | 4 | 501739 | 0,6781 |
| miR-377 | 220 | 702245 | 133 | 501610 | 206 | 702259 | 147 | 501596 | 0,6787 |
| miR-369-5p | 40 | 702425 | 40 | 501703 | 47 | 702418 | 33 | 501710 | 0,6833 |
| miR-135b* | 15 | 702450 | 5 | 501738 | 12 | 702453 | 8 | 501735 | 0,6834 |
| miR-379* | 33 | 702432 | 34 | 501709 | 39 | 702426 | 28 | 501715 | 0,6857 |
| miR-548z | 14 | 702451 | 17 | 501726 | 18 | 702447 | 13 | 501730 | 0,6966 |
| miR-137 | 4 | 702461 | 7 | 501736 | 6 | 702459 | 5 | 501738 | 0,7018 |
| miR-192* | 4 | 702461 | 7 | 501736 | 6 | 702459 | 5 | 501738 | 0,7018 |
| miR-548b-5p | 4 | 702461 | 7 | 501736 | 6 | 702459 | 5 | 501738 | 0,7018 |
| miR-589 | 4 | 702461 | 7 | 501736 | 6 | 702459 | 5 | 501738 | 0,7018 |
| miR-33a* | 65 | 702400 | 34 | 501709 | 58 | 702407 | 41 | 501702 | 0,7019 |
| miR-181d | 13 | 702452 | 16 | 501727 | 17 | 702448 | 12 | 501731 | 0,7033 |
| miR-188-3p | 12 | 702453 | 15 | 501728 | 16 | 702449 | 11 | 501732 | 0,7094 |
| miR-3676 | 5 | 702460 | 8 | 501735 | 8 | 702457 | 5 | 501738 | 0,7151 |
| miR-423-3p | 24 | 702441 | 10 | 501733 | 20 | 702445 | 14 | 501729 | 0,7173 |
| miR-296-3p | 10 | 702455 | 13 | 501730 | 13 | 702452 | 10 | 501733 | 0,7195 |
| miR-3613-3p | 10 | 702455 | 13 | 501730 | 13 | 702452 | 10 | 501733 | 0,7195 |
| miR-3180-5p | 6 | 702459 | 9 | 501734 | 9 | 702456 | 6 | 501737 | 0,7221 |
| miR-3117 | 8 | 702457 | 11 | 501732 | 11 | 702454 | 8 | 501735 | 0,7249 |
| miR-665 | 56 | 702409 | 29 | 501714 | 50 | 702415 | 35 | 501708 | 0,7371 |
| miR-190 | 68 | 702397 | 62 | 501681 | 76 | 702389 | 54 | 501689 | 0,7463 |
| hsa-let-7i* | 44 | 702421 | 42 | 501701 | 50 | 702415 | 36 | 501707 | 0,7688 |
| miR-767-3p | 52 | 702413 | 48 | 501695 | 58 | 702407 | 42 | 501701 | 0,7996 |
| miR-3915 | 12 | 702453 | 14 | 501729 | 15 | 702450 | 11 | 501732 | 0,8111 |
| miR-191 | 5855 | 696610 | 4077 | 497666 | 5794 | 696671 | 4138 | 497605 | 0,8147 |
| miR-1178 | 2 | 702463 | 4 | 501739 | 4 | 702461 | 2 | 501741 | 0,8190 |

| | | | | | | | | | |
|---|---|---|---|---|---|---|---|---|---|
| miR-1181 | 2 | 702463 | 4 | 501739 | 4 | 702461 | 2 | 501741 | 0,8190 |
| miR-1182 | 2 | 702463 | 4 | 501739 | 4 | 702461 | 2 | 501741 | 0,8190 |
| miR-1183 | 2 | 702463 | 4 | 501739 | 4 | 702461 | 2 | 501741 | 0,8190 |
| miR-1184 | 2 | 702463 | 4 | 501739 | 4 | 702461 | 2 | 501741 | 0,8190 |
| miR-1193 | 2 | 702463 | 4 | 501739 | 4 | 702461 | 2 | 501741 | 0,8190 |
| miR-1200 | 2 | 702463 | 4 | 501739 | 4 | 702461 | 2 | 501741 | 0,8190 |
| miR-1202 | 2 | 702463 | 4 | 501739 | 4 | 702461 | 2 | 501741 | 0,8190 |
| miR-1203 | 2 | 702463 | 4 | 501739 | 4 | 702461 | 2 | 501741 | 0,8190 |
| miR-1204 | 2 | 702463 | 4 | 501739 | 4 | 702461 | 2 | 501741 | 0,8190 |
| miR-1205 | 2 | 702463 | 4 | 501739 | 4 | 702461 | 2 | 501741 | 0,8190 |
| miR-1206 | 2 | 702463 | 4 | 501739 | 4 | 702461 | 2 | 501741 | 0,8190 |
| miR-1207-3p | 2 | 702463 | 4 | 501739 | 4 | 702461 | 2 | 501741 | 0,8190 |
| miR-1207-5p | 2 | 702463 | 4 | 501739 | 4 | 702461 | 2 | 501741 | 0,8190 |
| miR-1208 | 2 | 702463 | 4 | 501739 | 4 | 702461 | 2 | 501741 | 0,8190 |
| miR-122* | 2 | 702463 | 4 | 501739 | 4 | 702461 | 2 | 501741 | 0,8190 |
| miR-1224-5p | 2 | 702463 | 4 | 501739 | 4 | 702461 | 2 | 501741 | 0,8190 |
| miR-1225-3p | 2 | 702463 | 4 | 501739 | 4 | 702461 | 2 | 501741 | 0,8190 |
| miR-1225-5p | 2 | 702463 | 4 | 501739 | 4 | 702461 | 2 | 501741 | 0,8190 |
| miR-1226 | 2 | 702463 | 4 | 501739 | 4 | 702461 | 2 | 501741 | 0,8190 |
| miR-1226* | 2 | 702463 | 4 | 501739 | 4 | 702461 | 2 | 501741 | 0,8190 |
| miR-1227 | 2 | 702463 | 4 | 501739 | 4 | 702461 | 2 | 501741 | 0,8190 |
| miR-1229 | 2 | 702463 | 4 | 501739 | 4 | 702461 | 2 | 501741 | 0,8190 |
| miR-1231 | 2 | 702463 | 4 | 501739 | 4 | 702461 | 2 | 501741 | 0,8190 |
| miR-1233 | 2 | 702463 | 4 | 501739 | 4 | 702461 | 2 | 501741 | 0,8190 |
| miR-1234 | 2 | 702463 | 4 | 501739 | 4 | 702461 | 2 | 501741 | 0,8190 |
| miR-1236 | 2 | 702463 | 4 | 501739 | 4 | 702461 | 2 | 501741 | 0,8190 |
| miR-1237 | 2 | 702463 | 4 | 501739 | 4 | 702461 | 2 | 501741 | 0,8190 |
| miR-1238 | 2 | 702463 | 4 | 501739 | 4 | 702461 | 2 | 501741 | 0,8190 |
| miR-1244 | 2 | 702463 | 4 | 501739 | 4 | 702461 | 2 | 501741 | 0,8190 |
| miR-1245 | 2 | 702463 | 4 | 501739 | 4 | 702461 | 2 | 501741 | 0,8190 |

| | | | | | | | | | |
|---|---|---|---|---|---|---|---|---|---|
| miR-1252 | 2 | 702463 | 4 | 501739 | 4 | 702461 | 2 | 501741 | 0,8190 |
| miR-1256 | 2 | 702463 | 4 | 501739 | 4 | 702461 | 2 | 501741 | 0,8190 |
| miR-1257 | 2 | 702463 | 4 | 501739 | 4 | 702461 | 2 | 501741 | 0,8190 |
| miR-1260 | 2 | 702463 | 4 | 501739 | 4 | 702461 | 2 | 501741 | 0,8190 |
| miR-1260b | 2 | 702463 | 4 | 501739 | 4 | 702461 | 2 | 501741 | 0,8190 |
| miR-1261 | 2 | 702463 | 4 | 501739 | 4 | 702461 | 2 | 501741 | 0,8190 |
| miR-1263 | 2 | 702463 | 4 | 501739 | 4 | 702461 | 2 | 501741 | 0,8190 |
| miR-1265 | 2 | 702463 | 4 | 501739 | 4 | 702461 | 2 | 501741 | 0,8190 |
| miR-1268 | 2 | 702463 | 4 | 501739 | 4 | 702461 | 2 | 501741 | 0,8190 |
| miR-1272 | 2 | 702463 | 4 | 501739 | 4 | 702461 | 2 | 501741 | 0,8190 |
| miR-1273 | 2 | 702463 | 4 | 501739 | 4 | 702461 | 2 | 501741 | 0,8190 |
| miR-1273c | 2 | 702463 | 4 | 501739 | 4 | 702461 | 2 | 501741 | 0,8190 |
| miR-1274a | 2 | 702463 | 4 | 501739 | 4 | 702461 | 2 | 501741 | 0,8190 |
| miR-1274b | 2 | 702463 | 4 | 501739 | 4 | 702461 | 2 | 501741 | 0,8190 |
| miR-1275 | 2 | 702463 | 4 | 501739 | 4 | 702461 | 2 | 501741 | 0,8190 |
| miR-1278 | 2 | 702463 | 4 | 501739 | 4 | 702461 | 2 | 501741 | 0,8190 |
| miR-1279 | 2 | 702463 | 4 | 501739 | 4 | 702461 | 2 | 501741 | 0,8190 |
| miR-1280 | 2 | 702463 | 4 | 501739 | 4 | 702461 | 2 | 501741 | 0,8190 |
| miR-1281 | 2 | 702463 | 4 | 501739 | 4 | 702461 | 2 | 501741 | 0,8190 |
| miR-1282 | 2 | 702463 | 4 | 501739 | 4 | 702461 | 2 | 501741 | 0,8190 |
| miR-1284 | 2 | 702463 | 4 | 501739 | 4 | 702461 | 2 | 501741 | 0,8190 |
| miR-1286 | 2 | 702463 | 4 | 501739 | 4 | 702461 | 2 | 501741 | 0,8190 |
| miR-1288 | 2 | 702463 | 4 | 501739 | 4 | 702461 | 2 | 501741 | 0,8190 |
| miR-1289 | 2 | 702463 | 4 | 501739 | 4 | 702461 | 2 | 501741 | 0,8190 |
| miR-1290 | 2 | 702463 | 4 | 501739 | 4 | 702461 | 2 | 501741 | 0,8190 |
| miR-1291 | 2 | 702463 | 4 | 501739 | 4 | 702461 | 2 | 501741 | 0,8190 |
| miR-1292 | 2 | 702463 | 4 | 501739 | 4 | 702461 | 2 | 501741 | 0,8190 |
| miR-1293 | 2 | 702463 | 4 | 501739 | 4 | 702461 | 2 | 501741 | 0,8190 |
| miR-1297 | 2 | 702463 | 4 | 501739 | 4 | 702461 | 2 | 501741 | 0,8190 |
| miR-1298 | 2 | 702463 | 4 | 501739 | 4 | 702461 | 2 | 501741 | 0,8190 |

| | | | | | | | | | |
|---|---|---|---|---|---|---|---|---|---|
| miR-1302 | 2 | 702463 | 4 | 501739 | 4 | 702461 | 2 | 501741 | 0,8190 |
| miR-1305 | 2 | 702463 | 4 | 501739 | 4 | 702461 | 2 | 501741 | 0,8190 |
| miR-1306 | 2 | 702463 | 4 | 501739 | 4 | 702461 | 2 | 501741 | 0,8190 |
| miR-130a* | 2 | 702463 | 4 | 501739 | 4 | 702461 | 2 | 501741 | 0,8190 |
| miR-1321 | 2 | 702463 | 4 | 501739 | 4 | 702461 | 2 | 501741 | 0,8190 |
| miR-1322 | 2 | 702463 | 4 | 501739 | 4 | 702461 | 2 | 501741 | 0,8190 |
| miR-1324 | 2 | 702463 | 4 | 501739 | 4 | 702461 | 2 | 501741 | 0,8190 |
| miR-133b | 2 | 702463 | 4 | 501739 | 4 | 702461 | 2 | 501741 | 0,8190 |
| miR-138-1* | 2 | 702463 | 4 | 501739 | 4 | 702461 | 2 | 501741 | 0,8190 |
| miR-138-2* | 2 | 702463 | 4 | 501739 | 4 | 702461 | 2 | 501741 | 0,8190 |
| miR-1469 | 2 | 702463 | 4 | 501739 | 4 | 702461 | 2 | 501741 | 0,8190 |
| miR-147 | 2 | 702463 | 4 | 501739 | 4 | 702461 | 2 | 501741 | 0,8190 |
| miR-1470 | 2 | 702463 | 4 | 501739 | 4 | 702461 | 2 | 501741 | 0,8190 |
| miR-1471 | 2 | 702463 | 4 | 501739 | 4 | 702461 | 2 | 501741 | 0,8190 |
| miR-148b* | 2 | 702463 | 4 | 501739 | 4 | 702461 | 2 | 501741 | 0,8190 |
| miR-1538 | 2 | 702463 | 4 | 501739 | 4 | 702461 | 2 | 501741 | 0,8190 |
| miR-1539 | 2 | 702463 | 4 | 501739 | 4 | 702461 | 2 | 501741 | 0,8190 |
| miR-182* | 2 | 702463 | 4 | 501739 | 4 | 702461 | 2 | 501741 | 0,8190 |
| miR-1825 | 2 | 702463 | 4 | 501739 | 4 | 702461 | 2 | 501741 | 0,8190 |
| miR-1827 | 2 | 702463 | 4 | 501739 | 4 | 702461 | 2 | 501741 | 0,8190 |
| miR-185* | 2 | 702463 | 4 | 501739 | 4 | 702461 | 2 | 501741 | 0,8190 |
| miR-18b* | 2 | 702463 | 4 | 501739 | 4 | 702461 | 2 | 501741 | 0,8190 |
| miR-1909 | 2 | 702463 | 4 | 501739 | 4 | 702461 | 2 | 501741 | 0,8190 |
| miR-1909* | 2 | 702463 | 4 | 501739 | 4 | 702461 | 2 | 501741 | 0,8190 |
| miR-1910 | 2 | 702463 | 4 | 501739 | 4 | 702461 | 2 | 501741 | 0,8190 |
| miR-1911 | 2 | 702463 | 4 | 501739 | 4 | 702461 | 2 | 501741 | 0,8190 |
| miR-1911* | 2 | 702463 | 4 | 501739 | 4 | 702461 | 2 | 501741 | 0,8190 |
| miR-1913 | 2 | 702463 | 4 | 501739 | 4 | 702461 | 2 | 501741 | 0,8190 |
| miR-1914 | 2 | 702463 | 4 | 501739 | 4 | 702461 | 2 | 501741 | 0,8190 |
| miR-1915 | 2 | 702463 | 4 | 501739 | 4 | 702461 | 2 | 501741 | 0,8190 |

| | | | | | | | | | |
|---|---|---|---|---|---|---|---|---|---|
| miR-1915* | 2 | 702463 | 4 | 501739 | 4 | 702461 | 2 | 501741 | 0,8190 |
| miR-196a* | 2 | 702463 | 4 | 501739 | 4 | 702461 | 2 | 501741 | 0,8190 |
| miR-196b* | 2 | 702463 | 4 | 501739 | 4 | 702461 | 2 | 501741 | 0,8190 |
| miR-1973 | 2 | 702463 | 4 | 501739 | 4 | 702461 | 2 | 501741 | 0,8190 |
| miR-1976 | 2 | 702463 | 4 | 501739 | 4 | 702461 | 2 | 501741 | 0,8190 |
| miR-198 | 2 | 702463 | 4 | 501739 | 4 | 702461 | 2 | 501741 | 0,8190 |
| miR-2052 | 2 | 702463 | 4 | 501739 | 4 | 702461 | 2 | 501741 | 0,8190 |
| miR-2053 | 2 | 702463 | 4 | 501739 | 4 | 702461 | 2 | 501741 | 0,8190 |
| miR-2054 | 2 | 702463 | 4 | 501739 | 4 | 702461 | 2 | 501741 | 0,8190 |
| miR-208b | 2 | 702463 | 4 | 501739 | 4 | 702461 | 2 | 501741 | 0,8190 |
| miR-2114* | 2 | 702463 | 4 | 501739 | 4 | 702461 | 2 | 501741 | 0,8190 |
| miR-2115 | 2 | 702463 | 4 | 501739 | 4 | 702461 | 2 | 501741 | 0,8190 |
| miR-2116* | 2 | 702463 | 4 | 501739 | 4 | 702461 | 2 | 501741 | 0,8190 |
| miR-2117 | 2 | 702463 | 4 | 501739 | 4 | 702461 | 2 | 501741 | 0,8190 |
| miR-216b | 2 | 702463 | 4 | 501739 | 4 | 702461 | 2 | 501741 | 0,8190 |
| miR-218-2* | 2 | 702463 | 4 | 501739 | 4 | 702461 | 2 | 501741 | 0,8190 |
| miR-219-1-3p | 2 | 702463 | 4 | 501739 | 4 | 702461 | 2 | 501741 | 0,8190 |
| miR-219-2-3p | 2 | 702463 | 4 | 501739 | 4 | 702461 | 2 | 501741 | 0,8190 |
| miR-224* | 2 | 702463 | 4 | 501739 | 4 | 702461 | 2 | 501741 | 0,8190 |
| miR-2276 | 2 | 702463 | 4 | 501739 | 4 | 702461 | 2 | 501741 | 0,8190 |
| miR-2277-3p | 2 | 702463 | 4 | 501739 | 4 | 702461 | 2 | 501741 | 0,8190 |
| miR-24-1* | 2 | 702463 | 4 | 501739 | 4 | 702461 | 2 | 501741 | 0,8190 |
| miR-2861 | 2 | 702463 | 4 | 501739 | 4 | 702461 | 2 | 501741 | 0,8190 |
| miR-2909 | 2 | 702463 | 4 | 501739 | 4 | 702461 | 2 | 501741 | 0,8190 |
| miR-297 | 2 | 702463 | 4 | 501739 | 4 | 702461 | 2 | 501741 | 0,8190 |
| miR-298 | 2 | 702463 | 4 | 501739 | 4 | 702461 | 2 | 501741 | 0,8190 |
| miR-300 | 2 | 702463 | 4 | 501739 | 4 | 702461 | 2 | 501741 | 0,8190 |
| miR-302e | 2 | 702463 | 4 | 501739 | 4 | 702461 | 2 | 501741 | 0,8190 |
| miR-302f | 2 | 702463 | 4 | 501739 | 4 | 702461 | 2 | 501741 | 0,8190 |
| miR-3074 | 2 | 702463 | 4 | 501739 | 4 | 702461 | 2 | 501741 | 0,8190 |

| | | | | | | | | | |
|---|---|---|---|---|---|---|---|---|---|
| miR-3115 | 2 | 702463 | 4 | 501739 | 4 | 702461 | 2 | 501741 | 0,8190 |
| miR-3116 | 2 | 702463 | 4 | 501739 | 4 | 702461 | 2 | 501741 | 0,8190 |
| miR-3119 | 2 | 702463 | 4 | 501739 | 4 | 702461 | 2 | 501741 | 0,8190 |
| miR-3121 | 2 | 702463 | 4 | 501739 | 4 | 702461 | 2 | 501741 | 0,8190 |
| miR-3122 | 2 | 702463 | 4 | 501739 | 4 | 702461 | 2 | 501741 | 0,8190 |
| miR-3123 | 2 | 702463 | 4 | 501739 | 4 | 702461 | 2 | 501741 | 0,8190 |
| miR-3124 | 2 | 702463 | 4 | 501739 | 4 | 702461 | 2 | 501741 | 0,8190 |
| miR-3126-3p | 2 | 702463 | 4 | 501739 | 4 | 702461 | 2 | 501741 | 0,8190 |
| miR-3126-5p | 2 | 702463 | 4 | 501739 | 4 | 702461 | 2 | 501741 | 0,8190 |
| miR-3127 | 2 | 702463 | 4 | 501739 | 4 | 702461 | 2 | 501741 | 0,8190 |
| miR-3128 | 2 | 702463 | 4 | 501739 | 4 | 702461 | 2 | 501741 | 0,8190 |
| miR-3131 | 2 | 702463 | 4 | 501739 | 4 | 702461 | 2 | 501741 | 0,8190 |
| miR-3132 | 2 | 702463 | 4 | 501739 | 4 | 702461 | 2 | 501741 | 0,8190 |
| miR-3134 | 2 | 702463 | 4 | 501739 | 4 | 702461 | 2 | 501741 | 0,8190 |
| miR-3135 | 2 | 702463 | 4 | 501739 | 4 | 702461 | 2 | 501741 | 0,8190 |
| miR-3136 | 2 | 702463 | 4 | 501739 | 4 | 702461 | 2 | 501741 | 0,8190 |
| miR-3137 | 2 | 702463 | 4 | 501739 | 4 | 702461 | 2 | 501741 | 0,8190 |
| miR-3138 | 2 | 702463 | 4 | 501739 | 4 | 702461 | 2 | 501741 | 0,8190 |
| miR-3139 | 2 | 702463 | 4 | 501739 | 4 | 702461 | 2 | 501741 | 0,8190 |
| miR-3140 | 2 | 702463 | 4 | 501739 | 4 | 702461 | 2 | 501741 | 0,8190 |
| miR-3141 | 2 | 702463 | 4 | 501739 | 4 | 702461 | 2 | 501741 | 0,8190 |
| miR-3142 | 2 | 702463 | 4 | 501739 | 4 | 702461 | 2 | 501741 | 0,8190 |
| miR-3144-3p | 2 | 702463 | 4 | 501739 | 4 | 702461 | 2 | 501741 | 0,8190 |
| miR-3144-5p | 2 | 702463 | 4 | 501739 | 4 | 702461 | 2 | 501741 | 0,8190 |
| miR-3145 | 2 | 702463 | 4 | 501739 | 4 | 702461 | 2 | 501741 | 0,8190 |
| miR-3146 | 2 | 702463 | 4 | 501739 | 4 | 702461 | 2 | 501741 | 0,8190 |
| miR-3147 | 2 | 702463 | 4 | 501739 | 4 | 702461 | 2 | 501741 | 0,8190 |
| miR-3149 | 2 | 702463 | 4 | 501739 | 4 | 702461 | 2 | 501741 | 0,8190 |
| miR-3150 | 2 | 702463 | 4 | 501739 | 4 | 702461 | 2 | 501741 | 0,8190 |
| miR-3150b | 2 | 702463 | 4 | 501739 | 4 | 702461 | 2 | 501741 | 0,8190 |

| | | | | | | | | | |
|---|---|---|---|---|---|---|---|---|---|
| miR-3152 | 2 | 702463 | 4 | 501739 | 4 | 702461 | 2 | 501741 | 0,8190 |
| miR-3153 | 2 | 702463 | 4 | 501739 | 4 | 702461 | 2 | 501741 | 0,8190 |
| miR-3155 | 2 | 702463 | 4 | 501739 | 4 | 702461 | 2 | 501741 | 0,8190 |
| miR-3157 | 2 | 702463 | 4 | 501739 | 4 | 702461 | 2 | 501741 | 0,8190 |
| miR-3159 | 2 | 702463 | 4 | 501739 | 4 | 702461 | 2 | 501741 | 0,8190 |
| miR-3161 | 2 | 702463 | 4 | 501739 | 4 | 702461 | 2 | 501741 | 0,8190 |
| miR-3162 | 2 | 702463 | 4 | 501739 | 4 | 702461 | 2 | 501741 | 0,8190 |
| miR-3164 | 2 | 702463 | 4 | 501739 | 4 | 702461 | 2 | 501741 | 0,8190 |
| miR-3165 | 2 | 702463 | 4 | 501739 | 4 | 702461 | 2 | 501741 | 0,8190 |
| miR-3167 | 2 | 702463 | 4 | 501739 | 4 | 702461 | 2 | 501741 | 0,8190 |
| miR-3168 | 2 | 702463 | 4 | 501739 | 4 | 702461 | 2 | 501741 | 0,8190 |
| miR-3169 | 2 | 702463 | 4 | 501739 | 4 | 702461 | 2 | 501741 | 0,8190 |
| miR-3170 | 2 | 702463 | 4 | 501739 | 4 | 702461 | 2 | 501741 | 0,8190 |
| miR-3171 | 2 | 702463 | 4 | 501739 | 4 | 702461 | 2 | 501741 | 0,8190 |
| miR-3173 | 2 | 702463 | 4 | 501739 | 4 | 702461 | 2 | 501741 | 0,8190 |
| miR-3174 | 2 | 702463 | 4 | 501739 | 4 | 702461 | 2 | 501741 | 0,8190 |
| miR-3176 | 2 | 702463 | 4 | 501739 | 4 | 702461 | 2 | 501741 | 0,8190 |
| miR-3178 | 2 | 702463 | 4 | 501739 | 4 | 702461 | 2 | 501741 | 0,8190 |
| miR-3181 | 2 | 702463 | 4 | 501739 | 4 | 702461 | 2 | 501741 | 0,8190 |
| miR-3182 | 2 | 702463 | 4 | 501739 | 4 | 702461 | 2 | 501741 | 0,8190 |
| miR-3183 | 2 | 702463 | 4 | 501739 | 4 | 702461 | 2 | 501741 | 0,8190 |
| miR-3185 | 2 | 702463 | 4 | 501739 | 4 | 702461 | 2 | 501741 | 0,8190 |
| miR-3186-3p | 2 | 702463 | 4 | 501739 | 4 | 702461 | 2 | 501741 | 0,8190 |
| miR-3186-5p | 2 | 702463 | 4 | 501739 | 4 | 702461 | 2 | 501741 | 0,8190 |
| miR-3187 | 2 | 702463 | 4 | 501739 | 4 | 702461 | 2 | 501741 | 0,8190 |
| miR-3188 | 2 | 702463 | 4 | 501739 | 4 | 702461 | 2 | 501741 | 0,8190 |
| miR-3189 | 2 | 702463 | 4 | 501739 | 4 | 702461 | 2 | 501741 | 0,8190 |
| miR-3191 | 2 | 702463 | 4 | 501739 | 4 | 702461 | 2 | 501741 | 0,8190 |
| miR-3193 | 2 | 702463 | 4 | 501739 | 4 | 702461 | 2 | 501741 | 0,8190 |
| miR-3194 | 2 | 702463 | 4 | 501739 | 4 | 702461 | 2 | 501741 | 0,8190 |

| | | | | | | | | | |
|---|---|---|---|---|---|---|---|---|---|
| miR-3195 | 2 | 702463 | 4 | 501739 | 4 | 702461 | 2 | 501741 | 0,8190 |
| miR-3196 | 2 | 702463 | 4 | 501739 | 4 | 702461 | 2 | 501741 | 0,8190 |
| miR-3197 | 2 | 702463 | 4 | 501739 | 4 | 702461 | 2 | 501741 | 0,8190 |
| miR-3198 | 2 | 702463 | 4 | 501739 | 4 | 702461 | 2 | 501741 | 0,8190 |
| miR-3201 | 2 | 702463 | 4 | 501739 | 4 | 702461 | 2 | 501741 | 0,8190 |
| miR-3202 | 2 | 702463 | 4 | 501739 | 4 | 702461 | 2 | 501741 | 0,8190 |
| miR-320d | 2 | 702463 | 4 | 501739 | 4 | 702461 | 2 | 501741 | 0,8190 |
| miR-320e | 2 | 702463 | 4 | 501739 | 4 | 702461 | 2 | 501741 | 0,8190 |
| miR-323-5p | 2 | 702463 | 4 | 501739 | 4 | 702461 | 2 | 501741 | 0,8190 |
| miR-325 | 2 | 702463 | 4 | 501739 | 4 | 702461 | 2 | 501741 | 0,8190 |
| miR-346 | 2 | 702463 | 4 | 501739 | 4 | 702461 | 2 | 501741 | 0,8190 |
| miR-3607-3p | 2 | 702463 | 4 | 501739 | 4 | 702461 | 2 | 501741 | 0,8190 |
| miR-3610 | 2 | 702463 | 4 | 501739 | 4 | 702461 | 2 | 501741 | 0,8190 |
| miR-3616-5p | 2 | 702463 | 4 | 501739 | 4 | 702461 | 2 | 501741 | 0,8190 |
| miR-3617 | 2 | 702463 | 4 | 501739 | 4 | 702461 | 2 | 501741 | 0,8190 |
| miR-3618 | 2 | 702463 | 4 | 501739 | 4 | 702461 | 2 | 501741 | 0,8190 |
| miR-3619 | 2 | 702463 | 4 | 501739 | 4 | 702461 | 2 | 501741 | 0,8190 |
| miR-3646 | 2 | 702463 | 4 | 501739 | 4 | 702461 | 2 | 501741 | 0,8190 |
| miR-3647-3p | 2 | 702463 | 4 | 501739 | 4 | 702461 | 2 | 501741 | 0,8190 |
| miR-3648 | 2 | 702463 | 4 | 501739 | 4 | 702461 | 2 | 501741 | 0,8190 |
| miR-3649 | 2 | 702463 | 4 | 501739 | 4 | 702461 | 2 | 501741 | 0,8190 |
| miR-3650 | 2 | 702463 | 4 | 501739 | 4 | 702461 | 2 | 501741 | 0,8190 |
| miR-3651 | 2 | 702463 | 4 | 501739 | 4 | 702461 | 2 | 501741 | 0,8190 |
| miR-3652 | 2 | 702463 | 4 | 501739 | 4 | 702461 | 2 | 501741 | 0,8190 |
| miR-3653 | 2 | 702463 | 4 | 501739 | 4 | 702461 | 2 | 501741 | 0,8190 |
| miR-3654 | 2 | 702463 | 4 | 501739 | 4 | 702461 | 2 | 501741 | 0,8190 |
| miR-3655 | 2 | 702463 | 4 | 501739 | 4 | 702461 | 2 | 501741 | 0,8190 |
| miR-3656 | 2 | 702463 | 4 | 501739 | 4 | 702461 | 2 | 501741 | 0,8190 |
| miR-3657 | 2 | 702463 | 4 | 501739 | 4 | 702461 | 2 | 501741 | 0,8190 |
| miR-3658 | 2 | 702463 | 4 | 501739 | 4 | 702461 | 2 | 501741 | 0,8190 |

| | | | | | | | | | |
|---|---|---|---|---|---|---|---|---|---|
| miR-3659 | 2 | 702463 | 4 | 501739 | 4 | 702461 | 2 | 501741 | 0,8190 |
| miR-3662 | 2 | 702463 | 4 | 501739 | 4 | 702461 | 2 | 501741 | 0,8190 |
| miR-3663-5p | 2 | 702463 | 4 | 501739 | 4 | 702461 | 2 | 501741 | 0,8190 |
| miR-3664 | 2 | 702463 | 4 | 501739 | 4 | 702461 | 2 | 501741 | 0,8190 |
| miR-3665 | 2 | 702463 | 4 | 501739 | 4 | 702461 | 2 | 501741 | 0,8190 |
| miR-3666 | 2 | 702463 | 4 | 501739 | 4 | 702461 | 2 | 501741 | 0,8190 |
| miR-3668 | 2 | 702463 | 4 | 501739 | 4 | 702461 | 2 | 501741 | 0,8190 |
| miR-3669 | 2 | 702463 | 4 | 501739 | 4 | 702461 | 2 | 501741 | 0,8190 |
| miR-3670 | 2 | 702463 | 4 | 501739 | 4 | 702461 | 2 | 501741 | 0,8190 |
| miR-3671 | 2 | 702463 | 4 | 501739 | 4 | 702461 | 2 | 501741 | 0,8190 |
| miR-3672 | 2 | 702463 | 4 | 501739 | 4 | 702461 | 2 | 501741 | 0,8190 |
| miR-3673 | 2 | 702463 | 4 | 501739 | 4 | 702461 | 2 | 501741 | 0,8190 |
| miR-3674 | 2 | 702463 | 4 | 501739 | 4 | 702461 | 2 | 501741 | 0,8190 |
| miR-3675-3p | 2 | 702463 | 4 | 501739 | 4 | 702461 | 2 | 501741 | 0,8190 |
| miR-3675-5p | 2 | 702463 | 4 | 501739 | 4 | 702461 | 2 | 501741 | 0,8190 |
| miR-3677 | 2 | 702463 | 4 | 501739 | 4 | 702461 | 2 | 501741 | 0,8190 |
| miR-3678-3p | 2 | 702463 | 4 | 501739 | 4 | 702461 | 2 | 501741 | 0,8190 |
| miR-3678-5p | 2 | 702463 | 4 | 501739 | 4 | 702461 | 2 | 501741 | 0,8190 |
| miR-3679-3p | 2 | 702463 | 4 | 501739 | 4 | 702461 | 2 | 501741 | 0,8190 |
| miR-3680 | 2 | 702463 | 4 | 501739 | 4 | 702461 | 2 | 501741 | 0,8190 |
| miR-3680* | 2 | 702463 | 4 | 501739 | 4 | 702461 | 2 | 501741 | 0,8190 |
| miR-3681 | 2 | 702463 | 4 | 501739 | 4 | 702461 | 2 | 501741 | 0,8190 |
| miR-3681* | 2 | 702463 | 4 | 501739 | 4 | 702461 | 2 | 501741 | 0,8190 |
| miR-3682 | 2 | 702463 | 4 | 501739 | 4 | 702461 | 2 | 501741 | 0,8190 |
| miR-3683 | 2 | 702463 | 4 | 501739 | 4 | 702461 | 2 | 501741 | 0,8190 |
| miR-3685 | 2 | 702463 | 4 | 501739 | 4 | 702461 | 2 | 501741 | 0,8190 |
| miR-3686 | 2 | 702463 | 4 | 501739 | 4 | 702461 | 2 | 501741 | 0,8190 |
| miR-3689a-3p | 2 | 702463 | 4 | 501739 | 4 | 702461 | 2 | 501741 | 0,8190 |
| miR-3689b* | 2 | 702463 | 4 | 501739 | 4 | 702461 | 2 | 501741 | 0,8190 |
| miR-3690 | 2 | 702463 | 4 | 501739 | 4 | 702461 | 2 | 501741 | 0,8190 |

| | | | | | | | | | |
|---|---|---|---|---|---|---|---|---|---|
| miR-3691 | 2 | 702463 | 4 | 501739 | 4 | 702461 | 2 | 501741 | 0,8190 |
| miR-3692 | 2 | 702463 | 4 | 501739 | 4 | 702461 | 2 | 501741 | 0,8190 |
| miR-3713 | 2 | 702463 | 4 | 501739 | 4 | 702461 | 2 | 501741 | 0,8190 |
| miR-3714 | 2 | 702463 | 4 | 501739 | 4 | 702461 | 2 | 501741 | 0,8190 |
| miR-378b | 2 | 702463 | 4 | 501739 | 4 | 702461 | 2 | 501741 | 0,8190 |
| miR-384 | 2 | 702463 | 4 | 501739 | 4 | 702461 | 2 | 501741 | 0,8190 |
| miR-3907 | 2 | 702463 | 4 | 501739 | 4 | 702461 | 2 | 501741 | 0,8190 |
| miR-3908 | 2 | 702463 | 4 | 501739 | 4 | 702461 | 2 | 501741 | 0,8190 |
| miR-3909 | 2 | 702463 | 4 | 501739 | 4 | 702461 | 2 | 501741 | 0,8190 |
| miR-3911 | 2 | 702463 | 4 | 501739 | 4 | 702461 | 2 | 501741 | 0,8190 |
| miR-3912 | 2 | 702463 | 4 | 501739 | 4 | 702461 | 2 | 501741 | 0,8190 |
| miR-3914 | 2 | 702463 | 4 | 501739 | 4 | 702461 | 2 | 501741 | 0,8190 |
| miR-3917 | 2 | 702463 | 4 | 501739 | 4 | 702461 | 2 | 501741 | 0,8190 |
| miR-3918 | 2 | 702463 | 4 | 501739 | 4 | 702461 | 2 | 501741 | 0,8190 |
| miR-3919 | 2 | 702463 | 4 | 501739 | 4 | 702461 | 2 | 501741 | 0,8190 |
| miR-3920 | 2 | 702463 | 4 | 501739 | 4 | 702461 | 2 | 501741 | 0,8190 |
| miR-3921 | 2 | 702463 | 4 | 501739 | 4 | 702461 | 2 | 501741 | 0,8190 |
| miR-3922 | 2 | 702463 | 4 | 501739 | 4 | 702461 | 2 | 501741 | 0,8190 |
| miR-3923 | 2 | 702463 | 4 | 501739 | 4 | 702461 | 2 | 501741 | 0,8190 |
| miR-3924 | 2 | 702463 | 4 | 501739 | 4 | 702461 | 2 | 501741 | 0,8190 |
| miR-3925 | 2 | 702463 | 4 | 501739 | 4 | 702461 | 2 | 501741 | 0,8190 |
| miR-3926 | 2 | 702463 | 4 | 501739 | 4 | 702461 | 2 | 501741 | 0,8190 |
| miR-3927 | 2 | 702463 | 4 | 501739 | 4 | 702461 | 2 | 501741 | 0,8190 |
| miR-3928 | 2 | 702463 | 4 | 501739 | 4 | 702461 | 2 | 501741 | 0,8190 |
| miR-3929 | 2 | 702463 | 4 | 501739 | 4 | 702461 | 2 | 501741 | 0,8190 |
| miR-3934 | 2 | 702463 | 4 | 501739 | 4 | 702461 | 2 | 501741 | 0,8190 |
| miR-3935 | 2 | 702463 | 4 | 501739 | 4 | 702461 | 2 | 501741 | 0,8190 |
| miR-3937 | 2 | 702463 | 4 | 501739 | 4 | 702461 | 2 | 501741 | 0,8190 |
| miR-3938 | 2 | 702463 | 4 | 501739 | 4 | 702461 | 2 | 501741 | 0,8190 |
| miR-3939 | 2 | 702463 | 4 | 501739 | 4 | 702461 | 2 | 501741 | 0,8190 |

| | | | | | | | | | |
|---|---|---|---|---|---|---|---|---|---|
| miR-3941 | 2 | 702463 | 4 | 501739 | 4 | 702461 | 2 | 501741 | 0,8190 |
| miR-3942 | 2 | 702463 | 4 | 501739 | 4 | 702461 | 2 | 501741 | 0,8190 |
| miR-3943 | 2 | 702463 | 4 | 501739 | 4 | 702461 | 2 | 501741 | 0,8190 |
| miR-3945 | 2 | 702463 | 4 | 501739 | 4 | 702461 | 2 | 501741 | 0,8190 |
| miR-4251 | 2 | 702463 | 4 | 501739 | 4 | 702461 | 2 | 501741 | 0,8190 |
| miR-4252 | 2 | 702463 | 4 | 501739 | 4 | 702461 | 2 | 501741 | 0,8190 |
| miR-4253 | 2 | 702463 | 4 | 501739 | 4 | 702461 | 2 | 501741 | 0,8190 |
| miR-4254 | 2 | 702463 | 4 | 501739 | 4 | 702461 | 2 | 501741 | 0,8190 |
| miR-4255 | 2 | 702463 | 4 | 501739 | 4 | 702461 | 2 | 501741 | 0,8190 |
| miR-4256 | 2 | 702463 | 4 | 501739 | 4 | 702461 | 2 | 501741 | 0,8190 |
| miR-4257 | 2 | 702463 | 4 | 501739 | 4 | 702461 | 2 | 501741 | 0,8190 |
| miR-4258 | 2 | 702463 | 4 | 501739 | 4 | 702461 | 2 | 501741 | 0,8190 |
| miR-4259 | 2 | 702463 | 4 | 501739 | 4 | 702461 | 2 | 501741 | 0,8190 |
| miR-4260 | 2 | 702463 | 4 | 501739 | 4 | 702461 | 2 | 501741 | 0,8190 |
| miR-4261 | 2 | 702463 | 4 | 501739 | 4 | 702461 | 2 | 501741 | 0,8190 |
| miR-4262 | 2 | 702463 | 4 | 501739 | 4 | 702461 | 2 | 501741 | 0,8190 |
| miR-4263 | 2 | 702463 | 4 | 501739 | 4 | 702461 | 2 | 501741 | 0,8190 |
| miR-4264 | 2 | 702463 | 4 | 501739 | 4 | 702461 | 2 | 501741 | 0,8190 |
| miR-4265 | 2 | 702463 | 4 | 501739 | 4 | 702461 | 2 | 501741 | 0,8190 |
| miR-4266 | 2 | 702463 | 4 | 501739 | 4 | 702461 | 2 | 501741 | 0,8190 |
| miR-4267 | 2 | 702463 | 4 | 501739 | 4 | 702461 | 2 | 501741 | 0,8190 |
| miR-4268 | 2 | 702463 | 4 | 501739 | 4 | 702461 | 2 | 501741 | 0,8190 |
| miR-4269 | 2 | 702463 | 4 | 501739 | 4 | 702461 | 2 | 501741 | 0,8190 |
| miR-4270 | 2 | 702463 | 4 | 501739 | 4 | 702461 | 2 | 501741 | 0,8190 |
| miR-4271 | 2 | 702463 | 4 | 501739 | 4 | 702461 | 2 | 501741 | 0,8190 |
| miR-4272 | 2 | 702463 | 4 | 501739 | 4 | 702461 | 2 | 501741 | 0,8190 |
| miR-4273 | 2 | 702463 | 4 | 501739 | 4 | 702461 | 2 | 501741 | 0,8190 |
| miR-4274 | 2 | 702463 | 4 | 501739 | 4 | 702461 | 2 | 501741 | 0,8190 |
| miR-4275 | 2 | 702463 | 4 | 501739 | 4 | 702461 | 2 | 501741 | 0,8190 |
| miR-4276 | 2 | 702463 | 4 | 501739 | 4 | 702461 | 2 | 501741 | 0,8190 |

| | | | | | | | | | |
|---|---|---|---|---|---|---|---|---|---|
| miR-4277 | 2 | 702463 | 4 | 501739 | 4 | 702461 | 2 | 501741 | 0,8190 |
| miR-4278 | 2 | 702463 | 4 | 501739 | 4 | 702461 | 2 | 501741 | 0,8190 |
| miR-4279 | 2 | 702463 | 4 | 501739 | 4 | 702461 | 2 | 501741 | 0,8190 |
| miR-4280 | 2 | 702463 | 4 | 501739 | 4 | 702461 | 2 | 501741 | 0,8190 |
| miR-4281 | 2 | 702463 | 4 | 501739 | 4 | 702461 | 2 | 501741 | 0,8190 |
| miR-4282 | 2 | 702463 | 4 | 501739 | 4 | 702461 | 2 | 501741 | 0,8190 |
| miR-4283 | 2 | 702463 | 4 | 501739 | 4 | 702461 | 2 | 501741 | 0,8190 |
| miR-4284 | 2 | 702463 | 4 | 501739 | 4 | 702461 | 2 | 501741 | 0,8190 |
| miR-4285 | 2 | 702463 | 4 | 501739 | 4 | 702461 | 2 | 501741 | 0,8190 |
| miR-4286 | 2 | 702463 | 4 | 501739 | 4 | 702461 | 2 | 501741 | 0,8190 |
| miR-4287 | 2 | 702463 | 4 | 501739 | 4 | 702461 | 2 | 501741 | 0,8190 |
| miR-4288 | 2 | 702463 | 4 | 501739 | 4 | 702461 | 2 | 501741 | 0,8190 |
| miR-4289 | 2 | 702463 | 4 | 501739 | 4 | 702461 | 2 | 501741 | 0,8190 |
| miR-4290 | 2 | 702463 | 4 | 501739 | 4 | 702461 | 2 | 501741 | 0,8190 |
| miR-4291 | 2 | 702463 | 4 | 501739 | 4 | 702461 | 2 | 501741 | 0,8190 |
| miR-4292 | 2 | 702463 | 4 | 501739 | 4 | 702461 | 2 | 501741 | 0,8190 |
| miR-4293 | 2 | 702463 | 4 | 501739 | 4 | 702461 | 2 | 501741 | 0,8190 |
| miR-4294 | 2 | 702463 | 4 | 501739 | 4 | 702461 | 2 | 501741 | 0,8190 |
| miR-4295 | 2 | 702463 | 4 | 501739 | 4 | 702461 | 2 | 501741 | 0,8190 |
| miR-4296 | 2 | 702463 | 4 | 501739 | 4 | 702461 | 2 | 501741 | 0,8190 |
| miR-4297 | 2 | 702463 | 4 | 501739 | 4 | 702461 | 2 | 501741 | 0,8190 |
| miR-4298 | 2 | 702463 | 4 | 501739 | 4 | 702461 | 2 | 501741 | 0,8190 |
| miR-4299 | 2 | 702463 | 4 | 501739 | 4 | 702461 | 2 | 501741 | 0,8190 |
| miR-4300 | 2 | 702463 | 4 | 501739 | 4 | 702461 | 2 | 501741 | 0,8190 |
| miR-4301 | 2 | 702463 | 4 | 501739 | 4 | 702461 | 2 | 501741 | 0,8190 |
| miR-4302 | 2 | 702463 | 4 | 501739 | 4 | 702461 | 2 | 501741 | 0,8190 |
| miR-4303 | 2 | 702463 | 4 | 501739 | 4 | 702461 | 2 | 501741 | 0,8190 |
| miR-4304 | 2 | 702463 | 4 | 501739 | 4 | 702461 | 2 | 501741 | 0,8190 |
| miR-4305 | 2 | 702463 | 4 | 501739 | 4 | 702461 | 2 | 501741 | 0,8190 |
| miR-4306 | 2 | 702463 | 4 | 501739 | 4 | 702461 | 2 | 501741 | 0,8190 |

| | | | | | | | | | |
|---|---|---|---|---|---|---|---|---|---|
| miR-4307 | 2 | 702463 | 4 | 501739 | 4 | 702461 | 2 | 501741 | 0,8190 |
| miR-4308 | 2 | 702463 | 4 | 501739 | 4 | 702461 | 2 | 501741 | 0,8190 |
| miR-4309 | 2 | 702463 | 4 | 501739 | 4 | 702461 | 2 | 501741 | 0,8190 |
| miR-4310 | 2 | 702463 | 4 | 501739 | 4 | 702461 | 2 | 501741 | 0,8190 |
| miR-4311 | 2 | 702463 | 4 | 501739 | 4 | 702461 | 2 | 501741 | 0,8190 |
| miR-4312 | 2 | 702463 | 4 | 501739 | 4 | 702461 | 2 | 501741 | 0,8190 |
| miR-4313 | 2 | 702463 | 4 | 501739 | 4 | 702461 | 2 | 501741 | 0,8190 |
| miR-4314 | 2 | 702463 | 4 | 501739 | 4 | 702461 | 2 | 501741 | 0,8190 |
| miR-4315 | 2 | 702463 | 4 | 501739 | 4 | 702461 | 2 | 501741 | 0,8190 |
| miR-4316 | 2 | 702463 | 4 | 501739 | 4 | 702461 | 2 | 501741 | 0,8190 |
| miR-4317 | 2 | 702463 | 4 | 501739 | 4 | 702461 | 2 | 501741 | 0,8190 |
| miR-4318 | 2 | 702463 | 4 | 501739 | 4 | 702461 | 2 | 501741 | 0,8190 |
| miR-4319 | 2 | 702463 | 4 | 501739 | 4 | 702461 | 2 | 501741 | 0,8190 |
| miR-4320 | 2 | 702463 | 4 | 501739 | 4 | 702461 | 2 | 501741 | 0,8190 |
| miR-4321 | 2 | 702463 | 4 | 501739 | 4 | 702461 | 2 | 501741 | 0,8190 |
| miR-4322 | 2 | 702463 | 4 | 501739 | 4 | 702461 | 2 | 501741 | 0,8190 |
| miR-4323 | 2 | 702463 | 4 | 501739 | 4 | 702461 | 2 | 501741 | 0,8190 |
| miR-4324 | 2 | 702463 | 4 | 501739 | 4 | 702461 | 2 | 501741 | 0,8190 |
| miR-4325 | 2 | 702463 | 4 | 501739 | 4 | 702461 | 2 | 501741 | 0,8190 |
| miR-4327 | 2 | 702463 | 4 | 501739 | 4 | 702461 | 2 | 501741 | 0,8190 |
| miR-4328 | 2 | 702463 | 4 | 501739 | 4 | 702461 | 2 | 501741 | 0,8190 |
| miR-4329 | 2 | 702463 | 4 | 501739 | 4 | 702461 | 2 | 501741 | 0,8190 |
| miR-4330 | 2 | 702463 | 4 | 501739 | 4 | 702461 | 2 | 501741 | 0,8190 |
| miR-448 | 2 | 702463 | 4 | 501739 | 4 | 702461 | 2 | 501741 | 0,8190 |
| miR-449b | 2 | 702463 | 4 | 501739 | 4 | 702461 | 2 | 501741 | 0,8190 |
| miR-449b* | 2 | 702463 | 4 | 501739 | 4 | 702461 | 2 | 501741 | 0,8190 |
| miR-449c | 2 | 702463 | 4 | 501739 | 4 | 702461 | 2 | 501741 | 0,8190 |
| miR-449c* | 2 | 702463 | 4 | 501739 | 4 | 702461 | 2 | 501741 | 0,8190 |
| miR-452* | 2 | 702463 | 4 | 501739 | 4 | 702461 | 2 | 501741 | 0,8190 |
| miR-490-3p | 2 | 702463 | 4 | 501739 | 4 | 702461 | 2 | 501741 | 0,8190 |

| | | | | | | | | | |
|---|---|---|---|---|---|---|---|---|---|
| miR-490-5p | 2 | 702463 | 4 | 501739 | 4 | 702461 | 2 | 501741 | 0,8190 |
| miR-492 | 2 | 702463 | 4 | 501739 | 4 | 702461 | 2 | 501741 | 0,8190 |
| miR-513a-5p | 2 | 702463 | 4 | 501739 | 4 | 702461 | 2 | 501741 | 0,8190 |
| miR-541 | 2 | 702463 | 4 | 501739 | 4 | 702461 | 2 | 501741 | 0,8190 |
| miR-544b | 2 | 702463 | 4 | 501739 | 4 | 702461 | 2 | 501741 | 0,8190 |
| miR-548f | 2 | 702463 | 4 | 501739 | 4 | 702461 | 2 | 501741 | 0,8190 |
| miR-548h | 2 | 702463 | 4 | 501739 | 4 | 702461 | 2 | 501741 | 0,8190 |
| miR-548l | 2 | 702463 | 4 | 501739 | 4 | 702461 | 2 | 501741 | 0,8190 |
| miR-548s | 2 | 702463 | 4 | 501739 | 4 | 702461 | 2 | 501741 | 0,8190 |
| miR-548t | 2 | 702463 | 4 | 501739 | 4 | 702461 | 2 | 501741 | 0,8190 |
| miR-548u | 2 | 702463 | 4 | 501739 | 4 | 702461 | 2 | 501741 | 0,8190 |
| miR-548v | 2 | 702463 | 4 | 501739 | 4 | 702461 | 2 | 501741 | 0,8190 |
| miR-550b | 2 | 702463 | 4 | 501739 | 4 | 702461 | 2 | 501741 | 0,8190 |
| miR-551b* | 2 | 702463 | 4 | 501739 | 4 | 702461 | 2 | 501741 | 0,8190 |
| miR-553 | 2 | 702463 | 4 | 501739 | 4 | 702461 | 2 | 501741 | 0,8190 |
| miR-554 | 2 | 702463 | 4 | 501739 | 4 | 702461 | 2 | 501741 | 0,8190 |
| miR-555 | 2 | 702463 | 4 | 501739 | 4 | 702461 | 2 | 501741 | 0,8190 |
| miR-556-3p | 2 | 702463 | 4 | 501739 | 4 | 702461 | 2 | 501741 | 0,8190 |
| miR-557 | 2 | 702463 | 4 | 501739 | 4 | 702461 | 2 | 501741 | 0,8190 |
| miR-558 | 2 | 702463 | 4 | 501739 | 4 | 702461 | 2 | 501741 | 0,8190 |
| miR-562 | 2 | 702463 | 4 | 501739 | 4 | 702461 | 2 | 501741 | 0,8190 |
| miR-563 | 2 | 702463 | 4 | 501739 | 4 | 702461 | 2 | 501741 | 0,8190 |
| miR-564 | 2 | 702463 | 4 | 501739 | 4 | 702461 | 2 | 501741 | 0,8190 |
| miR-566 | 2 | 702463 | 4 | 501739 | 4 | 702461 | 2 | 501741 | 0,8190 |
| miR-567 | 2 | 702463 | 4 | 501739 | 4 | 702461 | 2 | 501741 | 0,8190 |
| miR-568 | 2 | 702463 | 4 | 501739 | 4 | 702461 | 2 | 501741 | 0,8190 |
| miR-569 | 2 | 702463 | 4 | 501739 | 4 | 702461 | 2 | 501741 | 0,8190 |
| miR-571 | 2 | 702463 | 4 | 501739 | 4 | 702461 | 2 | 501741 | 0,8190 |
| miR-572 | 2 | 702463 | 4 | 501739 | 4 | 702461 | 2 | 501741 | 0,8190 |
| miR-573 | 2 | 702463 | 4 | 501739 | 4 | 702461 | 2 | 501741 | 0,8190 |

| | | | | | | | | | |
|---|---|---|---|---|---|---|---|---|---|
| miR-575 | 2 | 702463 | 4 | 501739 | 4 | 702461 | 2 | 501741 | 0,8190 |
| miR-577 | 2 | 702463 | 4 | 501739 | 4 | 702461 | 2 | 501741 | 0,8190 |
| miR-578 | 2 | 702463 | 4 | 501739 | 4 | 702461 | 2 | 501741 | 0,8190 |
| miR-580 | 2 | 702463 | 4 | 501739 | 4 | 702461 | 2 | 501741 | 0,8190 |
| miR-581 | 2 | 702463 | 4 | 501739 | 4 | 702461 | 2 | 501741 | 0,8190 |
| miR-583 | 2 | 702463 | 4 | 501739 | 4 | 702461 | 2 | 501741 | 0,8190 |
| miR-585 | 2 | 702463 | 4 | 501739 | 4 | 702461 | 2 | 501741 | 0,8190 |
| miR-586 | 2 | 702463 | 4 | 501739 | 4 | 702461 | 2 | 501741 | 0,8190 |
| miR-587 | 2 | 702463 | 4 | 501739 | 4 | 702461 | 2 | 501741 | 0,8190 |
| miR-588 | 2 | 702463 | 4 | 501739 | 4 | 702461 | 2 | 501741 | 0,8190 |
| miR-591 | 2 | 702463 | 4 | 501739 | 4 | 702461 | 2 | 501741 | 0,8190 |
| miR-593 | 2 | 702463 | 4 | 501739 | 4 | 702461 | 2 | 501741 | 0,8190 |
| miR-593* | 2 | 702463 | 4 | 501739 | 4 | 702461 | 2 | 501741 | 0,8190 |
| miR-595 | 2 | 702463 | 4 | 501739 | 4 | 702461 | 2 | 501741 | 0,8190 |
| miR-596 | 2 | 702463 | 4 | 501739 | 4 | 702461 | 2 | 501741 | 0,8190 |
| miR-597 | 2 | 702463 | 4 | 501739 | 4 | 702461 | 2 | 501741 | 0,8190 |
| miR-599 | 2 | 702463 | 4 | 501739 | 4 | 702461 | 2 | 501741 | 0,8190 |
| miR-600 | 2 | 702463 | 4 | 501739 | 4 | 702461 | 2 | 501741 | 0,8190 |
| miR-601 | 2 | 702463 | 4 | 501739 | 4 | 702461 | 2 | 501741 | 0,8190 |
| miR-602 | 2 | 702463 | 4 | 501739 | 4 | 702461 | 2 | 501741 | 0,8190 |
| miR-603 | 2 | 702463 | 4 | 501739 | 4 | 702461 | 2 | 501741 | 0,8190 |
| miR-604 | 2 | 702463 | 4 | 501739 | 4 | 702461 | 2 | 501741 | 0,8190 |
| miR-605 | 2 | 702463 | 4 | 501739 | 4 | 702461 | 2 | 501741 | 0,8190 |
| miR-606 | 2 | 702463 | 4 | 501739 | 4 | 702461 | 2 | 501741 | 0,8190 |
| miR-607 | 2 | 702463 | 4 | 501739 | 4 | 702461 | 2 | 501741 | 0,8190 |
| miR-608 | 2 | 702463 | 4 | 501739 | 4 | 702461 | 2 | 501741 | 0,8190 |
| miR-609 | 2 | 702463 | 4 | 501739 | 4 | 702461 | 2 | 501741 | 0,8190 |
| miR-610 | 2 | 702463 | 4 | 501739 | 4 | 702461 | 2 | 501741 | 0,8190 |
| miR-611 | 2 | 702463 | 4 | 501739 | 4 | 702461 | 2 | 501741 | 0,8190 |
| miR-612 | 2 | 702463 | 4 | 501739 | 4 | 702461 | 2 | 501741 | 0,8190 |

| | | | | | | | | | |
|---|---|---|---|---|---|---|---|---|---|
| miR-613 | 2 | 702463 | 4 | 501739 | 4 | 702461 | 2 | 501741 | 0,8190 |
| miR-614 | 2 | 702463 | 4 | 501739 | 4 | 702461 | 2 | 501741 | 0,8190 |
| miR-615-5p | 2 | 702463 | 4 | 501739 | 4 | 702461 | 2 | 501741 | 0,8190 |
| miR-617 | 2 | 702463 | 4 | 501739 | 4 | 702461 | 2 | 501741 | 0,8190 |
| miR-618 | 2 | 702463 | 4 | 501739 | 4 | 702461 | 2 | 501741 | 0,8190 |
| miR-619 | 2 | 702463 | 4 | 501739 | 4 | 702461 | 2 | 501741 | 0,8190 |
| miR-620 | 2 | 702463 | 4 | 501739 | 4 | 702461 | 2 | 501741 | 0,8190 |
| miR-621 | 2 | 702463 | 4 | 501739 | 4 | 702461 | 2 | 501741 | 0,8190 |
| miR-622 | 2 | 702463 | 4 | 501739 | 4 | 702461 | 2 | 501741 | 0,8190 |
| miR-623 | 2 | 702463 | 4 | 501739 | 4 | 702461 | 2 | 501741 | 0,8190 |
| miR-626 | 2 | 702463 | 4 | 501739 | 4 | 702461 | 2 | 501741 | 0,8190 |
| miR-630 | 2 | 702463 | 4 | 501739 | 4 | 702461 | 2 | 501741 | 0,8190 |
| miR-631 | 2 | 702463 | 4 | 501739 | 4 | 702461 | 2 | 501741 | 0,8190 |
| miR-632 | 2 | 702463 | 4 | 501739 | 4 | 702461 | 2 | 501741 | 0,8190 |
| miR-633 | 2 | 702463 | 4 | 501739 | 4 | 702461 | 2 | 501741 | 0,8190 |
| miR-634 | 2 | 702463 | 4 | 501739 | 4 | 702461 | 2 | 501741 | 0,8190 |
| miR-635 | 2 | 702463 | 4 | 501739 | 4 | 702461 | 2 | 501741 | 0,8190 |
| miR-637 | 2 | 702463 | 4 | 501739 | 4 | 702461 | 2 | 501741 | 0,8190 |
| miR-638 | 2 | 702463 | 4 | 501739 | 4 | 702461 | 2 | 501741 | 0,8190 |
| miR-639 | 2 | 702463 | 4 | 501739 | 4 | 702461 | 2 | 501741 | 0,8190 |
| miR-640 | 2 | 702463 | 4 | 501739 | 4 | 702461 | 2 | 501741 | 0,8190 |
| miR-644 | 2 | 702463 | 4 | 501739 | 4 | 702461 | 2 | 501741 | 0,8190 |
| miR-645 | 2 | 702463 | 4 | 501739 | 4 | 702461 | 2 | 501741 | 0,8190 |
| miR-646 | 2 | 702463 | 4 | 501739 | 4 | 702461 | 2 | 501741 | 0,8190 |
| miR-647 | 2 | 702463 | 4 | 501739 | 4 | 702461 | 2 | 501741 | 0,8190 |
| miR-648 | 2 | 702463 | 4 | 501739 | 4 | 702461 | 2 | 501741 | 0,8190 |
| miR-649 | 2 | 702463 | 4 | 501739 | 4 | 702461 | 2 | 501741 | 0,8190 |
| miR-650 | 2 | 702463 | 4 | 501739 | 4 | 702461 | 2 | 501741 | 0,8190 |
| miR-653 | 2 | 702463 | 4 | 501739 | 4 | 702461 | 2 | 501741 | 0,8190 |
| miR-657 | 2 | 702463 | 4 | 501739 | 4 | 702461 | 2 | 501741 | 0,8190 |

| | | | | | | | | | |
|---|---|---|---|---|---|---|---|---|---|
| miR-658 | 2 | 702463 | 4 | 501739 | 4 | 702461 | 2 | 501741 | 0,8190 |
| miR-659 | 2 | 702463 | 4 | 501739 | 4 | 702461 | 2 | 501741 | 0,8190 |
| miR-661 | 2 | 702463 | 4 | 501739 | 4 | 702461 | 2 | 501741 | 0,8190 |
| miR-662 | 2 | 702463 | 4 | 501739 | 4 | 702461 | 2 | 501741 | 0,8190 |
| miR-663 | 2 | 702463 | 4 | 501739 | 4 | 702461 | 2 | 501741 | 0,8190 |
| miR-663b | 2 | 702463 | 4 | 501739 | 4 | 702461 | 2 | 501741 | 0,8190 |
| miR-676* | 2 | 702463 | 4 | 501739 | 4 | 702461 | 2 | 501741 | 0,8190 |
| miR-711 | 2 | 702463 | 4 | 501739 | 4 | 702461 | 2 | 501741 | 0,8190 |
| miR-718 | 2 | 702463 | 4 | 501739 | 4 | 702461 | 2 | 501741 | 0,8190 |
| miR-7-2* | 2 | 702463 | 4 | 501739 | 4 | 702461 | 2 | 501741 | 0,8190 |
| miR-720 | 2 | 702463 | 4 | 501739 | 4 | 702461 | 2 | 501741 | 0,8190 |
| miR-759 | 2 | 702463 | 4 | 501739 | 4 | 702461 | 2 | 501741 | 0,8190 |
| miR-761 | 2 | 702463 | 4 | 501739 | 4 | 702461 | 2 | 501741 | 0,8190 |
| miR-762 | 2 | 702463 | 4 | 501739 | 4 | 702461 | 2 | 501741 | 0,8190 |
| miR-764 | 2 | 702463 | 4 | 501739 | 4 | 702461 | 2 | 501741 | 0,8190 |
| miR-802 | 2 | 702463 | 4 | 501739 | 4 | 702461 | 2 | 501741 | 0,8190 |
| miR-875-3p | 2 | 702463 | 4 | 501739 | 4 | 702461 | 2 | 501741 | 0,8190 |
| miR-885-3p | 2 | 702463 | 4 | 501739 | 4 | 702461 | 2 | 501741 | 0,8190 |
| miR-888* | 2 | 702463 | 4 | 501739 | 4 | 702461 | 2 | 501741 | 0,8190 |
| miR-892a | 2 | 702463 | 4 | 501739 | 4 | 702461 | 2 | 501741 | 0,8190 |
| miR-892b | 2 | 702463 | 4 | 501739 | 4 | 702461 | 2 | 501741 | 0,8190 |
| miR-920 | 2 | 702463 | 4 | 501739 | 4 | 702461 | 2 | 501741 | 0,8190 |
| miR-921 | 2 | 702463 | 4 | 501739 | 4 | 702461 | 2 | 501741 | 0,8190 |
| miR-922 | 2 | 702463 | 4 | 501739 | 4 | 702461 | 2 | 501741 | 0,8190 |
| miR-924 | 2 | 702463 | 4 | 501739 | 4 | 702461 | 2 | 501741 | 0,8190 |
| miR-933 | 2 | 702463 | 4 | 501739 | 4 | 702461 | 2 | 501741 | 0,8190 |
| miR-934 | 2 | 702463 | 4 | 501739 | 4 | 702461 | 2 | 501741 | 0,8190 |
| miR-936 | 2 | 702463 | 4 | 501739 | 4 | 702461 | 2 | 501741 | 0,8190 |
| miR-937 | 2 | 702463 | 4 | 501739 | 4 | 702461 | 2 | 501741 | 0,8190 |
| miR-938 | 2 | 702463 | 4 | 501739 | 4 | 702461 | 2 | 501741 | 0,8190 |

| | | | | | | | | | |
|---|---|---|---|---|---|---|---|---|---|
| miR-943 | 2 | 702463 | 4 | 501739 | 4 | 702461 | 2 | 501741 | 0,8190 |
| miR-887 | 17 | 702448 | 7 | 501736 | 14 | 702451 | 10 | 501733 | 0,8191 |
| miR-421 | 95 | 702370 | 55 | 501688 | 88 | 702377 | 62 | 501681 | 0,8191 |
| miR-212 | 10 | 702455 | 12 | 501731 | 13 | 702452 | 9 | 501734 | 0,8264 |
| miR-450b-3p | 10 | 702455 | 12 | 501731 | 13 | 702452 | 9 | 501734 | 0,8264 |
| miR-34b | 15 | 702450 | 6 | 501737 | 12 | 702453 | 9 | 501734 | 0,8299 |
| miR-769-5p | 29 | 702436 | 14 | 501729 | 25 | 702440 | 18 | 501725 | 0,8324 |
| miR-27a* | 9 | 702456 | 11 | 501732 | 12 | 702453 | 8 | 501735 | 0,8332 |
| miR-146b-3p | 3 | 702462 | 5 | 501738 | 5 | 702460 | 3 | 501740 | 0,8392 |
| miR-16-1* | 3 | 702462 | 5 | 501738 | 5 | 702460 | 3 | 501740 | 0,8392 |
| miR-219-5p | 3 | 702462 | 5 | 501738 | 5 | 702460 | 3 | 501740 | 0,8392 |
| miR-3130-5p | 3 | 702462 | 5 | 501738 | 5 | 702460 | 3 | 501740 | 0,8392 |
| miR-374c | 3 | 702462 | 5 | 501738 | 5 | 702460 | 3 | 501740 | 0,8392 |
| miR-582-3p | 3 | 702462 | 5 | 501738 | 5 | 702460 | 3 | 501740 | 0,8392 |
| miR-769-3p | 3 | 702462 | 5 | 501738 | 5 | 702460 | 3 | 501740 | 0,8392 |
| miR-876-3p | 3 | 702462 | 5 | 501738 | 5 | 702460 | 3 | 501740 | 0,8392 |
| miR-92a-2* | 3 | 702462 | 5 | 501738 | 5 | 702460 | 3 | 501740 | 0,8392 |
| miR-105* | 13 | 702452 | 5 | 501738 | 11 | 702454 | 7 | 501736 | 0,8392 |
| miR-215 | 22 | 702443 | 10 | 501733 | 19 | 702446 | 13 | 501730 | 0,8392 |
| hsa-let-7f-1* | 105 | 702360 | 62 | 501681 | 97 | 702368 | 70 | 501673 | 0,8413 |
| miR-19b-2* | 4 | 702461 | 6 | 501737 | 6 | 702459 | 4 | 501739 | 0,8471 |
| miR-216a | 4 | 702461 | 6 | 501737 | 6 | 702459 | 4 | 501739 | 0,8471 |
| miR-2278 | 4 | 702461 | 6 | 501737 | 6 | 702459 | 4 | 501739 | 0,8471 |
| miR-3611 | 6 | 702459 | 8 | 501735 | 8 | 702457 | 6 | 501737 | 0,8477 |
| miR-3615 | 6 | 702459 | 8 | 501735 | 8 | 702457 | 6 | 501737 | 0,8477 |
| miR-431 | 6 | 702459 | 8 | 501735 | 8 | 702457 | 6 | 501737 | 0,8477 |
| miR-592 | 6 | 702459 | 8 | 501735 | 8 | 702457 | 6 | 501737 | 0,8477 |
| miR-143* | 17 | 702448 | 18 | 501725 | 20 | 702445 | 15 | 501728 | 0,8490 |
| miR-224 | 41 | 702424 | 38 | 501705 | 46 | 702419 | 33 | 501710 | 0,8535 |
| miR-376a | 1798 | 700667 | 1231 | 500512 | 1767 | 700698 | 1262 | 500481 | 0,8591 |

| | | | | | | | | | |
|---|---|---|---|---|---|---|---|---|---|
| miR-130a | 3059 | 699406 | 2254 | 499489 | 3099 | 699366 | 2214 | 499529 | 0,8676 |
| miR-651 | 28 | 702437 | 27 | 501716 | 32 | 702433 | 23 | 501720 | 0,8702 |
| miR-656 | 39 | 702426 | 36 | 501707 | 44 | 702421 | 31 | 501712 | 0,8718 |
| miR-129-3p | 26 | 702439 | 13 | 501730 | 23 | 702442 | 16 | 501727 | 0,8920 |
| miR-422a | 12 | 702453 | 13 | 501730 | 15 | 702450 | 10 | 501733 | 0,8945 |
| miR-328 | 58 | 702407 | 33 | 501710 | 53 | 702412 | 38 | 501705 | 0,8954 |
| miR-149* | 12 | 702453 | 5 | 501738 | 10 | 702455 | 7 | 501736 | 0,9021 |
| miR-26a-1* | 10 | 702455 | 11 | 501732 | 12 | 702453 | 9 | 501734 | 0,9110 |
| hsa-let-7c* | 10 | 702455 | 4 | 501739 | 8 | 702457 | 6 | 501737 | 0,9117 |
| miR-3605-5p | 10 | 702455 | 4 | 501739 | 8 | 702457 | 6 | 501737 | 0,9117 |
| miR-3916 | 10 | 702455 | 4 | 501739 | 8 | 702457 | 6 | 501737 | 0,9117 |
| miR-670 | 10 | 702455 | 4 | 501739 | 8 | 702457 | 6 | 501737 | 0,9117 |
| miR-765 | 10 | 702455 | 4 | 501739 | 8 | 702457 | 6 | 501737 | 0,9117 |
| miR-34a* | 35 | 702430 | 19 | 501724 | 32 | 702433 | 22 | 501721 | 0,9198 |
| miR-26a-2* | 93 | 702372 | 77 | 501666 | 99 | 702366 | 71 | 501672 | 0,9215 |
| miR-628-3p | 8 | 702457 | 9 | 501734 | 10 | 702455 | 7 | 501736 | 0,9261 |
| miR-320c | 15 | 702450 | 15 | 501728 | 18 | 702447 | 12 | 501731 | 0,9306 |
| miR-301a | 91 | 702374 | 75 | 501668 | 97 | 702368 | 69 | 501674 | 0,9325 |
| miR-10a* | 7 | 702458 | 8 | 501735 | 9 | 702456 | 6 | 501737 | 0,9330 |
| miR-1255a | 6 | 702459 | 7 | 501736 | 8 | 702457 | 5 | 501738 | 0,9393 |
| miR-181c* | 6 | 702459 | 7 | 501736 | 8 | 702457 | 5 | 501738 | 0,9393 |
| miR-891a | 6 | 702459 | 7 | 501736 | 8 | 702457 | 5 | 501738 | 0,9393 |
| miR-766 | 23 | 702442 | 12 | 501731 | 20 | 702445 | 15 | 501728 | 0,9405 |
| miR-380* | 5 | 702460 | 6 | 501737 | 6 | 702459 | 5 | 501738 | 0,9449 |
| miR-488* | 5 | 702460 | 6 | 501737 | 6 | 702459 | 5 | 501738 | 0,9449 |
| miR-28-3p | 789 | 701676 | 537 | 501206 | 774 | 701691 | 552 | 501191 | 0,9456 |
| miR-433 | 80 | 702385 | 49 | 501694 | 75 | 702390 | 54 | 501689 | 0,9489 |
| miR-1250 | 4 | 702461 | 5 | 501738 | 5 | 702460 | 4 | 501739 | 0,9495 |
| miR-2116 | 4 | 702461 | 5 | 501738 | 5 | 702460 | 4 | 501739 | 0,9495 |
| miR-3179 | 4 | 702461 | 5 | 501738 | 5 | 702460 | 4 | 501739 | 0,9495 |

| | | | | | | | | | |
|---|---|---|---|---|---|---|---|---|---|
| miR-3190 | 4 | 702461 | 5 | 501738 | 5 | 702460 | 4 | 501739 | 0,9495 |
| miR-3199 | 4 | 702461 | 5 | 501738 | 5 | 702460 | 4 | 501739 | 0,9495 |
| miR-3200-5p | 4 | 702461 | 5 | 501738 | 5 | 702460 | 4 | 501739 | 0,9495 |
| miR-3622a-5p | 4 | 702461 | 5 | 501738 | 5 | 702460 | 4 | 501739 | 0,9495 |
| miR-548q | 4 | 702461 | 5 | 501738 | 5 | 702460 | 4 | 501739 | 0,9495 |
| miR-616 | 4 | 702461 | 5 | 501738 | 5 | 702460 | 4 | 501739 | 0,9495 |
| miR-100* | 11 | 702454 | 5 | 501738 | 9 | 702456 | 7 | 501736 | 0,9496 |
| miR-105 | 21 | 702444 | 11 | 501732 | 19 | 702446 | 13 | 501730 | 0,9513 |
| miR-1224-3p | 3 | 702462 | 4 | 501739 | 4 | 702461 | 3 | 501740 | 0,9526 |
| miR-1254 | 3 | 702462 | 4 | 501739 | 4 | 702461 | 3 | 501740 | 0,9526 |
| miR-1262 | 3 | 702462 | 4 | 501739 | 4 | 702461 | 3 | 501740 | 0,9526 |
| miR-187* | 3 | 702462 | 4 | 501739 | 4 | 702461 | 3 | 501740 | 0,9526 |
| miR-194* | 3 | 702462 | 4 | 501739 | 4 | 702461 | 3 | 501740 | 0,9526 |
| miR-200b* | 3 | 702462 | 4 | 501739 | 4 | 702461 | 3 | 501740 | 0,9526 |
| miR-222* | 3 | 702462 | 4 | 501739 | 4 | 702461 | 3 | 501740 | 0,9526 |
| miR-2277-5p | 3 | 702462 | 4 | 501739 | 4 | 702461 | 3 | 501740 | 0,9526 |
| miR-3118 | 3 | 702462 | 4 | 501739 | 4 | 702461 | 3 | 501740 | 0,9526 |
| miR-3125 | 3 | 702462 | 4 | 501739 | 4 | 702461 | 3 | 501740 | 0,9526 |
| miR-3163 | 3 | 702462 | 4 | 501739 | 4 | 702461 | 3 | 501740 | 0,9526 |
| miR-3177 | 3 | 702462 | 4 | 501739 | 4 | 702461 | 3 | 501740 | 0,9526 |
| miR-3616-3p | 3 | 702462 | 4 | 501739 | 4 | 702461 | 3 | 501740 | 0,9526 |
| miR-3621 | 3 | 702462 | 4 | 501739 | 4 | 702461 | 3 | 501740 | 0,9526 |
| miR-3622a-3p | 3 | 702462 | 4 | 501739 | 4 | 702461 | 3 | 501740 | 0,9526 |
| miR-3663-3p | 3 | 702462 | 4 | 501739 | 4 | 702461 | 3 | 501740 | 0,9526 |
| miR-3692* | 3 | 702462 | 4 | 501739 | 4 | 702461 | 3 | 501740 | 0,9526 |
| miR-3936 | 3 | 702462 | 4 | 501739 | 4 | 702461 | 3 | 501740 | 0,9526 |
| miR-561 | 3 | 702462 | 4 | 501739 | 4 | 702461 | 3 | 501740 | 0,9526 |
| miR-496 | 43 | 702422 | 25 | 501718 | 40 | 702425 | 28 | 501715 | 0,9547 |
| miR-502-3p | 69 | 702396 | 57 | 501686 | 74 | 702391 | 52 | 501691 | 0,9560 |
| miR-3151 | 9 | 702456 | 4 | 501739 | 8 | 702457 | 5 | 501738 | 0,9591 |

| | | | | | | | | | |
|---|---|---|---|---|---|---|---|---|---|
| miR-873 | 9 | 702456 | 4 | 501739 | 8 | 702457 | 5 | 501738 | 0,9591 |
| miR-30c-1* | 46 | 702419 | 39 | 501704 | 50 | 702415 | 35 | 501708 | 0,9606 |
| miR-3612 | 14 | 702451 | 7 | 501736 | 12 | 702453 | 9 | 501734 | 0,9631 |
| miR-1228* | 10 | 702455 | 10 | 501733 | 12 | 702453 | 8 | 501735 | 0,9662 |
| miR-23b* | 9 | 702456 | 9 | 501734 | 11 | 702454 | 7 | 501736 | 0,9721 |
| miR-1180 | 224 | 702241 | 172 | 501571 | 231 | 702234 | 165 | 501578 | 0,9725 |
| miR-361-5p | 532 | 701933 | 362 | 501381 | 522 | 701943 | 372 | 501371 | 0,9728 |
| miR-545* | 14 | 702451 | 13 | 501730 | 16 | 702449 | 11 | 501732 | 0,9767 |
| miR-3913 | 8 | 702457 | 8 | 501735 | 9 | 702456 | 7 | 501736 | 0,9775 |
| miR-511 | 8 | 702457 | 8 | 501735 | 9 | 702456 | 7 | 501736 | 0,9775 |
| miR-641 | 10 | 702455 | 5 | 501738 | 9 | 702456 | 6 | 501737 | 0,9801 |
| miR-181a* | 47 | 702418 | 29 | 501714 | 44 | 702421 | 32 | 501711 | 0,9837 |
| miR-668 | 8 | 702457 | 4 | 501739 | 7 | 702458 | 5 | 501738 | 0,9869 |
| miR-195 | 3021 | 699444 | 2123 | 499620 | 3001 | 699464 | 2143 | 499600 | 0,9877 |
| miR-1271 | 21 | 702444 | 18 | 501725 | 23 | 702442 | 16 | 501727 | 0,9883 |
| miR-15a* | 16 | 702449 | 14 | 501729 | 18 | 702447 | 12 | 501731 | 0,9892 |
| miR-3130-3p | 16 | 702449 | 14 | 501729 | 18 | 702447 | 12 | 501731 | 0,9892 |
| hsa-let-7a-2* | 19 | 702446 | 11 | 501732 | 18 | 702447 | 12 | 501731 | 0,9893 |
| miR-489 | 5 | 702460 | 5 | 501738 | 6 | 702459 | 4 | 501739 | 0,9907 |
| miR-744* | 5 | 702460 | 5 | 501738 | 6 | 702459 | 4 | 501739 | 0,9907 |
| miR-33a | 43 | 702422 | 27 | 501716 | 41 | 702424 | 29 | 501714 | 0,9913 |
| miR-1197 | 15 | 702450 | 13 | 501730 | 16 | 702449 | 12 | 501731 | 0,9922 |
| miR-3184 | 10 | 702455 | 9 | 501734 | 11 | 702454 | 8 | 501735 | 0,9926 |
| miR-193a-5p | 134 | 702331 | 102 | 501641 | 138 | 702327 | 98 | 501645 | 0,9936 |
| miR-940 | 36 | 702429 | 29 | 501714 | 38 | 702427 | 27 | 501716 | 0,9937 |
| miR-124* | 4 | 702461 | 4 | 501739 | 5 | 702460 | 3 | 501740 | 0,9939 |
| miR-1243 | 4 | 702461 | 4 | 501739 | 5 | 702460 | 3 | 501740 | 0,9939 |
| miR-1253 | 4 | 702461 | 4 | 501739 | 5 | 702460 | 3 | 501740 | 0,9939 |
| miR-1258 | 4 | 702461 | 4 | 501739 | 5 | 702460 | 3 | 501740 | 0,9939 |
| miR-1273d | 4 | 702461 | 4 | 501739 | 5 | 702460 | 3 | 501740 | 0,9939 |

| | | | | | | | | | |
|---|---|---|---|---|---|---|---|---|---|
| miR-129* | 4 | 702461 | 4 | 501739 | 5 | 702460 | 3 | 501740 | 0,9939 |
| miR-1295 | 4 | 702461 | 4 | 501739 | 5 | 702460 | 3 | 501740 | 0,9939 |
| miR-1914* | 4 | 702461 | 4 | 501739 | 5 | 702460 | 3 | 501740 | 0,9939 |
| miR-211 | 4 | 702461 | 4 | 501739 | 5 | 702460 | 3 | 501740 | 0,9939 |
| miR-3160 | 4 | 702461 | 4 | 501739 | 5 | 702460 | 3 | 501740 | 0,9939 |
| miR-3175 | 4 | 702461 | 4 | 501739 | 5 | 702460 | 3 | 501740 | 0,9939 |
| miR-3607-5p | 4 | 702461 | 4 | 501739 | 5 | 702460 | 3 | 501740 | 0,9939 |
| miR-363* | 4 | 702461 | 4 | 501739 | 5 | 702460 | 3 | 501740 | 0,9939 |
| miR-466 | 4 | 702461 | 4 | 501739 | 5 | 702460 | 3 | 501740 | 0,9939 |
| miR-548k | 4 | 702461 | 4 | 501739 | 5 | 702460 | 3 | 501740 | 0,9939 |
| miR-551b | 4 | 702461 | 4 | 501739 | 5 | 702460 | 3 | 501740 | 0,9939 |
| miR-570 | 4 | 702461 | 4 | 501739 | 5 | 702460 | 3 | 501740 | 0,9939 |
| miR-636 | 4 | 702461 | 4 | 501739 | 5 | 702460 | 3 | 501740 | 0,9939 |
| miR-770-5p | 4 | 702461 | 4 | 501739 | 5 | 702460 | 3 | 501740 | 0,9939 |
| miR-888 | 4 | 702461 | 4 | 501739 | 5 | 702460 | 3 | 501740 | 0,9939 |
| miR-186* | 9 | 702456 | 5 | 501738 | 8 | 702457 | 6 | 501737 | 0,9951 |
| miR-3154 | 9 | 702456 | 5 | 501738 | 8 | 702457 | 6 | 501737 | 0,9951 |
| miR-548w | 9 | 702456 | 8 | 501735 | 10 | 702455 | 7 | 501736 | 0,9952 |
| miR-188-5p | 43 | 702422 | 34 | 501709 | 45 | 702420 | 32 | 501711 | 0,9955 |
| miR-381 | 297 | 702168 | 204 | 501539 | 292 | 702173 | 209 | 501534 | 0,9960 |
| miR-584 | 12 | 702453 | 7 | 501736 | 11 | 702454 | 8 | 501735 | 0,9961 |
| miR-2355-3p | 18 | 702447 | 11 | 501732 | 17 | 702448 | 12 | 501731 | 0,9967 |
| miR-1248 | 8 | 702457 | 7 | 501736 | 9 | 702456 | 6 | 501737 | 0,9972 |
| miR-203 | 12 | 702453 | 10 | 501733 | 13 | 702452 | 9 | 501734 | 0,9980 |
| miR-676 | 7 | 702458 | 4 | 501739 | 6 | 702459 | 5 | 501738 | 0,9981 |
| miR-758 | 161 | 702304 | 120 | 501623 | 164 | 702301 | 117 | 501626 | 0,9981 |
| miR-589* | 10 | 702455 | 6 | 501737 | 9 | 702456 | 7 | 501736 | 0,9984 |
| miR-1285 | 19 | 702446 | 12 | 501731 | 18 | 702447 | 13 | 501730 | 0,9985 |
| miR-500a* | 43 | 702422 | 33 | 501710 | 44 | 702421 | 32 | 501711 | 0,9989 |
| miR-99b* | 45 | 702420 | 30 | 501713 | 44 | 702421 | 31 | 501712 | 0,9991 |

| | | | | | | | | | |
|---|---|---|---|---|---|---|---|---|---|
| hsa-let-7f-2* | 19 | 702446 | 15 | 501728 | 20 | 702445 | 14 | 501729 | 0,9991 |
| miR-767-5p | 19 | 702446 | 15 | 501728 | 20 | 702445 | 14 | 501729 | 0,9991 |
| miR-545 | 17 | 702448 | 11 | 501732 | 16 | 702449 | 12 | 501731 | 0,9995 |
| miR-3679-5p | 6 | 702459 | 5 | 501738 | 6 | 702459 | 5 | 501738 | 0,9995 |
| miR-431* | 6 | 702459 | 5 | 501738 | 6 | 702459 | 5 | 501738 | 0,9995 |
| miR-890 | 6 | 702459 | 5 | 501738 | 6 | 702459 | 5 | 501738 | 0,9995 |
| miR-337-5p | 18 | 702447 | 14 | 501729 | 19 | 702446 | 13 | 501730 | 0,9996 |
| miR-708* | 10 | 702455 | 8 | 501735 | 11 | 702454 | 7 | 501736 | 0,9996 |
| miR-944 | 11 | 702454 | 7 | 501736 | 11 | 702454 | 7 | 501736 | 0,9996 |
| miR-483-3p | 43 | 702422 | 29 | 501714 | 42 | 702423 | 30 | 501713 | 0,9996 |
| miR-1299 | 8 | 702457 | 5 | 501738 | 8 | 702457 | 5 | 501738 | 0,9996 |
| miR-150* | 8 | 702457 | 5 | 501738 | 8 | 702457 | 5 | 501738 | 0,9996 |
| miR-206 | 8 | 702457 | 5 | 501738 | 8 | 702457 | 5 | 501738 | 0,9996 |
| miR-432* | 8 | 702457 | 5 | 501738 | 8 | 702457 | 5 | 501738 | 0,9996 |
| miR-132* | 90 | 702375 | 62 | 501681 | 89 | 702376 | 63 | 501680 | 0,9997 |
| miR-574-3p | 969 | 701496 | 698 | 501045 | 972 | 701493 | 695 | 501048 | 0,9999 |
| miR-1266 | 5 | 702460 | 4 | 501739 | 5 | 702460 | 4 | 501739 | 0,9999 |
| miR-1267 | 5 | 702460 | 4 | 501739 | 5 | 702460 | 4 | 501739 | 0,9999 |
| miR-3605-3p | 5 | 702460 | 4 | 501739 | 5 | 702460 | 4 | 501739 | 0,9999 |
| miR-3661 | 5 | 702460 | 4 | 501739 | 5 | 702460 | 4 | 501739 | 0,9999 |
| miR-939 | 5 | 702460 | 4 | 501739 | 5 | 702460 | 4 | 501739 | 0,9999 |
| miR-500a | 38 | 702427 | 26 | 501717 | 37 | 702428 | 27 | 501716 | 0,9999 |
| miR-331-5p | 22 | 702443 | 15 | 501728 | 22 | 702443 | 15 | 501728 | 1,0000 |
| miR-541* | 6 | 702459 | 4 | 501739 | 6 | 702459 | 4 | 501739 | 1,0000 |
| miR-654-5p | 6 | 702459 | 4 | 501739 | 6 | 702459 | 4 | 501739 | 1,0000 |
| miR-330-3p | 16 | 702449 | 11 | 501732 | 16 | 702449 | 11 | 501732 | 1,0000 |
| miR-671-3p | 8 | 702457 | 6 | 501737 | 8 | 702457 | 6 | 501737 | 1,0000 |
| miR-103-2* | 15 | 702450 | 11 | 501732 | 15 | 702450 | 11 | 501732 | 1,0000 |
| miR-877 | 37 | 702428 | 26 | 501717 | 37 | 702428 | 26 | 501717 | 1,0000 |
| miR-136* | 193 | 702272 | 137 | 501606 | 193 | 702272 | 137 | 501606 | 1,0000 |

| | | | | | | | | | |
|---|---|---|---|---|---|---|---|---|---|
| miR-29a* | 283 | 702182 | 203 | 501540 | 284 | 702181 | 202 | 501541 | 1,0000 |
| miR-452 | 11 | 702454 | 8 | 501735 | 11 | 702454 | 8 | 501735 | 1,0000 |
| miR-1294 | 7 | 702458 | 5 | 501738 | 7 | 702458 | 5 | 501738 | 1,0000 |
| miR-3065-5p | 7 | 702458 | 5 | 501738 | 7 | 702458 | 5 | 501738 | 1,0000 |

# P-values were calculated using $\chi 2$ test; $P<0.0001$ is considered as significant

**Supplementary Table 3.** Sequencing-based SAM results for differentially expressed miRNAs between TGCTs and NT

| miRNA | Score (d) | Fold change (log2) | FDR (q-value) |
|---|---|---|---|
| **Over-expressed miRNAs in TGCTs** | | | |
| miR-223 | 1,2 | 2,0 | 72,4 |
| miR-15a | 1,2 | 1,8 | 72,4 |
| miR-21 | 1,1 | 2,1 | 72,8 |
| miR-92a | 1,0 | 1,8 | 75,0 |
| miR-15b | 0,9 | 1,7 | 75,0 |
| miR-27a | 0,9 | 1,8 | 75,0 |
| miR-142-3p | 0,9 | 2,2 | 77,7 |
| miR-19b | 0,9 | 1,7 | 77,7 |
| miR-25 | 0,9 | 1,8 | 77,7 |
| miR-96 | 0,9 | 2,0 | 77,7 |
| miR-374a | 0,8 | 1,9 | 82,3 |
| miR-302b* | 0,8 | 1,9 | 82,3 |
| miR-372 | 0,8 | 2,0 | 82,3 |
| miR-302b | 0,8 | 2,2 | 82,3 |
| miR-183 | 0,8 | 1,7 | 82,3 |
| miR-367 | 0,8 | 2,1 | 82,3 |
| miR-142-5p | 0,8 | 2,0 | 82,3 |
| miR-182 | 0,8 | 2,1 | 82,3 |
| miR-515-5p | 0,8 | 2,0 | 82,3 |
| miR-19a | 0,8 | 1,9 | 82,3 |
| miR-7 | 0,7 | 1,8 | 82,5 |
| miR-16 | 0,7 | 1,6 | 82,5 |
| miR-302a | 0,7 | 2,2 | 83,3 |
| **Under-expressed miRNAs in TGCTs** | | | |
| miR-202 | -8,3 | 0,0 | 0,0 |
| miR-507 | -6,3 | 0,1 | 0,0 |
| miR-510 | -5,3 | 0,0 | 0,0 |
| miR-513c | -4,1 | 0,1 | 0,0 |
| miR-514 | -4,1 | 0,0 | 0,0 |
| miR-424* | -3,9 | 0,1 | 0,0 |
| miR-508-5p | -3,8 | 0,1 | 0,0 |
| miR-513b | -2,7 | 0,0 | 0,0 |
| miR-542-5p | -2,5 | 0,2 | 0,0 |
| miR-508-3p | -2,5 | 0,0 | 0,0 |
| miR-202* | -2,5 | 0,1 | 0,0 |
| miR-134 | -2,5 | 0,2 | 0,0 |
| miR-503 | -2,5 | 0,2 | 0,0 |
| miR-509-3p | -2,4 | 0,1 | 0,0 |
| miR-424 | -2,3 | 0,2 | 6,2 |
| miR-506 | -2,0 | 0,2 | 18,7 |
| miR-129-5p | -1,9 | 0,1 | 18,7 |
| miR-193b* | -1,9 | 0,2 | 18,7 |
| miR-204 | -1,9 | 0,2 | 18,7 |
| miR-483-5p | -1,9 | 0,3 | 18,7 |

| | | | |
|---|---|---|---|
| miR-99a* | -1,7 | 0,2 | 26,7 |
| miR-509-3-5p | -1,6 | 0,3 | 32,5 |
| hsa-let-7b | -1,6 | 0,2 | 32,5 |
| miR-494 | -1,5 | 0,4 | 36,0 |
| hsa-let-7c | -1,5 | 0,2 | 36,0 |
| miR-513a-3p | -1,5 | 0,3 | 36,0 |
| miR-10b* | -1,4 | 0,3 | 41,6 |
| miR-324-5p | -1,3 | 0,5 | 50,1 |
| miR-23a* | -1,3 | 0,3 | 50,1 |
| miR-25* | -1,3 | 0,5 | 50,1 |
| miR-29b-2* | -1,2 | 0,4 | 50,1 |
| miR-542-3p | -1,2 | 0,5 | 50,1 |
| miR-485-5p | -1,2 | 0,4 | 50,1 |
| miR-210 | -1,2 | 0,4 | 50,1 |
| miR-411 | -1,2 | 0,5 | 50,1 |
| miR-487b | -1,1 | 0,5 | 50,1 |
| miR-2110 | -1,1 | 0,3 | 50,1 |
| miR-874 | -1,1 | 0,4 | 56,1 |
| miR-320a | -1,0 | 0,4 | 56,1 |
| miR-214 | -1,0 | 0,4 | 56,1 |
| miR-140-3p | -1,0 | 0,5 | 56,1 |
| miR-99a | -1,0 | 0,4 | 56,1 |
| miR-151-3p | -1,0 | 0,5 | 56,1 |
| miR-509-5p | -1,0 | 0,4 | 56,1 |
| miR-664* | -1,0 | 0,4 | 56,1 |
| miR-423-5p | -1,0 | 0,5 | 56,1 |
| miR-1908 | -0,9 | 0,4 | 56,1 |
| miR-362-5p | -0,9 | 0,4 | 56,1 |
| miR-127-3p | -0,9 | 0,6 | 56,1 |
| miR-449a | -0,9 | 0,4 | 56,1 |
| miR-514b-5p | -0,9 | 0,4 | 56,1 |
| miR-409-3p | -0,9 | 0,6 | 56,1 |
| miR-574-5p | -0,8 | 0,6 | 56,1 |
| miR-744 | -0,8 | 0,6 | 56,1 |
| miR-199a-5p | -0,8 | 0,6 | 56,1 |
| miR-450a | -0,8 | 0,6 | 56,1 |
| miR-193b | -0,8 | 0,6 | 56,1 |
| miR-370 | -0,8 | 0,5 | 56,1 |
| miR-125a-3p | -0,7 | 0,5 | 61,9 |
| miR-221 | -0,7 | 0,7 | 61,9 |
| miR-299-3p | -0,7 | 0,6 | 61,9 |
| miR-133a | -0,7 | 0,5 | 61,9 |
| miR-1296 | -0,6 | 0,5 | 61,9 |
| miR-642a | -0,6 | 0,5 | 61,9 |
| miR-525-3p | -0,6 | 0,6 | 61,9 |
| miR-517b | -0,6 | 0,6 | 61,9 |
| miR-200a | -0,6 | 0,6 | 61,9 |
| miR-520d-5p | -0,6 | 0,6 | 61,9 |
| miR-197 | -0,5 | 0,7 | 64,9 |
| miR-520e | -0,5 | 0,6 | 64,9 |
| hsa-let-7a | -0,5 | 0,6 | 64,9 |
| miR-127-5p | -0,5 | 0,7 | 64,9 |

| | | | |
|---|---|---|---|
| miR-181b | -0,5 | 0,7 | 64,9 |
| miR-642b | -0,5 | 0,6 | 64,9 |
| miR-362-3p | -0,5 | 0,7 | 64,9 |
| miR-521 | -0,5 | 0,6 | 64,9 |
| miR-375 | -0,5 | 0,6 | 64,9 |
| miR-1301 | -0,5 | 0,7 | 64,9 |
| miR-345 | -0,5 | 0,7 | 64,9 |
| miR-373* | -0,5 | 0,7 | 64,9 |
| miR-522 | -0,4 | 0,7 | 64,9 |
| miR-339-5p | -0,4 | 0,7 | 64,9 |
| miR-497 | -0,4 | 0,7 | 64,9 |
| hsa-let-7e | -0,4 | 0,7 | 64,9 |
| miR-302c* | -0,4 | 0,7 | 64,9 |
| miR-518d-3p | -0,4 | 0,7 | 68,5 |
| miR-660 | -0,4 | 0,8 | 68,5 |
| miR-125b | -0,4 | 0,7 | 68,5 |
| miR-135a | -0,3 | 0,7 | 68,5 |
| miR-26b* | -0,3 | 0,8 | 70,9 |
| miR-28-5p | -0,3 | 0,8 | 70,9 |
| miR-615-3p | -0,3 | 0,7 | 70,9 |
| miR-152 | -0,3 | 0,8 | 70,9 |
| miR-130b* | -0,3 | 0,8 | 70,9 |
| miR-92a-1* | -0,3 | 0,8 | 70,9 |
| miR-382 | -0,3 | 0,8 | 70,9 |
| miR-488 | -0,3 | 0,8 | 70,9 |
| miR-125a-5p | -0,3 | 0,8 | 70,9 |
| miR-1269 | -0,2 | 0,8 | 70,9 |
| miR-520g | -0,2 | 0,8 | 72,4 |
| miR-30b* | -0,2 | 0,8 | 72,4 |
| miR-411* | -0,2 | 0,9 | 72,4 |
| miR-548x | -0,2 | 0,8 | 72,4 |
| miR-523 | -0,2 | 0,9 | 72,4 |
| miR-520a-3p | -0,2 | 0,9 | 72,4 |

FDR = False discovery rate

**Supplementary Table 4.** Results from immunohistochemical analysis of p50 and PEG3 in TGCTs, non-germ cell testicular tumors, and testicular non-tumor tissues

| **Immunohistochemical marker** | | **Immunohistochemistry [Number (%)]** | | | |
|---|---|---|---|---|---|
| **Sample type** | **(n =)** | **Negative** | **Weak** | **Intermediate** | **Strong** |
| **p50 staining** | | | | | |
| ***TGCTs*** | **96** | **2 (2.1)** | **11 (11.5)** | **8 (8.3)** | **75 (78.1)** |
| Seminoma (classic, multinodular, anaplastic) | 66 | 2 (3.0) | 7 (10.6) | 7 (10.6) | 50 (75.8) |
| Embryonal carcinoma | 17 | 0 | 2 (11.8) | 1 (5.9) | 14 (82.4) |
| Yolk sac tumor | 7 | 0 | 0 | 0 | 7 (100 ) |
| Mixed germ cell tumor | 1 | 0 | 0 | 0 | 1 (100) |
| Teratoma | 2 | 0 | 1 (50) | 0 | 1 (50) |
| Choriocarcinoma | 1 | 0 | 0 | 0 | 1 (100) |
| IGCNU | 2 | 0 | 1 (50) | 0 | 1 (50) |
| ***Non-germ cell testicular tumors*** | **8** | **0** | **4 (50)** | **0** | **4 (50)** |
| Testis adenomatoid tumor | 1 | 0 | 0 | 0 | 1 (100) |
| Testis fibrous pseudotumor | 1 | 0 | 0 | 0 | 1 (100) |
| Leydig cell tumor | 1 | 0 | 0 | 0 | 1 (100) |
| Testis liposarcoma | 3 | 0 | 3 (100) | 0 | 0 |
| Testis papillary serous | 1 | 0 | 1 (100) | 0 | 0 |
| Testis adenocarcinoma | 1 | 0 | 0 | 0 | 1 (100) |
| ***Testicular non-tumor tissues*** | **25** | **1 (4)** | **8 (32)** | **3 (12)** | **13 (52)** |
| **PEG3 staining** | | | | | |
| ***TGCTs*** | **96** | **4 (4.2)** | **2 (2.1 )** | **10 (10.4)** | **80 (83.3)** |
| Seminoma (classic, multinodular, anaplastic) | 63 | 3 (4.8) | 2 (3.2 ) | 8 (12.7) | 50 (79.4) |
| Embryonal carcinoma | 18 | 0 | 0 | 0 | 18 (100) |
| Yolk sac tumor | 7 | 0 | 0 | 2 (28.6) | 5 (71.4) |
| Mixed germ cell tumor | 1 | 0 | 0 | 0 | 1 (100) |
| Teratoma | 4 | 1 (25) | 0 | 0 | 3 (75) |
| Choriocarcinoma | 1 | 0 | 0 | 0 | 1 (100) |
| IGCNU | 2 | 0 | 0 | 0 | 2 (100) |
| ***Non-germ cell testicular tumors*** | **8** | **0** | **5 (62.5)** | **0** | **3 (37.5)** |
| Testis adenomatoid tumor | 1 | 0 | 1 (100) | 0 | 0 |
| Testis fibrous pseudotumor | 1 | 0 | 1 (100) | 0 | 0 |
| Leydig cell tumor | 1 | 0 | 0 | 0 | 1 (100) |
| Testis liposarcoma | 3 | 0 | 2 (66.7 ) | 0 | 1 (33.3) |
| Testis papillary serous | 1 | 0 | 1 (100) | 0 | 0 |
| Testis adenocarcinoma | 1 | 0 | 0 | 0 | 1 (100) |
| ***Testicular non-tumor tissues*** | **25** | **0** | **12 (48)** | **3 (12)** | **10 (40)** |

IGCNU = intratubular germ cell neoplasia unclassified

Negative = All nuclei scored as having intensity 0

Weak = A mixture of nuclei scored as having intensity 0 and 1+, 2+ or 3+

Intermediate = All nuclei scored as intensity 1+ or a mixture of nuclei scored as 1+ and 2+ or 3+

Strong = A mixture of nuclei scored as having intensity 2+, 3+ or a mixture of nuclei with 2+ and 3+

**Supplementary Table 5.** Genotypes of the 2102Ep and TCam-2 cell lines for short tandem repeat (STR) markers

| **Locus** | **2102Ep** | | **TCam-2** | |
|---|---|---|---|---|
| **Publication** | **This study** | **Reference (17)** | **This study** | **Reference (17)** |
| *AMEL* | X, Y | X, Y | X, Y | X, Y |
| *D8S1179* | 14, 15 | - | 14, 17 | - |
| *D21S11* | 28, 29 | - | 30 | - |
| *D7S820* | 7 | 7, 13 | 8, 11 | 8, 11 |
| *CSF1PO* | 11, 12 | 11, 12 | 9, 11 | 9, 11 |
| *D3S1358* | 16 | - | 16, 17 | - |
| *THO1* | 9, 9.3 | 9, 9.3 | 6, 9 | 6, 9 |
| *D13S317* | 8 | 8 | 8, 12 | 8, 12 |
| *D16S539* | 11, 12 | 11, 12 | 9 | 9 |
| *D2S1338* | 17, 20 | - | 23 | - |
| *D19S433* | 13, 14 | - | 15 | - |
| *vWA* | 16, 17, 18 | 13, 16, 18 | 18, 19 | 18, 19 |
| *TPOX* | 8, 11 | 8, 11 | 9 | 9 |
| *D18S51* | 15 | - | 13 | - |
| *D5S818* | 11 | 11 | 11, 12 | 11, 12 |
| *FGA* | 22 | - | 25 | - |

Reference (17) Palmer *et al.* 2010.

**Supplementary Table 6.** List of the oligonucleotides used in the study

| Purpose, Name | Oligonucleotide Sequences (5' to 3') |
|---|---|
| **Small RNA cloning** | |
| 3'-adaptor ("Linker-1"; IDT) | /5rApp/CTGTAGGCACCATCAAT/3ddC |
| 5'-adaptor | ACGCTCTTCCGATCTv |
| RT | ATTGATGGTGCCTACAG |
| Amp_F | GATACGGCGACCACCGAGATCTACACTCTTTCCCTACACGACGCTCTTCCGATCT |
| Amp_R | CAAGCAGAAGACGGCATACGAGCTCTTCCGATCTATTGATGGTGCCTACAG |
| ***PEG3* 3'UTR luciferase reporter constructs** | |
| WT_sense | TTTATGATTATAACTGTGTCAAAtagtATTCTATCTGTAATAGTGTCAACtagtTAGGGCTTGGAAGTATG TCAAAGtagtTCAAAGATGCAGGGAGTGTCAAA |
| WT_antisense | TTTGACACTCCCTGCATCTTTGAactaCTTTGACATACTTCCAAGCCCTAactaGTTGACACTATTACAG ATAGAATactaTTTGACACAGTTATAATCATAAA |
| MUT_sense | TTTATGATTATAACTGTGCGAAtagtATTCTATCTGTAATAGTGTTACtagtTAGGGCTTGGAAGTATTGA AAGtagtTCAAAGATGCAGGGAGTGCGAA |
| MUT_antisense | TTCGCACTCCCTGCATCTTTGAactaCTTTCAATACTTCCAAGCCCTAactaGTAACACTATTACAGATA GAATactaTTCGCACAGTTATAATCATAAA |
| **shRNA constructs** | |
| shPEG3-1_ sense | GATCTGCCTAATCTATGTGGAATTTGTCGAAAAATTCCACATAGATTAGGTTTTTTTGGTAC |
| shPEG3-1_antisense | CAAAAAAACCTAATCTATGTGGAATTTTTCGACAAATTCCACATAGATTAGGCA |
| shPEG3-2_sense | GATCTGGCTCTAACCCCTATGAAAACCCGAATTTTCATAGGGGTTAGAGCTATTTTTGGTAC |
| shPEG3-2_antisense | CAAAAATAGCTCTAACCCCTATGAAAATTCGGGTTTTCATAGGGGTTAGAGCCA |
| **PEG3 CDS construct** | |
| PEG3-F | ATGCTGCCTCCAAAGCACTTGTC |
| PEG3-R | TCAGCCAGTGTGGGTATTCTGGT |
| **PEG3 Pyrosequencing*** | |
| PEG-SF | TGTCTTCTAGGCTGCTTGGTTCGTGCC |
| PEG3-F | CTCACAACACAATCCAGGAC |
| PEG3-R | TAGACCTCGACTGGTGCTTG |

v = barcodes with triple RNA molecules: aaa, ggg, ccc or uuu; * Primers were used in Ref. 40
